# Efficacy, Structure–Activity Relationship, and Mode of Action Studies of a New Generation of Acridine/Acridone-Based Antimalarials

**DOI:** 10.1021/acsinfecdis.5c00682

**Published:** 2026-05-26

**Authors:** Sarah El Chamy Maluf, Giovana Rossi Mendes, Igor M. R. Moura, Guilherme Eduardo de Souza, Talita Alvarenga Valdes, Vinícius Bonatto, Gabriela Silva Oliveira, Anna Caroline Campos Aguiar, Marcos L. Gazarini, Ana C. Puhl, Natalia Monakhova, Alexander Lepioshkin, Vadim Makarov, Thomas R. Lane, Renuka Raman, Guilherme A. S. Campolina, Camila S. Barbosa, Amália dos Santos Ferreira, Carolina B. G. Teles, Dhelio B. Pereira, Roberto Rudge de Moraes Barros, Ernest Diez Benavente, Sean Ekins, Rafael Victorio Carvalho Guido

**Affiliations:** a São Carlos Institute of Physics, University of São Paulo, São Carlos 13563-120, Brazil; b Department of Microbiology, Immunology and Parasitology, Federal University of São Paulo, São Paulo, CEP 04023-062, Brazil; c Department of Biosciences, Federal University of São Paulo, Santos 11015-020, Brazil; d Collaborations Pharmaceuticals, Inc., 840 Main Campus Drive, Lab 3510, Raleigh, North Carolina 27606, United States; e Research Center of Biotechnology RAS, 33-2 Leninsky prospect, Moscow 119071, Russia; f Oswaldo Cruz Foundation, Leishmaniasis and Malaria Bioassay Platform, Porto Velho 76812-245, Brazil; g Research Center in Tropical Medicine of Rondônia, Porto Velho 76812-329, Brazil; h Laboratory of Experimental Cardiology, University Medical Center Utrecht, 8125 Utrecht University, Utrecht, CS 3584, The Netherlands

## Abstract

Malaria continues to devastate humanity with significant global morbidity and mortality. The emerging resistance to antimalarials suggests artemisinin and its derivatives will encounter the same challenges as other antimalarial drugs. Strategies to accelerate developing new antimalarials include utilizing known scaffolds and making structural modifications to enhance properties and elucidate their mode of action. We have focused on acridine derivatives and describe 18 acridine/acridone analogs based on pyronaridine or quinacrine cores. Several molecules demonstrated potent *in vitro* activity against both chloroquine-sensitive strains of*Plasmodium falciparum*, with either no observed or low cytotoxicity. Additionally, we investigated the mode of action of these derivatives, their combination with other antimalarials, and their inhibitory activity against multidrug-resistant strains of*P. falciparum*. Compound **5a**, an acridone-based derivative, demonstrated lower potency against the atovaquone-resistant strain (IC_50_^*Pf*TM90C6B^ > 12.5 μM) and exhibited a slow-acting mechanism, along with inhibition of the mitochondrial bc1 complex (IC_50_^bc1^ = 2 μM). In contrast, compound **2d** (IC_50_^*Pf*3D7^ = 0.02 μM), an acridine-based derivative, showed fast-acting inhibition, localized near the parasite’s digestive vacuole, and inhibited hemozoin formation (IC_50_ = 5 μM). Acridine-based derivatives **2d** showed potent nanomolar inhibitory activity against *P. falciparum* and *P. vivax* field isolates and improved survival in mice infected with *P. berghei*, achieving a 100% survival rate at 30 days. These findings suggest that acridone- and acridine-based derivatives likely act through distinct modes of action, providing valuable insights for developing new antimalarials active against resistant strains of *P. falciparum* and demonstrating efficacy in both *ex vivo* and *in vivo* models.

## Graphical abstract

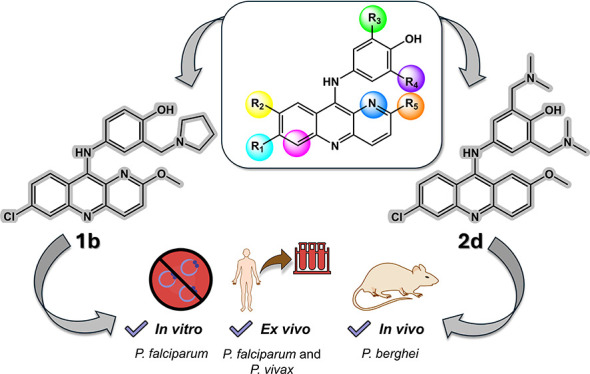

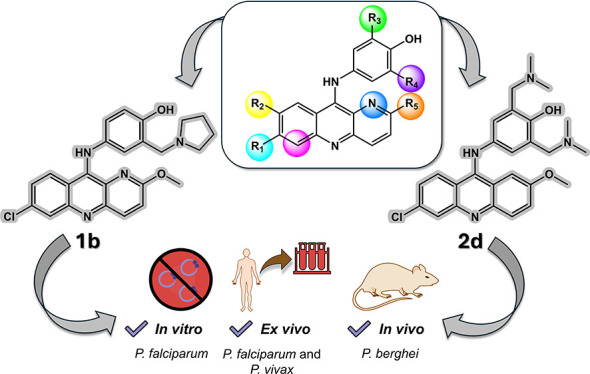

## Introduction

Malaria is one of the deadliest human pathogen-borne diseases, accounting for more than 282 million cases and 610,000 estimated deaths in 2024.^1^ Despite more than 200 species included in the *Plasmodium* genus, only six are known to cause malaria in humans (*P. falciparum*, *P. vivax*, *P. ovalecurtisi*, *P. ovalewallikeri*, *P. malariae*, and *P. knowlesi*).^2,3^

Insecticide-treated bed nets and artemisinin-based combination therapies (ACTs) have been the primary strategies for reducing the malaria burden over the past two decades.^1^ While ACTs are crucial components of treatment policies in numerous malaria-endemic countries, the emergence of resistance to their components poses a serious threat to malaria control, necessitating the development of new effective alternatives.^4,5^ Mosquito nets, although highly effective, face challenges such as incomplete coverage, inconsistent use, and the development of insecticide-resistant mosquito populations. The first approved malaria vaccine, Mosquirix (RTS, S/AS01),^6,7^ represents an important milestone but currently provides only partial protection in children and requires multiple doses for optimal efficacy. These limitations underscore the need for new antimalarials targeting novel mechanisms or repurposing existing scaffolds. In this sense, acridine- and acridone-based (AC) compounds represent a promising chemical series for investigation.^8^

Interest in acridine-based (AC) compounds as antimalarials began with the discovery that synthetic dyes like methylene blue (MB) and acridine orange (AO) inhibit *P. falciparum* at nanomolar concentrations.^8^ MB’s use was restricted due to side effects and toxicity, prompting the development of safer drugs. Quinacrine, synthesized in 1932,^9^ became the first synthetic antimalarial to undergo clinical trials and was widely used during World War II before being replaced by chloroquine for safety and efficacy reasons.^10^ As resistance to chloroquine spread, focus shifted to developing improved quinacrine derivatives with enhanced tolerability and multitargeted activity.^11–17^

Pyronaridine (Malaridine),^18^ an acridine-based drug synthesized in 1970,^19^ when used in combination with artesunate (Pyramax) is an effective and affordable treatment for uncomplicated malaria.^20^ Furthermore, pyronaridine exhibits antitumor,^21–23^ antiprotozoal,^24,25^ antiviral,^26^ and antibacterial^27^ activities.

The proposed mechanisms of action for AC-based antimalarials include binding to heme^16^ and inhibition of hematin polymerization.^28^ Additionally, inhibition of the mitochondrial bc1 complex has been suggested as another possible mechanism of action.^29^ We now describe 18 acridine/acridone-based compounds that were synthesized and evaluated against the malaria human parasite *P. falciparum* and *P. vivax* and the murine parasite *P. berghei*. Studies of the structure–activity relationship (SAR), mode of action, and combinations with other antimalarial drugs provide valuable information into the parasitological profile of this class of compounds.

## Results

### Antiplasmodial Activities and Structure–Activity Relationship

The compounds synthesized and assessed were separated into two series, the pyronaridine derivatives (**1a**–**3b**) and the quinacrine derivatives (**4a–5a**). These compounds were tested for hemolytic activity and showed no hemolytic effects at a concentration of 10 μM (Figure S1). For the pyronaridine derivatives (Table 1), three subseries (**1**–**3**) with different positions of the heteroatoms in the tricyclic system were synthesized, benzo­[*b*]-1,5-naphthyridine (**1**), acridine (**2**), and benzo­[*b*]-1,8-naphthyridine (**3**), while the substituents groups were somewhat like pyronaridine (**1a**).

**Table 1:**
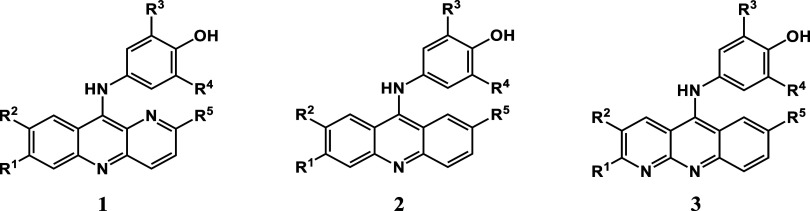

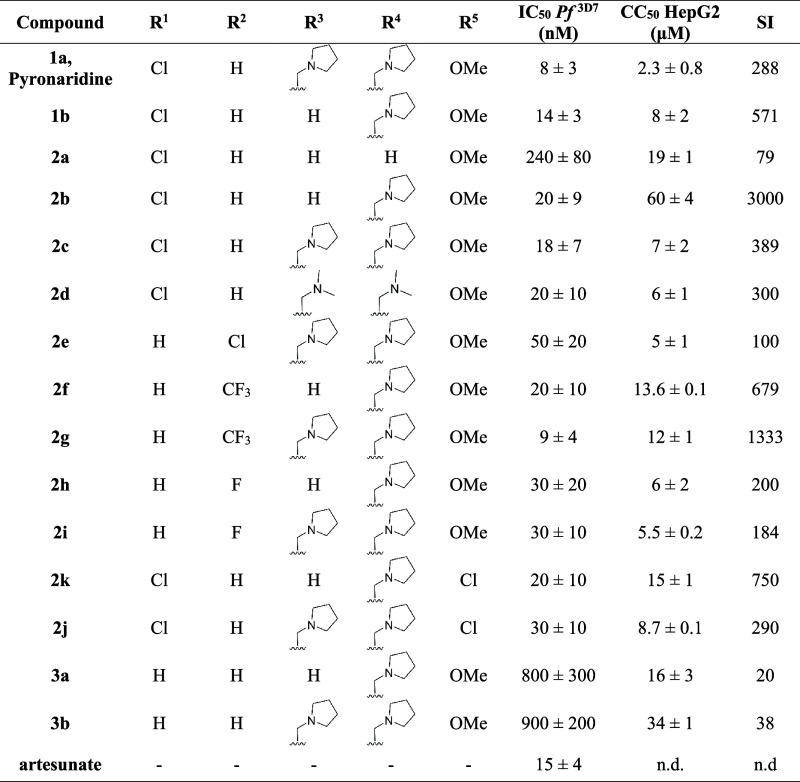
*In Vitro* Antiplasmodial Activity against Chloroquine-Sensitive (3D7) Strain of *P. falciparum*, Cytotoxicity against the HepG2 Cell Line, and Selectivity Index of Pyronaridine Derivatives^a^

a Mean values are represented with their respective standard deviations (n.d. = not determined).

Scaffolds **1** and **2** exhibited higher antiplasmodial activity compared to scaffold **3**, with their potency reaching up to 100-fold greater than scaffold **3**. The presence of an extra nitrogen atom in scaffold **1** led to compounds slightly more potent than acridine (**2**), as evidenced by the comparison between the **1a** → **2c** and **1b** → **2b**. An additional ring nitrogen can modulate basicity and electronic distribution and has been shown to increase potency in related heterocyclic antimalarial scaffolds.^30^ For example, reduced antiplasmodial activity was observed for chloroquine analogues lacking basic nitrogen in the quinoline scaffold.^31^

To investigate the influence of substituent groups in acridine-based compounds, scaffold **2** was selected. Investigations on the groups R_1_ and R_2_ showed that the Cl atom is favorable at the R^1^ position (e.g., **2c**), while shifting the Cl to the R^2^ position (**2e**) resulted in a loss of up to 3-fold in potency. However, when the Cl at R^2^ is replaced with an F atom (**2i**), the potency is nearly restored. The introduction of a trifluoromethyl group at the R^2^ position (**2g**) yielded the most potent new compound (after pyronaridine) (IC_50_ *Pf* ^3D7^= 9 nM) within all tested series with a selectivity index of 1333. This compound is equipotent to pyronaridine (**1a**, IC_50_ *Pf* ^3D7^= 8 nM), but its cytotoxicity is 5-fold lower.

Next, we evaluated the importance of the pyrrolidinylmethylene group at R^3^/R^4^. The removal of pyrrolidine groups at both positions resulted in the least potent compound within the series of benzo­[*b*]-1,5-naphthyridine and acridine (**2a**, IC_50_ *Pf* ^3D7^= 240 nM), highlighting the importance of this moiety to the potency and the significance of the basic nitrogen in potent antimalarial compounds.^30^ The bioisosteric substitution of the pyrrolidine to dimethylamine (**2c** → **2d**) led to no differences in potency or cytotoxicity. However, eliminating only one pyrrolidine ring offered an opportunity to considerably improve the selectivity index by reducing the cytotoxicity, as demonstrated by compounds **2b** and **2k**, which contain only one pyrrolidine ring in their chemical structure and exhibit higher selectivity. Interestingly, when the CF_3_ group is presented at R^2^ (**2g**) with both pyrrolidine rings at R^3^ and R^4^, the potency increases 2-fold with no changes in cytotoxicity compared to its counterpart containing only one pyrrolidine ring (**2f**). Hence, in this case, the presence of two pyrrolidine groups at R^3^ and R^4^ results in a compound 2-fold more selective. In addition, we assessed the influence of the methoxy group at R^5^ (**2c**) in comparison with a chlorine atom (**2j**), revealing no significant differences. However, the absence of the pyrrolidine ring at R^3^ significantly reduces the cytotoxicity of both **2c** and **2j**, leading to impressive selectivity index values of 3000 (**2b**) and 750 (**2k**), as previously stated. A summary of structure–activity relationships highlighting key substitutions and their effects on potency and selectivity is shown in Figure 1A.

**Figure 1:**
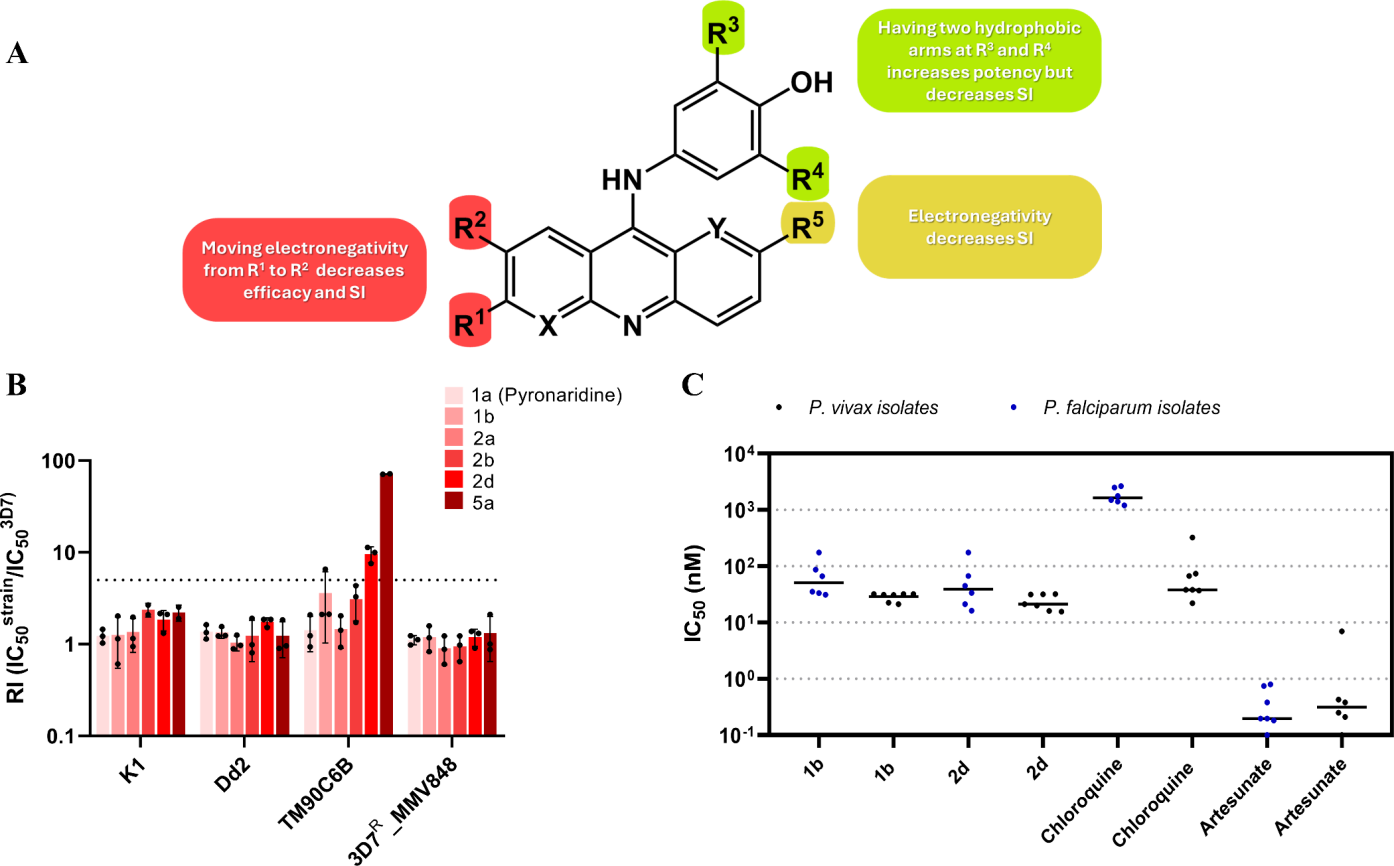
Structure–activity relationships (SAR) and *in vitro* and *ex vivo* antiplasmodial properties of AC-based compounds. (A) Summary of structure–activity relationships (SAR). (B) Resistance indexes of AC-based compounds against a selection of panel of multidrug-resistant *P. falciparum* strains. The resistance indexes (RI) were determined by the ratio of IC_50_ values of the resistant strains Dd2, K1, TM90C6B, and 3D7^R^_MMV848 per the sensitive strain 3D7. These data correspond to three independent experiments, mean ± SD. As a note, cross-resistance is considered when RI > 5, represented by the dashed line. (C) Evaluation of the *ex vivo* activity of compounds **1b** and **2d** against field isolates of *P. vivax* and *P. falciparum* from Porto Velho-RO/Brazil. The antimalarials chloroquine and artemisinin were used as controls.

For the quinacrine derivatives series (Table 2), only a few modifications were performed to enable a better understanding of the influence of the substituent groups directly linked to the acridine scaffold. We also assessed the acridone scaffold derivative **5a**, with the removal of the diethylpentane-1,4-diamine moiety. In line with the findings observed for the pyronaridine derivatives, shifting the chlorine atom from R_1_ to R_2_ (**4a** → **4b**) resulted in a loss of potency while maintaining the cytotoxicity constant. However, in contrast to the observations for pyronaridine derivatives, the presence of the trifluoromethyl group at the R_2_ position led to a reduction in potency (**4c**, IC_50_ *Pf* ^3D7^= 60 nM), contrary to the trend previously observed for the most potent compound, **2g**. Additionally, replacing the methoxy group (**4a**) with a chlorine atom (**4d**) revealed no significant differences. By changing the scaffold to acridone (**5a**), reduced potency against the parasite was observed when compared to pyronaridine (**1a**) and quinacrine (**4a**). In this way, the amino-phenol and the diethylpentane-1,4-diamine moieties prove to be important in achieving more potent and selective compounds.

**Table 2:**
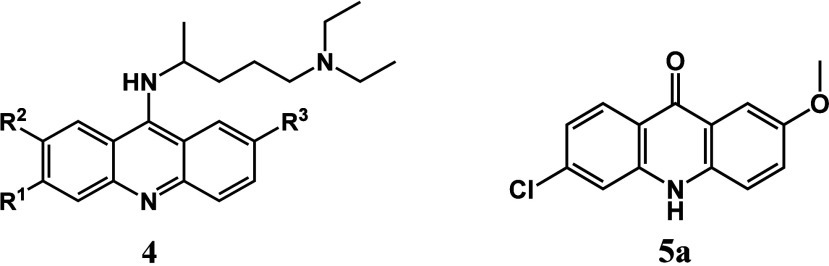
*In Vitro* Antiplasmodial Activity against Chloroquine-Sensitive (3D7) Strain of *P. falciparum*, Cytotoxicity against the HepG2 Cell Line, and Selectivity Index of Quinacrine Derivatives^a^

**compound**	**R** ^**1**^	**R** ^**2**^	**R** ^**3**^	**IC**_**50**_ *Pf* ^**3D7**^ **(nM)**	**CC**_**50**_ **HepG2 (μM)**	**SI**
**4a, quinacrine**	Cl	H	OMe	12 ± 5	4 ± 2	333
**4b**	H	Cl	OMe	50 ± 20	4.1 ± 0.5	82
**4c**	H	CF_3_	OMe	60 ± 10	17 ± 4	283
**4d**	Cl	Cl	Cl	20 ± 10	5 ± 2	250
**5a**				728 ± 6	>6.25	>8
**artesunate**				15 ± 4	n.d.	n.d

a Mean values are represented with their respective standard deviations (n.d. = not determined).

The antiplasmodial activity of three additional and distinct AC-based compounds (**5b**, **6**, and **7**) was assessed (Table S1). These derivatives demonstrated limited inhibitory effects. Specifically, modifications included substitution at position 3 (e.g., morpholine) and position 4 (e.g., nitrile) of the benzo­[*b*]-1,6-naphthyridine (**6**) and 10-oxo-5,10-dihydrobenzo­[*b*]-1,6-naphthyridine (**5b**) cores (IC_50_ *Pf* ^3D7^ > 10 μM), as well as substitution with a morpholine group at position 9 of the acridine core (**7**, IC_50_ *Pf* ^3D7^ = 3.3 μM).

Subsequent assays were then conducted with the aim of characterizing the antimalarial properties of this series. Thus, representative acridine- (e.g., **1a** (pyronaridine), **1b**, **2a**, **2b**, **2d**, **4a** (quinacrine)) and acridone-based compounds (**5a**) were used.

### Inhibitory Activity against Multidrug-Resistant *P. falciparum* Strains

The potency of representative AC-based compounds against sensitive and multidrug-resistant (MDR) *P. falciparum* strains was assessed. This representative resistant-strain panel includes K1 and Dd2 (both resistant to chloroquine, sulfadoxine, pyrimethamine, mefloquine, and cycloguanil),^32^ TM90C6B (resistant to chloroquine, pyrimethamine, and atovaquone),^32^ and 3D7^R^_MMV848 (a 3D7-derived strain resistant to MMV692848, a *Pf*PI4K inhibitor).^33^

The AC-based compounds **1b**, **2a**, **2b**, **2d**, and **5a** demonstrated no significant change in IC_50_s when tested against mutant lines K1, Dd2, and 3D7^R^_MMV848 (RI < 5) (Figure 1B). A previous report indicated that **5a** displayed IC_50_ values of 45 and 65 nM against sensitive (D6) and resistant (Dd2) *P. falciparum* strains, respectively.^34^ These values are ∼10-fold lower than those obtained herein, likely due to differences in the strain origin and genetic background (D6 × 3D7) used.^35^ Despite some potency differences, our results are consistent with earlier findings in showing that **5a** exhibits comparable IC_50_’s between sensitive (3D7) and resistant strains (K1, Dd2, 3D7^R^_MMV848 (RI ∼ 1)). This lack of differential activity suggests the absence of a shared resistance phenotype among the tested mutants, indicating that these derivatives may act via an alternative mechanism or that the mutations do not influence their binding mode.^32^ However, compounds **2d** and **5a** exhibited reduced potency against TM90C6B strain (RI > 5), suggesting cross-resistance with atovaquone, a mitochondrial cytochrome bc1 complex inhibitor. The derivatives **2a**, **2b**, and **2c** did not exhibit reduced potency against TM90C6B strain (RI < 5) (Figure 1B).

### Inhibitory Activity against *P. vivax* and *P. falciparum* Field Isolates

The *ex vivo* activity of representative compounds of subseries 1 (**1b**) and 2 (**2d**) was evaluated against field isolates of *P. vivax* and *P. falciparum* circulating in Porto Velho-RO (Brazilian Amazon). Chloroquine and artemisinin were used as controls. The compounds showed comparable potencies between laboratory culture strains and *P. falciparum* and *P. vivax* isolates. Additionally, compounds **1b** (IC_50_ *^Pf^* = 51 nM and IC_50_ *^Pv^* = 31 nM) and **2d** (IC_50_ *^Pf^* = 39 nM and IC_50_ *^Pv^* = 21 nM) showed comparable inhibitory activities between both isolates (Figure 1C). As expected, field isolates of *P. falciparum* were resistant to chloroquine but sensitive to artesunate (Figure 1C).^36^ Conversely, the *P. vivax* isolates were sensitive to both antimalarial controls used (Figure 1C).

### Speed-of-Action of AC-Based Compounds

To investigate the onset of action of our compounds, we examined their time-dependent effects on parasite growth. Although rapid parasite clearance is an important attribute for agents targeting the asexual blood stages,^37^ slower-acting antimalarials also have clear clinical value, especially within combination therapies where they contribute to sustained efficacy. Thus, characterizing the speed of action provides insight into how each compound might be positioned within future treatment strategies. A modified version of the *in vitro* speed-of-action methodology^38^ was employed to differentiate between fast- and slow-acting inhibitors.^39^ The incubation of fast-acting antimalarials for 24 h, as artesunate and chloroquine, yielded IC_50_s like the standard 72 h assay, with IC_50_^24h^ /IC_50_^72h^ ratios ∼ 1. In contrast, slow-acting controls (atovaquone and pyrimethamine) exhibited IC_50_^24h^ /IC_50_^72h^ ratios greater than 1 (Figure 2A). The subseries controls pyronaridine (**1a**) and quinacrine (**4a**), as well as the tested compounds **1b**, **2a**, and **2d**, showed IC_50_^24h^ /IC_50_^72h^ shifts ∼ 1, like chloroquine and artesunate, consistent with a fast-acting mechanism. On the other hand, compound **5a** demonstrated an IC_50_^24h^ /IC_50_^72h^ ratio >1, suggesting a slow action profile (Figure 2A). To confirm the speed-of-action, the morphological development of the parasite after 24 h of inhibitor exposure was verified (Figure 2B). The parasites in the negative control (without compound) developed according to the expected timeline, progressing from ring to trophozoite in the first 24 h and later from trophozoite to schizont, with new rings observed in 48 h, indicating one complete maturation cycle of the parasite. Over the incubation time of 72 h, trophozoites were observed in the control without compounds (Figure 2B). Artesunate (fast-acting control) caused parasite death within the first 24 h, as observed by the appearance of pyknotic nuclei, the irreversible chromatin condensation of a necrotic or apoptotic cell (Figure 2B). By contrast, atovaquone (slow-acting inhibitor) allowed the parasite to develop from ring to trophozoite in 24 h, delaying the development past this point, with ring-stage parasites still observed in 72 h. The subseries controls pyronaridine (**1a**) and quinacrine (**4a**), and the tested compounds **1b**, **2a**, and **2d** caused parasite death within the first 24 h of incubation, comparable to artesunate. At this time point, we verified the appearance of several pyknotic nuclei. For compound **5a**, the morphological analysis indicated that the parasite development stalled in the ring stage in 24 and 48 h, making it possible to observe young and mature trophozoite stages in 72 h. These findings confirm that the subseries controls, pyronaridine (**1a**) and quinacrine (**4a**), as well as the tested compounds **1b**, **2a**, and **2d**, act as fast-acting inhibitors, while compound **5a** shows a nonfast-acting profile.

**Figure 2:**
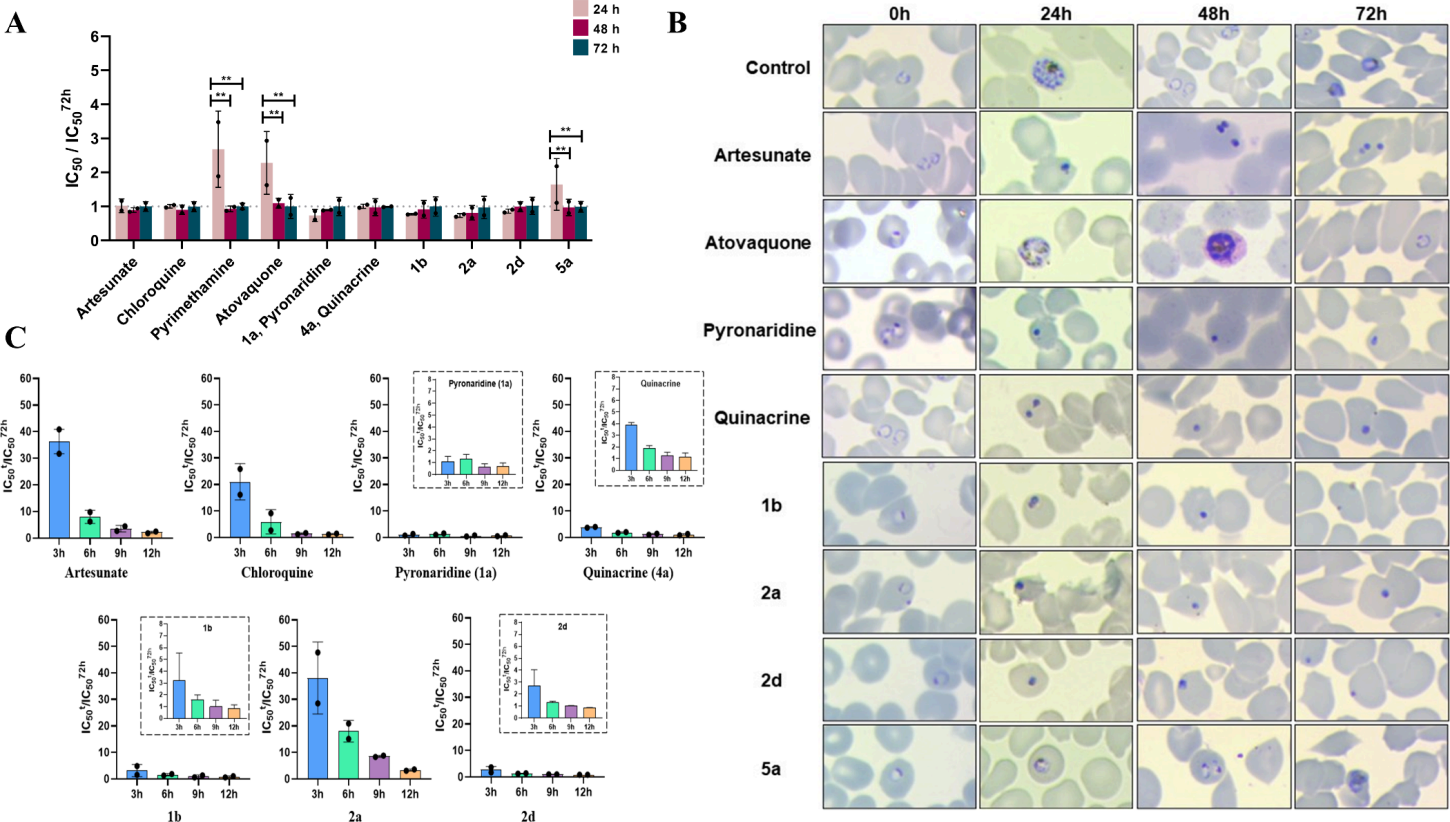
Speed-of-action of AC-based compounds. (A) Speed-of-action assessment of **1b**, **2a**, **2d**, and **5a**. Pyronaridine (**1a**) and quinacrine (**4a**) were used as control of the AC-based subseries. IC_50_ values were determined at 72 h, for parasites (3D7 strain) incubated for 24, 48, and 72 h with compounds. Artesunate and chloroquine were used as a positive control for fast-acting inhibition, and pyrimethamine and atovaquone were used as slow-acting inhibition controls. The results were normalized to the assessed IC_50_ value at 72 h. Ratios IC_50_^24h^ /IC_50_^72h^ ≤ 1 distinguish fast-acting compounds from nonfast-acting ones, represented by the dashed line. These data correspond to two independent experiments, mean IC_50_ ± SD. Statistical analysis was conducted by using ANOVA (** *p* < 0.01; a *p* value <0.05 indicates a significant difference within samples) (B) Morphological development of *P. falciparum* (3D7 strain) over 24 h of incubation with the AC-based compounds. The *P. falciparum* culture at 0.5% parasitemia was incubated for 24 h at 10× the IC_50_ with the fast- and slow-acting controls (artesunate and atovaquone, respectively), as well as the subseries controls (pyronaridine (**1a**) and quinacrine (**4a**)), and tested compounds **1b**, **2a**, **2d**, and **5a**. Images were taken at 24, 48, and 72 h. (C) Fast-acting assessment of artesunate, chloroquine, pyronaridine (**1a**), quinacrine (**4a**), **1b**, **2a**, and **2d** after short-term exposure. IC_50_ ratios obtained after 3, 6, 9, and 12 h of exposure followed by washing and incubation until 72 h. Graphs represent the ratio between each time point IC_50_^t^ and 72h IC_50_. Data are presented as mean ± SD of two independent experiments.

To verify how fast the inhibitors were, the compounds were incubated with parasites for 3, 6, 9, and 12 h. Next, the samples underwent washing and were further incubated under inhibitor-free conditions until 72 h, and then the IC_50_^t^/IC_50_^72h^ ratios for each time point were calculated. The controls, artesunate and chloroquine, demonstrated decreased inhibitory activity within the initial 3 h of incubation, with IC_50_ ratios >25-fold compared to their respective 72 h IC_50_ values (Figure 2C). Extension of the incubation to 6 h resulted in 5- to 8-fold reductions in IC_50_ ratios. Expected levels of antiplasmodial activity were achieved from 9 h onward (IC_50_ ratios <2). Among the subseries controls, pyronaridine (**1a**) exhibited IC_50_ ratios ∼ 1 across all time points, indicating that a 3 h incubation period is sufficient for its full inhibitory effect (Figure 2C). Quinacrine (**4a**) showed only 4-fold reduction in inhibitory activity following 3 h of incubation when compared to the 72 h IC_50_ but reached IC_50_ ratios <2 from 6 to 12 h. With respect to the tested compounds, **2a** displayed behavior comparable to artesunate and chloroquine, requiring more than 9 h of incubation to reach maximal inhibitory activity (Figure 2C). Compounds **1b** and **2d** exhibited ∼3-fold reductions in inhibitory activity after 3 h of incubation relative to the 72 h IC_50_ and showed IC_50_ ratios <2 during the 6 to 12 h interval. These results confirm that the compounds are fast-acting inhibitors, exhibiting maximal activity in less than 12 h of incubation.

### Stage-Specific Inhibitory Activity of AC-Based Compounds

To investigate the stage-specific effects of the representative compounds **2d** and **5a**, as well as chloroquine and pyronaridine (**1a**) (controls), highly synchronized *P. falciparum* cultures (ring stages) were prepared. The compounds were incubated with parasites at the ring (0–16 h), trophozoite (16–32 h), and schizont (32–48 h) stages. Parasitemia was assessed at 60 h using the SYBR Green assay. For comparison purposes, IC_50_ values were also determined under continuous exposure throughout the full 72 h erythrocytic cycle. Chloroquine displayed potent inhibitory activity against ring stages, moderate activity against trophozoites, and no measurable effect on schizonts (Figure 3A). Similarly, pyronaridine (**1a**) (Figure 3B) and **2d** (Figure 3C) exhibited a stage-dependent activity profile similar to that of chloroquine, with a pronounced inhibitory effect on the ring and trophozoite stages. In contrast, compound **5a** did not show detectable inhibitory activity against any stage of parasite development (IC_50_s > 10 μM), suggesting that its antiplasmodial effect requires prolonged exposure, as observed in the 72 h assay (IC_50_ ∼ 500 nM) (Figure 3D).

**Figure 3:**
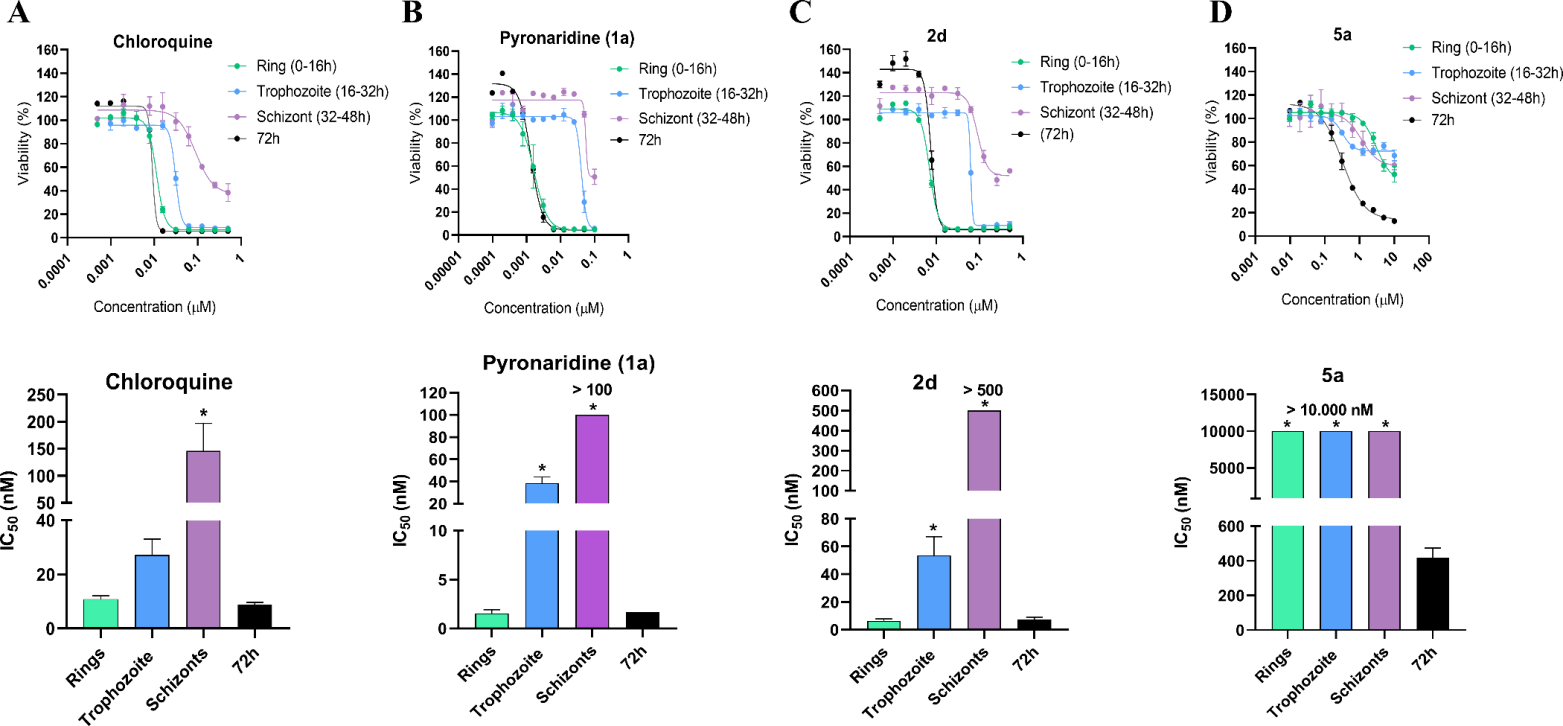
Stage-specific activity of AC-based derivatives. (A) Chloroquine; (B) pyronaridine (**1a**); (C) **2d**; and (D) **5a**. The compounds were tested against ring (0–16 h), trophozoite (16–32 h), and schizont (32–48 h) stages of *P. falciparum* using highly synchronized cultures. Parasitemia was measured at 60 h by the SYBR Green assay. Data are presented as mean ± SD of two independent experiments. Statistical analysis was performed using one-way ANOVA followed by the Bonferroni post hoc test (*p < 0.05).

### Combination Studies of AC-Based Compounds with Proguanil

The data obtained from the cross-resistance, speed-of-action, and stage-specific assays for compound **5a** suggest unique characteristics within the tested series of this study. These findings led us to hypothesize that compound **5a** acts distinctively from the other tested compounds. Furthermore, its resistance profile against the TM90C6B strain suggests a potential similarity in antiplasmodial action to that of atovaquone. Given that the combination of atovaquone and proguanil (Malarone) is synergistic against *P. falciparum*,^40^ the combination of the subseries controls pyronaridine (**1a**) and quinacrine (**4a**) and **1b**, **2a**, **2d**, and **5a** with proguanil was evaluated. As expected, atovaquone showed synergistic behavior in combination with proguanil (Figure 4A). The same behavior was observed for compound **5a** combined with proguanil against *P. falciparum* (Figure 4B). These findings indicated that compound **5a** may share the same mode of action as atovaquone (e.g., inhibition of the cytochrome bc1 complex). On the other hand, both pyronaridine (**1a**) and **2d** in combination with proguanil showed antagonistic behavior (Figure 4C,D, respectively). Compounds **1b**, **2a**, and quinacrine (**4a**) also showed antagonistic interactions with proguanil (Figure S2).

**Figure 4:**
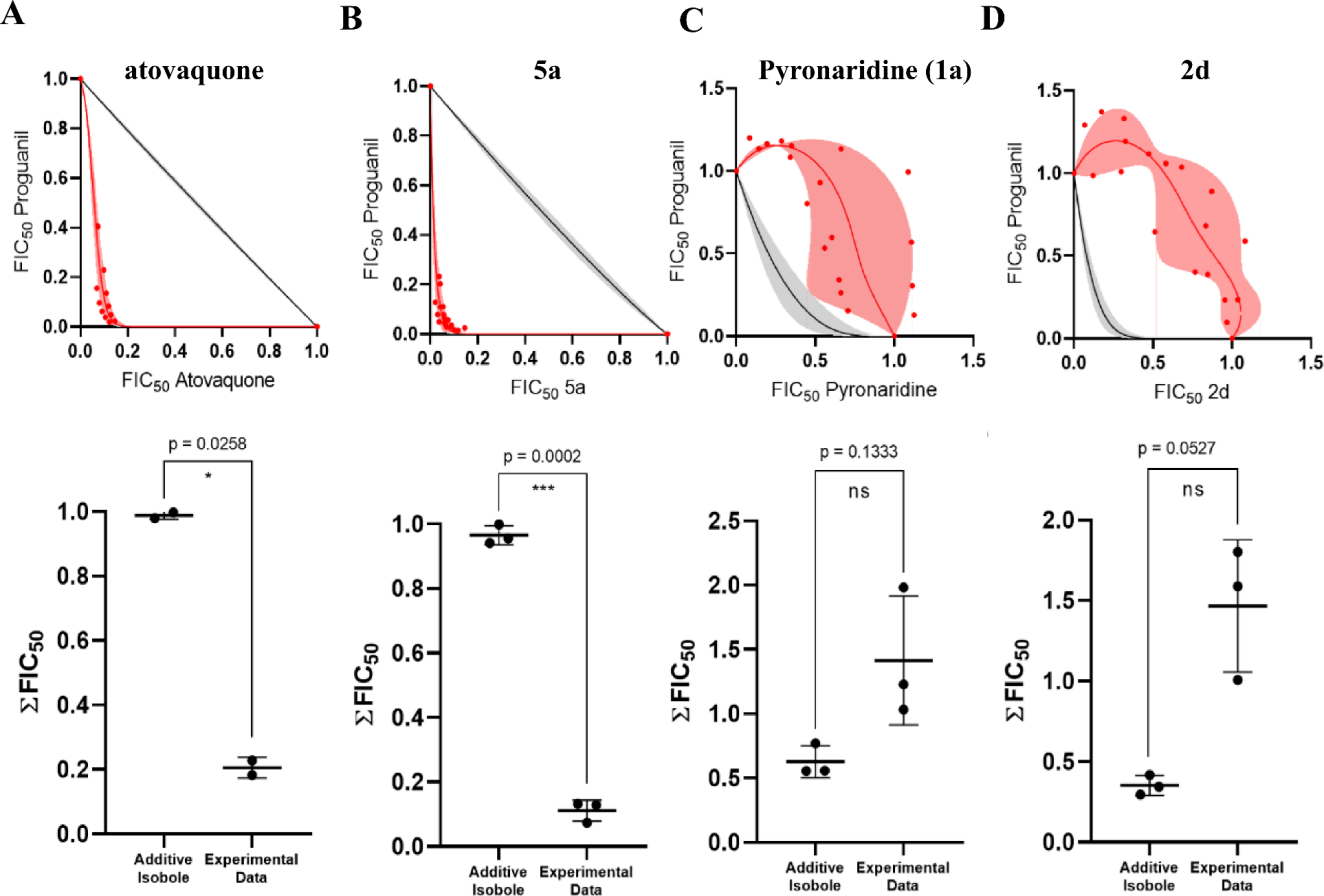
Combination investigation with proguanil. Upper panels: Isobolograms of association of atovaquone (A), **5a** (B), pyronaridine (**1a**) (C), and **2d** (D) with proguanil. Experimental data are represented by the red region and red dots, while the black line and gray region indicate the additivity curve. Bottom panels: Statistical analysis for the combination of atovaquone, **5a**, pyronaridine (**1a**), and **2d** with proguanil. The data represent the ∑FIC50 values from three independent experiments. Statistical analysis was conducted by using Student’s paired *t* test (*p* value <0.027 indicates a statistical distinction between the experimental findings and the additivity isobole).

### Cellular Localization of AC-Based Compounds by Confocal Microscopy

The intracellular distribution of compound **2d**, **5a**, and pyronaridine (**1a**) (subseries control) in *P. falciparum*-infected erythrocytes was analyzed by fluorescence microscopy. The compounds were tested at 10 μM. Confocal microscopy images and fluorescence measurements of infected erythrocytes revealed the selective uptake of **2d** by the parasites, particularly in two small spheres located near the digestive vacuole (DV), identified by the existence of the dark hemozoin crystals (Figure 5A,B). Furthermore, faint and diffuse cytoplasmic fluorescence was noted in addition to the punctate labeling. No labeling was observed in uninfected red blood cells, indicating the specificity of accumulation within *P. falciparum* parasites. The subseries control, pyronaridine (**1a**), demonstrated intrinsic fluorescence intensities significantly weaker compared to that of **2d** (Figure 5B). Nonetheless, a detectable distinction in fluorescence intensity was measured between the parasite DV region and a corresponding area in uninfected erythrocytes.

**Figure 5:**
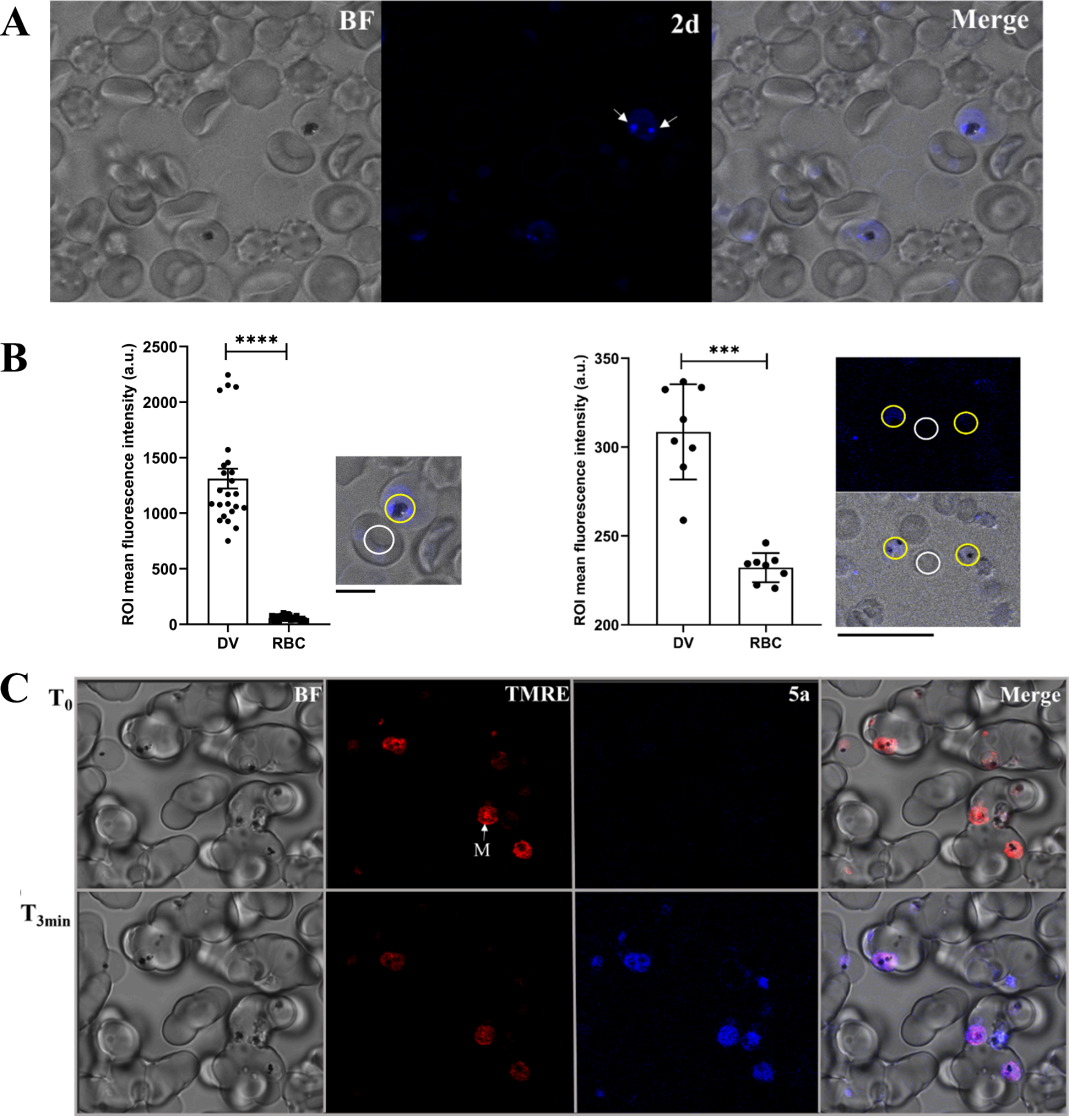
Intracellular localization of compounds **2d** and **5a** in *P. falciparum*-infected erythrocytes. (A) Confocal microscopy images showing the bright field (BF) and the distribution of compound **2d** (blue) at 10 μM in *P. falciparum*-infected erythrocytes. White arrows indicate patterns of accumulation close to the hemozoin and hence the digestive vacuole (DV). (B) Fluorescence measurements of infected erythrocytes in the presence of compounds **2d** and pyronaridine (**1a**) by confocal microscopy. Mean fluorescence intensity was measured within regions of interest (ROIs) surrounding the DV of *Plasmodium*-infected erythrocytes (yellow circles) and compared with ROIs of equivalent size selected in uninfected red blood cells (RBC) (white circles). ****p ≤ 0.0005, Student’s *t* test; a.u., arbitrary units. Scale bars of 5 μm for **2d** and **25** μm for pyronaridine (**1a**). (C) Detection of **5a** on Δ_Ψm_ from *P. falciparum* parasites in live cell confocal microscopy images. Bright-field (BF) and 100 nM TMRE fluorescence (red) images of infected erythrocytes in the initial time fluorescence (T0) (upper panel) and 3 min (lower panel) after the addition of 10 μM of **5a** (blue). ‘M’ indicates the parasite mitochondrion.

To provide further evidence that compound **5a** might be targeting the parasite’s mitochondria, we monitored the effect of this compound on the mitochondrial membrane potential (Δ_Ψm_) by measuring the fluorescence changes in cells loaded with tetramethylrhodamine ethyl ester (TMRE) in real time. TMRE is a cationic red-orange, fluorescent dye that reversibly accumulates inside energized membranes. Upon addition of TMRE to *P. falciparum*-infected erythrocytes, a fluorescent signal (red) was observed in specific subcellular locations within the cytosol (except for the food vacuole), corresponding to plasma and mitochondrial membrane potential (Figure 5C, upper panel). When adding the autofluorescent compound **5a** (blue), a simultaneous reduction in the red fluorescence of TMRE was observed as **5a** accumulates inside the parasite, indicating that it collapses membrane potential-dependent accumulation of TMRE in *P. falciparum*-infected erythrocytes (Figure 5C, lower panel). This data is further evidence that **5a** may be promoting parasite death through a mechanism associated with the mitochondria in the parasite. Therefore, the microscopy analysis indicated that the localization of pyronaridine derivative **2d** is distinct from that observed for compound **5a**.

### Inhibition of Cytochrome bc1 Complex and Hemozoin Formation by AC-Based Compounds

To evaluate our hypothesis that compound **5a** inhibits cytochrome bc1 complex, an enzymatic assay was conducted to measure cytochrome bc1 complex decyl ubiquinol-cytochrome c oxidoreductase activity. Mitochondrial fractions were extracted from *P. falciparum* 3D7 parasites, and the assay was performed using compounds **5a** and pyronaridine (**1a**) in parallel with negative (DMSO) and positive (atovaquone) controls. Atovaquone showed an IC_50_ of 16 nM against the *P. falciparum* cytochrome bc1 activity, confirming the high potency described previously.^41^ Pyronaridine showed no inhibition of the cytochrome bc1 complex (IC_50_ > 200 μM) (Figure 6A). On the other hand, compound **5a** showed an IC_50_ of 2 μM against the *P. falciparum* cytochrome bc1 (Figure 6A). These data indicated that **5a** inhibits the parasite bc1 complex.

**Figure 6:**
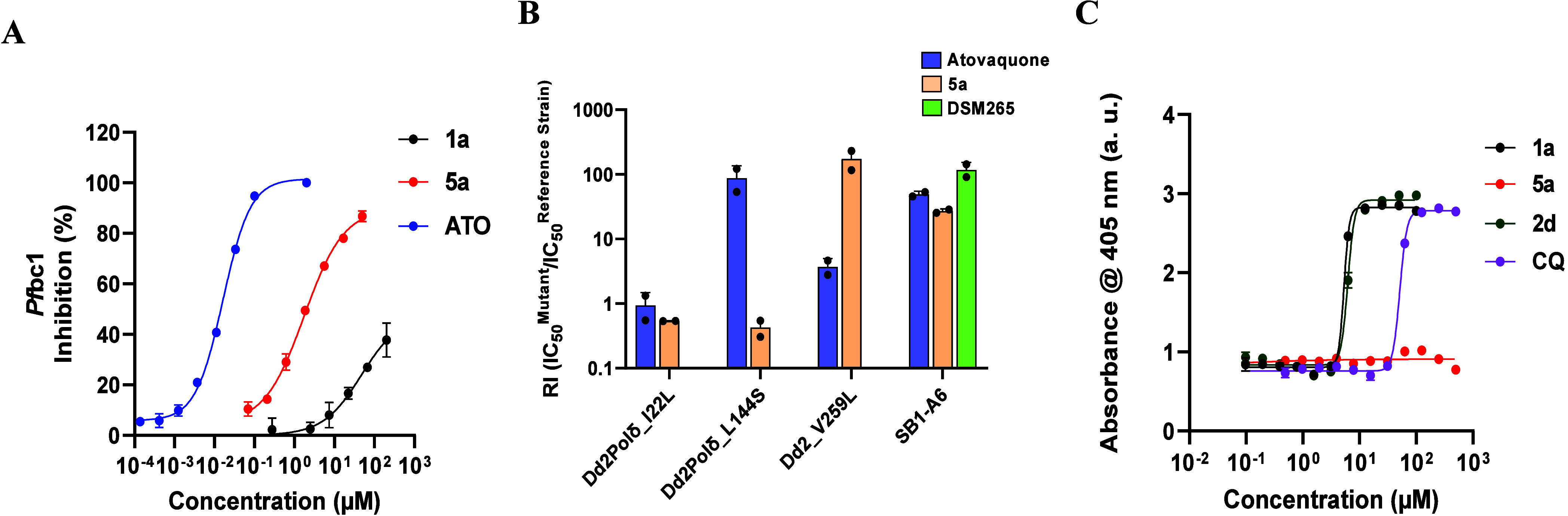
(A) Inhibition curves of the *P. falciparum* cytochrome bc1 complex by pyronaridine (**1a**) (black) (IC_50_ > 200 μM), compound **5a** (red) (IC_50_ = 2.01 ± 0.02 μM), and atovaquone (blue) (IC_50_ = 0.016 ± 0.002 μM). Data are representative of three independent experiments. (B) Resistance index (RI = IC_50_^Mutant^/IC_50_^Reference Strain^ for **5a** and atovaquone against Dd2_V259L (mutation in Qo), Dd2Polδ _I22L (mutation in Qi), Dd2 Polδ _L144S (mutation in Qo), and SB1-A6 (*Pf*DHODH CNV (2-fold) + C276F mutation), and for DSM265 in SB1-A6. (C) β-Hematin inhibition by chloroquine (green) (IC_50_ = 49 ± 3 μM), pyronaridine (**1a**) (black) (IC_50_ = 5 ± 1 μM), compound **2d** (purple) (IC_50_ = 5 ± 2 μM), and compound **5a** (red) (IC_50_ > 1000 μM). Data are representative of three independent experiments. Absorbance units (a.u.) were recorded at 405 nm.

To further probe target engagement, compound **5a** was evaluated against four *P. falciparum* mutant strains: Dd2_V259L harboring the V259L mutation in the Qo site of Cytochrome *b*;^42^ Dd2Polδ_I22L and Dd2Polδ_L144S, mutants named after substitutions in the Qi and Qo subsites of the cytochrome bc1 complex, respectively;^43^ and SB1-A6, a strain carrying both a copy number variation (∼2-fold) and a C276F mutation in the *P. falciparum* dihydroorotate dehydrogenase (*Pf*DHODH) gene.^44^ The resistance indexes (RI) were calculated as the ratio between the IC_50_ values of the mutant strains and the reference strain. Specifically, IC_50_ values for Dd2Polδ_I22L and Dd2Polδ_L144S were compared to those of Dd2Polδ, SB1-A6 to 3D7, and Dd2_V259L to Dd2. Atovaquone and DSM265 were used as reference controls. Compound **5a** exhibited a high level of resistance in both the Dd2_V259L and SB1-A6 strains (Figure 6B). A similar resistance profile was observed for atovaquone in Dd2_V259L and for both atovaquone and DSM265 in SB1-A6, indicating cross-resistance in this genetic background. However, compound **5a** showed no cross-resistance against the Dd2Polδ_L144S (Qo) and Dd2Polδ_I22L (Qi) strains, maintaining its potency in these mutant lines. In contrast, atovaquone showed no cross-resistance in Dd2Polδ_I22L but exhibited high resistance in Dd2Polδ_L144S, consistent with its known interaction with the Qo subsite of Cytochrome *b*.^43^

To assess whether the tested compounds impact hemozoin formation, a β-hematin inhibition assay was performed with compounds **2d** and **5a**, as well as pyronaridine (**1a**) and chloroquine (used as positive controls) (Figure 6C). Pyronaridine (**1a**) (IC_50_ = 5 μM) and compound **2d** (IC_50_ = 5 μM) demonstrated greater inhibition of β-hematin formation compared to chloroquine (IC_50_ = 49 μM), while compound **5a** showed minimal inhibitory activity under the same assay conditions (Figure 6C). In conjunction with the observed localization of **2d** near the parasite’s food vacuole (Figure 5A), these findings corroborate the proposed mechanism of action for compound **2d**, indicating that its antiplasmodial activity is achieved through interference with the hemozoin formation pathway. Additionally, the potent inhibitory effect of pyronaridine agrees with previous findings indicating a similar mechanism for acridine derivatives.^16,17^

### Resistance Selections for AC-Based Compounds

Aiming to understand the mode of action of compound **5a**, selection of resistant parasites was performed using Dd2-Polδ parasites in three flasks with an initial inoculum of 1 × 10^9^ parasites per flask under a selection pressure of 3 × IC_90_. The same protocol was applied to pyronaridine (**1a**) (control). Genetically engineered Dd2-Polδ parasites have D308A and E310A mutations in the delta subunit of DNA polymerase (PF3D7_1017000), which increases the propensity for the parasites to acquire mutations.^45^ The parasite clearance was observed on days 4 and 7 for pyronaridine (**1a**) and compound **5a**, respectively, then the inhibitors selection pressure was removed on day 17, and cultures were monitored for recrudescence for 60 days (Figure 7A,B). No recrudescence was observed for pyronaridine (**1a**) during the 60 days of experiment (Figure 7A), whereas selection with compound **5a** yielded recrudescent parasites in all 3 flasks on day 21 of selection (Figure 7B). Selected parasites from each flask were phenotyped and showed IC_50_ shifts from 8- to 25-fold compared to the parental strain (Figure 7C).

**Figure 7:**
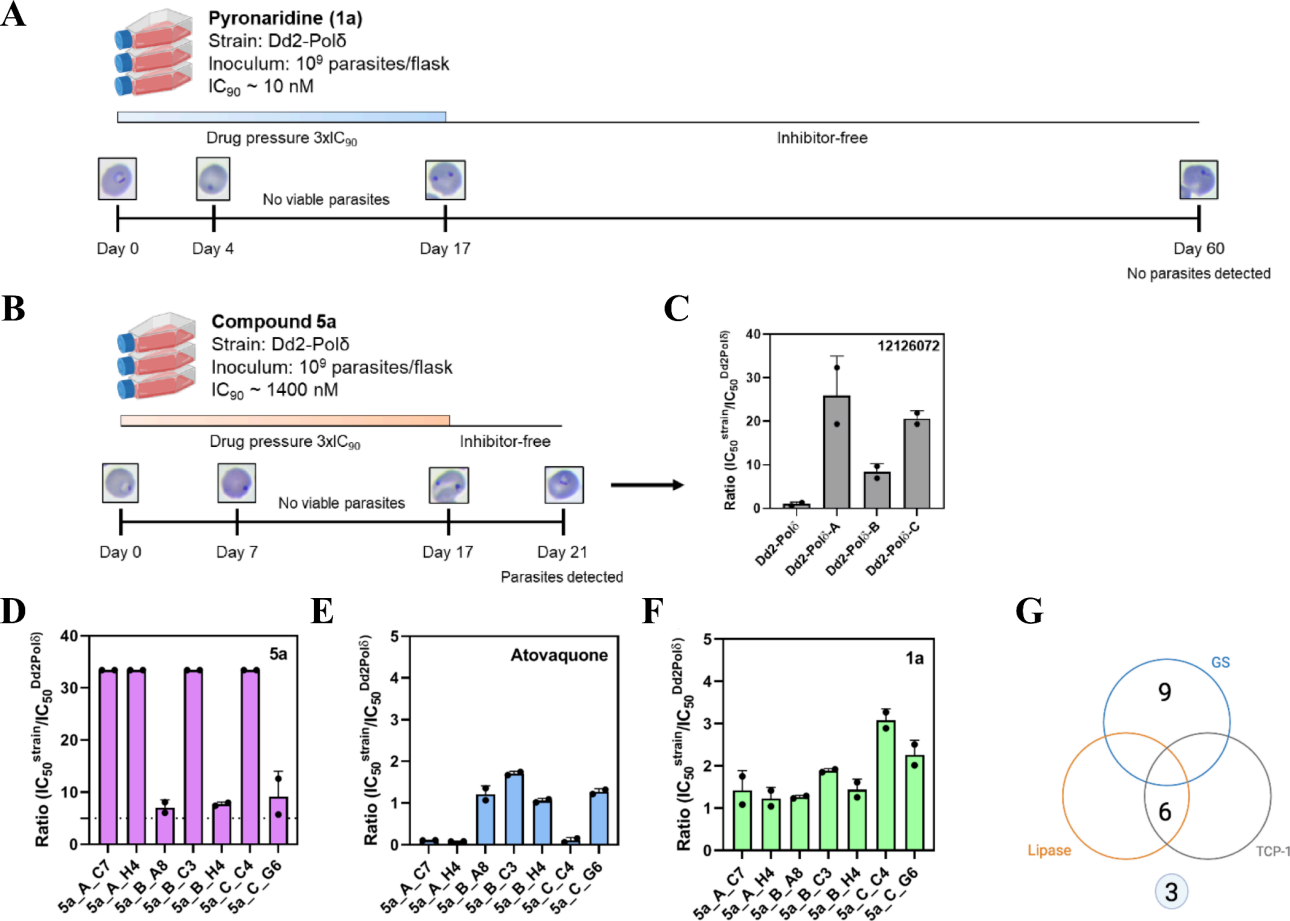
Generation of parasite resistance to pyronaridine (**1a**) and compound **5a**, inhibitory potency on isolated clonal parasites, and number of resistant *P. falciparum* clones harboring mutated genes. (A) Selection scheme showing inability to evolve resistance to pyronaridine (**1a**) with Dd2-Polδ (B) but successful isolation of resistance with compound **5a** in 3 independent flasks. (C) Ratios between IC_50_ values of resistant line to **5a** from each flask (Dd2-Polδ A-C) and IC_50_ values of Dd2-Polδ parental line. (D–F) IC_50_ shifts of clonal parasites compared to the parental Dd2-Polδ line. (D) compound **5a**, (E) atovaquone, and (F) pyronaridine (**1a**). Data shown is mean ± SD. (G) Venn diagram showing the number of clones resistant to **5a** sharing mutations in *P. falciparum* glutathione synthetase (GS), T-complex protein 1 (TCP-1), and lipase genes.

Limiting dilution was performed with cultures from all flasks to obtain six clonal parasites from each flask. Compound **5a** elicited varying levels of resistance among seven selected clones, which were sent for whole-genome sequencing (WGS). Clones from flask A (6a_A_C7 and 6a_A_H4) were highly resistant to **5a**, exhibiting a 30-fold reduction in potency compared to Dd2-Polδ parasites. Clones isolated from flasks B and C showed different levels of resistance: two clones (6a_B_C3 and 6a_C_C4) were highly resistant, exhibiting a 30-fold shift in IC_50_ compared to the parental parasites, while three others (6a_B_A8, 6a_B_H4, and 6a_C_G6) exhibited IC_50_ values for compound **5a** that were 8-fold higher than the parental strain (Figure 7D). Furthermore, the effects of atovaquone (Figure 7E) and pyronaridine (**1a**) (Figure 7F) were investigated in these clonal parasites, and no cross-resistance to either antimalarial was observed.

The WGS analysis of the seven selected clones revealed the acquisition of three mutations across three genes relative to the parental strain Dd2-Polδ. Clones from Flask B (5a_B_C3, 5a_B_A8, and 5a_B_H4) exhibited a M521I mutation in the T-complex protein 1 (TCP-1) gene and a I1264N mutation in the putative lipase gene, whereas clones from Flask A (5a_A_C7 and 5a_A_H4) and Flask C (5a_B_C3) displayed a M42I mutation in the glutathione synthetase (GS) gene (Tables S2 and S3). Given that these clones did not share common genetic alterations, we conducted direct Sanger sequencing to determine whether the remaining 11 clones, which were not submitted to WGS, were resistant to **5a** carried mutations in the candidate genes previously identified (Tables S2 and S3). The combined analysis of WGS and Sanger sequencing data revealed that 50% of the clones (9/18) exhibited the M42I mutation in the GS gene, 33% (6/18) carried the M521I mutation in the TCP-1 gene and I1264N mutation in the lipase gene, and 17% (3/18) had no mutations in these genes (Figure 7G). These findings suggest that compound **5a** may independently be selected for two distinct pathways.

### *In Silico* and *In Vitro* ADME Properties of AC-Based Compounds

We generated multiple classification models using our proprietary Assay Central software.^46^ Models were generated with extended-connectivity fingerprints (ECFP6) and the algorithms used produces a probability-like score and an applicability score for individual chemical compounds. Models are built on training data derived from various public data sets, including from databases such as ChEMBL^47^ and from the literature. Typically, a threshold of 0.5 is used to determine if a compound is predicted to be active, but higher scores suggest a higher likelihood of activity. Applicability scores consider both the model overlap and the individual bias and precision of the overlapping fingerprints.^48^ There is not a defined threshold for an acceptable applicability score, but it is ideal to have a higher score for confidence in the prediction (1 max). The consensus scores are based on majority rule classification (agreement for ≥4 algorithms, when equal to 4, a compound is predicted as active). The threshold for activity is shown for each model. ADMET model predictions were conducted for compounds **2d** and **5a**. Compound **2c**, an equipotent and close analogue of **2d,** was included in the analysis because it could serve as a surrogate compound for *in vivo* studies.

These models predicted low acute oral toxicity for all compounds (only predicted toxicity at ≥2000 mg/kg model for **2d**/**2c**) as well as no predicted AMES mutagenicity nor P-glycoprotein or CYP inhibition. All compounds are predicted to have high bioavailability and reasonable human clearance with high intestinal absorption. All compounds are also predicted to pass through the blood–brain barrier (BBB). hERG toxicity is predicted for both **2c** and **2d** but not for **5a**. The consensus score and applicability domain scores are all shown in Tables S4–S6.

In addition to the predicted ADME properties, we experimentally assessed physicochemical (e.g., solubility) and pharmacokinetic (e.g., metabolic stability, plasma protein binding, stability in human plasma, and permeability) properties of compounds **2c**, **2d**, and **5a** (Table S7). For compound **2d**, solubility was high, and mouse and human liver microsomes showed good metabolic stability. Mouse and human plasma protein binding was high, while *in vitro* stability in plasma was unstable. The Caco-2 efflux ratio was very high, suggesting likely P-gp involvement. For compound **2c**, solubility was low, while mouse and human metabolic stability was good. Mouse and human plasma protein binding was high, while half-life indicated it was unstable. The Caco-2 efflux ratio was also very high, suggesting likely P-gp involvement. In the cases for **2c** and **2d**, some of the predictions did not correspond with the *in vitro* data, which could be because they are outside the applicability domain of the model. For compound **5a**, solubility was very low, and mouse and human liver microsomal stability showed that the compound was unstable. In contrast, the compound had high protein binding and was stable in mouse and human plasma proteins. The efflux ratio was low, indicating no effect of efflux transporters (Table S7).

### *In Vivo* Activity of AC-Based Compounds

To determine whether derivative **2d** could function similarly to pyronaridine (**1a**) in a malaria murine model, we evaluated their *in vivo* antimalarial activity against *P. berghei* (NK65 strain). The *in vivo* activity of compound **5a** was not assessed because the compound showed poor *in vitro* physicochemical and pharmacokinetic properties. Compound **2d** was administered at 50 mg/kg/day for three consecutive days postinfection, and parasitemia was monitored on days 5, 8, and 11. Chloroquine (20 mg/kg/day) served as a positive control. Both pyronaridine (**1a**) and compound **2d** achieved a 100% reduction in parasitemia at all evaluated assessment days comparable to chloroquine (Figure 8A,B, upper panels). In addition, survival analysis revealed that mice treated with pyronaridine (**1a**) and **2d** showed markedly improved survival compared with the untreated control group (Figure 8A,B, bottom panels). The survival of the **2d**-treated group was comparable to those of the chloroquine-treated animals (Figure 8A,B, bottom panels). Compound **1b**, an equipotent and close analogue of **1a**, also showed significant *in vivo* antimalarial activity (Figure S3). Collectively, these results demonstrate that **2d** is well-tolerated and shows substantial antimalarial activity *in vivo*, providing protection in the murine malaria model.

**Figure 8:**
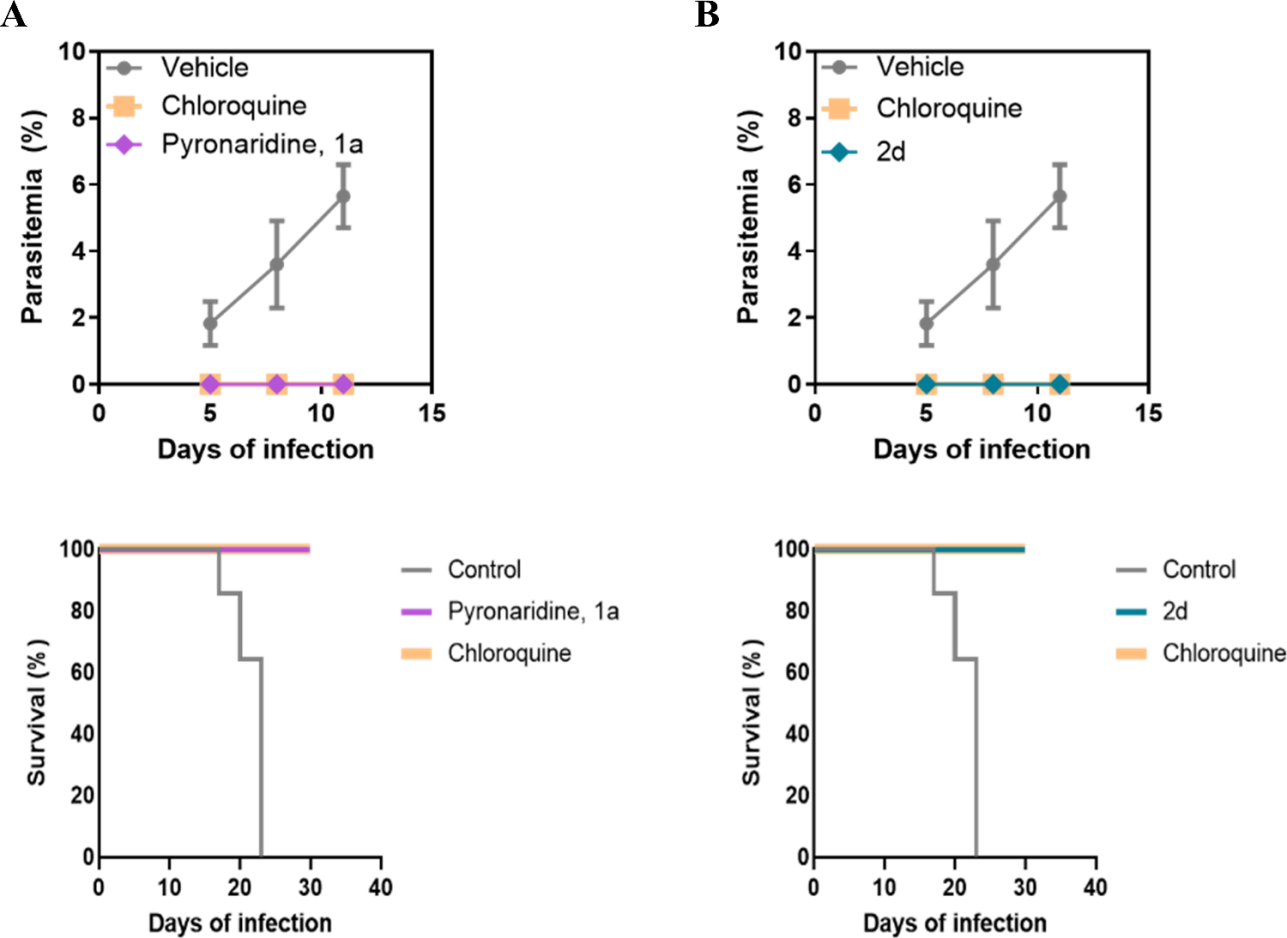
*In vivo* antimalarial activity assessment and survival analysis in mice infected with *P. berghei* **NK65**. Upper panels: Parasitemia reduction following treatment with pyronaridine **1a** (A) and compound **2d** (B) at 50 mg/kg/day for three consecutive days postinfection is shown relative to the untreated control group. Chloroquine (20 mg/kg/day) was included as a reference antimalarial. Bottom panels: Survival analysis of mice treated (PO, 50 mg/kg/day) with compound pyronaridine **1a** (A) and **2d** (B) and standard antimalarial chloroquine evaluated during the experiment (30 days) (*n* = 3–4).

## Discussion

Considering the proliferation of drug-resistant *Plasmodium* parasites, malaria continues to pose a significant challenge to global health.^5,49^ This study explored two series of acridine/acridone compounds: pyronaridine derivatives (**1a–3b**) and quinacrine derivatives (**4a–5a**). Considering that pyronaridine–artesunate (Pyramax) is an approved artemisinin-based combination therapy (ACT) and that cytotoxicity is not a primary clinical limitation for either pyronaridine or quinacrine, we have investigated the critical influence of specific substituent groups and molecular scaffolds (acridine and acridone) on the antiplasmodial potency and cytotoxic effects on HepG2 cells (Figure 1A). HepG2 cells are a well-established and widely used human hepatic model for assessing early toxicity in drug discovery.^50^ Additionally, we have evaluated these compounds against resistant *P. falciparum* strains.^51^ We did not observe notable changes in IC_50_’s of pyronaridine (**1a**) and AC-based compounds evaluated (**1b**, **2a**, **2b**, **2d**, and **5a**) in the Dd2 and K1 strains (Figure 1A), which agrees with earlier findings.^52,53^ Furthermore, AC-based compounds did not show cross-resistance to MMV848, indicating that they do not target *Pf*PI4K, an attractive molecular target for antimalarial discovery.^54^ Nevertheless, we observed that **2d** and **5a** were 7- and 70-fold less potent, respectively, against the TM90C6B strain (Figure 1A), which is resistant to atovaquone (cytochrome bc1 complex inhibitor). Cross-resistance between acridone derivatives and atovaquone has been previously reported. Kancharla et al.^55^ discovered new acridone derivatives with improved efficacy. However, these acridones exhibited cross-resistance to atovaquone. Dodean et al.^56^ conducted a lead-optimization campaign that yielded candidates with enhanced potency and reduced cross-resistance to atovaquone. Nevertheless, these optimized acridone derivatives retained some degree of cross-resistance, consistent with the reduced potency observed for compounds **2d** and **5a** against the TM90C6B strain in our study. Suswam et al.^57^ have reported that the derivatives of acridine, WR243251 (dihydroacridinedione), showed cross-resistance (4- to 9-fold) in *P. falciparum* strains that exhibited a significant increase in resistance (8700- to 23,000-fold) to atovaquone.

Reduced *ex vivo* susceptibility for pyronaridine in African isolates from imported malaria cases^58^ and in *P. vivax* clinical isolates from the China-Myanmar^59^ were reported. Our findings revealed potent inhibitory activities on the *P. falciparum* and *P. vivax* Brazilian field isolates (Figure 1B), indicating that the derivatives are active against circulating strains of the parasites. Notably, even using the hypermutable Dd2-Polδ parasites,^45^ we failed to generate resistance to pyronaridine after selection for 60 days (Figure 7A), reinforcing its low propensity for drug resistance.

The speed-of-action assay showed that pyronaridine (**1a**), pyronaridine derivatives **1b**, **2a**, and **2d**, and quinacrine (**4a**) act as fast-killers of *P. falciparum* parasites, while the acridone-based compound **5a** showed a slow-acting inhibition, like atovaquone (Figure 2). Resistance to atovaquone is a concern and is associated with mutations in the parasite’s Cytochrome *b* gene.^60^ Nevertheless, the synergistic combination of atovaquone and proguanil represents one of the most useful antimalarial medications. Given this, we conducted drug combination studies between AC-based compounds with proguanil. While pyronaridine (**1a**) (Figure 4C) and **2d** (Figure 4D) presented an antagonist association with proguanil, compound **5a** (Figure 4B) revealed significant synergy, comparable to the outcomes of atovaquone (Figure 4A).

The knowledge about the mode of action of acridine derivatives is limited, although some findings suggest that AC derivatives may target the inhibition of hemozoin (β-hematin) formation, mitochondrial bc1 complex, and DNA topoisomerase II.^8,61^ The selective accumulation of **5a** in *P. falciparum*-infected erythrocytes promotes simultaneous reduction in the red fluorescence of TMRE, indicating that it collapses the membrane potential (Figure 5C). Biagini et al.^29^ showed that deshydroxy-1-imino derivatives of acridine (e.g., dihydroacridinediones) cause a decrease of approximately 20% in total cellular-dependent TMRE fluorescence, suggesting a potential impact on mitochondrial function. In addition, dihydroacridinediones were potent inhibitors of the parasite’s mitochondrial bc1 complex (IC_50_ ∼ 15 nM).^29^ Dodean et al.^62^ developed a series of acridone-based inhibitors as broad-spectrum antimalarials. The compounds inhibited the *in vitro* *P. falciparum* blood-stage growth against multidrug-resistant parasite in the picomolar range; however, they did not identify the site(s) of action of these inhibitors.

Compound **5a** inhibited the *P. falciparum* cytochrome bc1 complex, while pyronaridine (**1a**) showed no inhibitory effect up to 200 μM (Figure 6A). The proposed mode of action observed for compound **5a** aligns with that of atovaquone and may account for their comparable antiplasmodial profiles observed in both speed-of-action and cross-resistance assays, as well as their synergistic interaction in combination with proguanil. Nonetheless, compound **5a** showed comparable potency against parasite strains carrying mutations in the Qo (Dd2Polδ_L144S) and Qi (Dd2Polδ_I22L) binding sites of the cytochrome bc1 complex (Figure 6B) but showed substantial resistance levels to the Dd2_V259L strain, which possesses a mutation in the Qo site.^42^ These findings indicate that while **5a** may share some resistance mechanisms with cytochrome bc1 complex and DHODH inhibitors, its binding mode likely differs from that of atovaquone, possibly binding to a distinct subsite or a partially overlapping region within the cytochrome bc1 complex. The further assessment of this is outside the scope of the current study.

Intracellular localization analysis indicated that **2d** accumulated in two small spheres surrounding the DV (Figure 5). These spherical structures are referred to as lipid bodies^63^ for which evidence suggests a role in heme detoxification within the parasite.^64^ In addition, the β-hematin inhibition assay indicated that **2d** acts by blocking hemozoin formation in the parasite (Figure 6C). This likely mode of action has been proposed for pyronaridine and acridine derivatives and is related to the accumulation of toxic hematin within the parasite’s food vacuole.^61^

To better understand the different modes of action for pyronaridine (**1a**) and compound **5a**, we conducted *in vitro* selection studies (Figure 7). While our attempts to generate **1a**-resistant parasites were unsuccessful, recrudescent parasites emerged within 21 days for compound **5a**, which was 8 to 25 times less potent against these recrudescent parasites. WGS analysis for 7 clones (out of 18) identified the mutations M521I in the TCP-1 gene, I1264N in the putative lipase gene, and M42I in the GS gene that could be related to the resistance phenotype. These data were confirmed for the remaining 11 clones by Sanger sequencing, which revealed two types of potential resistance mechanisms: mutations in TCP-1 and lipase putative genes and mutations in the GS gene (Tables S2 and S3).

Transcriptomic studies identified a correlation between artemisinin partial resistance (APR) and increased expression of unfolded protein response (UPR) pathways, including TCP-1 ring complex (TRiC),^65,66^ previously described to participate in the UPR of other species. Moreover, up-regulation of pathways associated with protein export or turnover, lipid metabolism and transport, and mitochondrial electron transport chain were observed in *k13* gene-edited isogenic parasites in the absence of dihydroartemisinin (DHA).^67^ Mok et al.^67^ reported an increase in redox enzyme expression after 48 h of DHA treatment, suggesting an interconnected mechanism, including enhanced ability to eliminate damaged proteins via the UPR response and ubiquitination, remodeling of secretory and vesicular transport that impact hemoglobin endocytosis, and also protein and lipid trafficking, for parasites to survive DHA treatment.^67^ The slow-acting profile of compound **5a**, particularly its effect on stalled parasite development at the ring stage after 48 h exposure (Figure 2B), in conjunction with the up-regulation of TCP-1, lipid metabolism, and oxidative stress responses associated with APR, raises the question of whether compound **5a** elicits resistance mechanisms as complex as (and possibly like) artemisinin derivatives. However, to shed further light on the resistance mechanisms identified in our study, a deeper investigation of the roles of TCP-1, lipase, and GS genes on this phenotype would need to be conducted in the future (e.g., using CRISPR).

In addition to our detailed analysis of the *in vitro* and *ex vivo* properties of the AC-based compounds, we assessed the *in vivo* antimalarial activity of compound **2d** by using a *P. berghei* model. It is noteworthy that compound **2d** exhibited a 100% reduction in parasitemia in the mice-treated group comparable to pyronaridine (**1a**) and chloroquine controls (Figure 8). The survival of mice treated with **2d** was comparable to that observed for the groups treated with both of the controls. These findings are considerably greater than the minimum threshold used for classifying a compound as active in an *in vivo* experiment (greater than 30%),^68^ suggesting that treatment with **2d** protected the animals from *Plasmodium* infection.

## Conclusions

We have synthesized 18 synthetic compounds belonging to two series: pyronaridine and quinacrine derivatives. We identified derivatives with potent and selective properties, exhibiting comparable or enhanced SI compared to pyronaridine and quinacrine and additionally had similar or improved *in vitro* ADME properties. The most promising candidates were found within the pyronaridine class, demonstrating activity against multidrug-resistant *P. falciparum* erythrocytic-stage parasites, exhibiting equipotent *ex vivo* activity against *P. vivax* and *P. falciparum* Brazilian isolates, and achieving desired therapeutic outcomes in an *in vivo* mouse model. Moreover, mode of action investigations of acridine/acridone-based compounds were consistent with a distinct mechanism. Specifically, the acridone-based **5a** targets the mitochondrial electron transport chain at the bc1 complex (like atovaquone) in *P. falciparum*, while acridine-based **2d** localizes near the parasite’s digestive vacuole and supports it acting as an inhibitor of hemozoin formation. The synergistic combination of compound **5a** with proguanil positions acridone derivatives as potential new agents for chemoprotection, filling a crucial gap in available options (primarily represented by atovaquone-proguanil) and signaling the need for further exploration. *In vitro* evolution studies conducted on compound **2d** failed to generate resistant parasites, thereby suggesting a low propensity for resistance. Conversely, parasites resistant to **5a** were obtained using the same protocol. These resistant parasites exhibited mutations in T-Complex protein 1 (TCP-1), lipase putative and glutathione synthetase (GS) genes, indicating a possibly complex mechanism of resistance like that observed with artemisinin. These combined findings offer invaluable insights that can guide the development of the next generation of potential clinical candidates based on AC compounds, paving the way for further novel and effective treatments for malaria.

## Methods

### Chemistry: Synthesis of Pyronaridine Derivatives for Antimalarial Evaluation

The synthetic procedures and physicochemical properties in our previous paper for compounds **1b**, **2d**–**j**, **3a**,**b,** and **4b–d**, have been described by Jones et al.^69^ and for compounds **2a**–**c** by Puhl et al.,^70^ and for compound **5a** by Dodean et al.^62^

### *P. falciparum* Parasite Culture

The *P. falciparum* strains were cultured in human erythrocytes maintained in RPMI 1640 medium (Sigma-Aldrich), supplemented with 0.2% NaHCO_3_, 25 mM HEPES, 11 mM d-glucose, 10 mg/L hypoxanthine, 25 mg/L gentamicin, and 0.5% (m/v) AlbuMAX II, essentially as previously described.^71^ The culture medium was routinely changed daily, and culture flasks were maintained under a 90% N_2_, 5% CO_2_, 5% O_2_ gas mixture at 37 °C.

### *In Vitro* Antiplasmodial Activity and Cross-Resistance Studies

The antiplasmodial activity of the AC-based compounds was assessed against *P. falciparum* blood parasites 3D7 strain. Parasites were synchronized to the ring stage through sorbitol treatment,^72^ and the density of parasites was determined using the SYBR Green I method.^73^ The antiplasmodial activity of test compounds was evaluated against a representative panel of *P. falciparum*-resistant (Dd2, K1, 3D7^R^_MMV848, TM90C6B, Dd2_V259L, Dd2-Polδ_I22L, Dd2-Polδ_L144S, and SB1-A6) strains. A resistance index (RI) was calculated by the ratio of IC_50_^Resistant strain^ to IC_50_^3D7^. RI > 5 was considered indicative of cross-resistance.^74^ The study was approved by the Research Ethics Committee (CAAE 67642722.50000.5505). The details of the protocol used are described in the Supporting Information Methods.

### *Ex Vivo* Clinical Isolate Schizont Maturation Assay

Clinical isolates of *P. falciparum* and *P. vivax* were obtained in September and October 2024 from patients enrolled at the Centre of Malaria Control in Porto Velho, Brazil. The study received ethical approval from the Centro de Pesquisa em Medicina Tropical (CEPEM) ethics committee (CAAE 58738416.1.0000.0011). Test compounds, along with standard antimalarials such as artesunate and chloroquine, were assessed on 7 *P. vivax* and 6 *P. falciparum* isolates, all of which were subjected to compound incubation for ≥40 h. The details of the protocol used are described in the Supporting Information.

### Speed-of-Action Assay

To determine whether test compounds acted as fast- or slow-acting inhibitors, a protocol adapted from Le Manach et al.^38^ was used. The details of the protocol used are described in the Supporting Information Methods.

### Activity in Combination with Proguanil

Drug combination assays were conducted following the methodology outlined by Fivelman et al.^75^ Additivity was assessed using the Hand model,^76^ with fractional inhibitory concentration (FIC_50_) values calculated for seven different compound proportions, expressed in terms of IC_50_ equivalents. FIC_50_ values from three independent experiments were subjected to nonlinear fitting and statistically compared to the additivity isobole. The details of the protocol used are described in the Supporting Information Methods.

### Intracellular Localization Studies by Confocal Microscopy

Erythrocytes infected with *P. falciparum* (3D7 strain) nonsynchronous parasites were washed in MOPS buffer, resuspended in the same buffer, and plated on a microscopy chamber previously pretreated with L-polylysine. The localization of the autofluorescent AC-based compound **2d** was achieved by adding 10 μM of it to the cells immobilized in the chamber. Additionally, the tetramethyl rhodamine ethyl ester (TMRE) was used to assess whether the autofluorescent AC-based compound **5a** affected the membrane potential of mitochondria in malaria-infected red blood cells. The cells immobilized in the chamber were loaded with 100 nM TMRE, and subsequently, compound **5a** (10 μM) was added. Membrane potential-dependent fluorescence changes were then monitored in real time. The details of the protocol used are described in the Supporting Information.

### *In Vivo* Assay against *P. berghei*

A suppressive parasite growth test was performed in mice infected with *P. berghei* NK65 strain (originally received from the New York University Medical School), as described previously.^77^ The use of laboratory animals was approved by the Ethics Committee for Animal Use of Universidade Federal do Estado de São Paulo, UNIFESP (CEUA N 6630080816). The details of the protocol used are described in the Supporting Information.

### Inhibition Cytochrome bc1 Complex Assay

*P. falciparum* mitochondria were extracted from isolated parasites with some modifications.^78^ The details of the protocol used are described in the Supporting Information.

### β-Hematin Inhibition Assay

The β-hematin inhibition assay was performed as previously described.^79^ The details of the protocol used are described in Supporting Information.

### Selecting for Resistance in *P. falciparum* *In Vitro* and Sequencing

The generation of resistance parasites was performed according to Paquet et al. with some modifications.^54^ The details of the protocol used are described in the Supporting Information.

### *P. falciparum* Stage-Specificity Assay

To identify the asexual blood stage most susceptible to the tested compounds, we followed a previously established protocol.^80^ The details of the protocol used are described in the Supporting Information.

### *In Silico* and *In Vitro* ADME Assays

ADMET properties were predicted using our in-house Assay Central software and models.^46^ These were followed by *in vitro* assays for Caco-2 cell permeability, kinetic solubility testing, mouse and human liver microsome stability, mouse and human plasma stability, and mouse and human plasma protein binding performed by a CRO (Syngene) as described in the Supporting Information.

## Supplementary Material



## Notes

General procedure for the synthesis of acridines **4a**–**d**; details of the protocol used (biology); hemolytic activity of compounds **1a**,**b**, **2a**–**j**, **3a**,**b, 4a**–**d**, and **5a**; isobolograms of association of **1b**, **2a**, **2b**, and **4a** with proguanil; ^1^H and ^13^C NMR spectra, HR-MS data, and HPLC trace chromatograms of compounds **4a**–**d**, **5a**–**b**, **6**, and **7** (PDF)

## References

[1] World Malaria Report 2025: Addressing the Threat of Antimalarial Drug Resistance; World Health Organization: Geneva, 2025. https://www.who.int/teams/global-malaria-programme/reports/world-malaria-report-2025 (accessed 2026–01–13).

[2] Cowman A. F., Healer J., Marapana D., Marsh K. (2016). Malaria: Biology and
Disease. Cell.

[3] Snounou G., Sharp P. M., Culleton R. (2024). The Two Parasite
Species Formerly
Known as Plasmodium Ovale. Trends Parasitol..

[4] Witkowski B., Khim N., Chim P., Kim S., Ke S., Kloeung N., Chy S., Duong S., Leang R., Ringwald P., Dondorp A. M., Tripura R., Benoit-Vical F., Berry A., Gorgette O., Ariey F., Barale J.-C., Mercereau-Puijalon O., Menard D. (2013). Reduced Artemisinin Susceptibility
of Plasmodium Falciparum Ring Stages in Western Cambodia. Antimicrob. Agents Chemother..

[5] Balikagala B., Fukuda N., Ikeda M., Katuro O. T., Tachibana S.-I., Yamauchi M., Opio W., Emoto S., Anywar D. A., Kimura E., Palacpac N. M. Q., Odongo-Aginya E. I., Ogwang M., Horii T., Mita T. (2021). Evidence of Artemisinin-Resistant
Malaria in Africa. N. Engl. J. Med..

[6] Wilby K. J., Lau T. T. Y., Gilchrist S. E., Ensom M. H. H. (2012). Mosquirix (RTS,S):
A Novel Vaccine for the Prevention of Plasmodium Falciparum Malaria. Ann. Pharmacother..

[7] Laurens M. B. (2020). RTS,S/AS01
Vaccine (Mosquirix^TM^): An Overview. Hum. Vaccin. Immunother..

[8] Valdés A. F.-C. (2011). Acridine
and Acridinones: Old and New Structures with Antimalarial Activity. Open Med. Chem. J..

[9] Wainwright M. (2008). Dyes in the
Development of Drugs and Pharmaceuticals. Dyes
Pigm..

[10] Kitchen L. W., Vaughn D. W., Skillman D. R. (2006). Role of US Military
Research Programs
in the Development of US Food and Drug Administration--Approved Antimalarial
Drugs. Clin. Infect. Dis..

[11] Auparakkitanon S., Noonpakdee W., Ralph R. K., Denny W. A., Wilairat P. (2003). Antimalarial
9-Anilinoacridine Compounds Directed AtHematin. Antimicrob. Agents Chemother..

[12] Chibale K., Haupt H., Kendrick H., Yardley V., Saravanamuthu A., Fairlamb A. H., Croft S. L. (2001). Antiprotozoal
and Cytotoxicity Evaluation
of Sulfonamide and Urea Analogues of Quinacrine. Bioorg. Med. Chem. Lett..

[13] Sparatore A., Basilico N., Parapini S., Romeo S., Novelli F., Sparatore F., Taramelli D. (2005). 4-Aminoquinoline Quinolizidinyl-
and Quinolizidinylalkyl-Derivatives with Antimalarial Activity. Bioorg. Med. Chem..

[14] Anderson M. O., Sherrill J., Madrid P. B., Liou A. P., Weisman J. L., DeRisi J. L., Guy R. K. (2006). Parallel
Synthesis of 9-Aminoacridines
and Their Evaluation against Chloroquine-Resistant Plasmodium Falciparum. Bioorg. Med. Chem..

[15] Fonte M., Fagundes N., Gomes A., Ferraz R., Prudêncio C., Araújo M. J., Gomes P., Teixeira C. (2019). Development of a Synthetic
Route towards N4,N9-Disubstituted 4,9-Diaminoacridines: On the Way
to Multi-Stage Antimalarials. Tetrahedron Lett..

[16] Guetzoyan L., Yu X.-M., Ramiandrasoa F., Pethe S., Rogier C., Pradines B., Cresteil T., Perrée-Fauvet M., Mahy J.-P. (2009). Antimalarial Acridines: Synthesis, in Vitro Activity
against P. Falciparum and Interaction with Hematin. Bioorg. Med. Chem..

[17] Yu X.-M., Ramiandrasoa F., Guetzoyan L., Pradines B., Quintino E., Gadelle D., Forterre P., Cresteil T., Mahy J.-P., Pethe S. (2012). Synthesis
and Biological Evaluation of Acridine Derivatives as Antimalarial
Agents. ChemMedChem..

[18] Zheng X. Y., Xia Y., Gao F. H., Chen C. (1979). Synthesis of 7351, a New Antimalarial
Drug (Author’s Transl). Yao Xue Xue Bao
= Acta Pharm. Sin..

[19] Croft S. L., Duparc S., Arbe-Barnes S. J., Craft J. C., Shin C.-S., Fleckenstein L., Borghini-Fuhrer I., Rim H.-J. (2012). Review of Pyronaridine
Anti-Malarial Properties and Product Characteristics. Malar. J..

[20] Pryce J., Hine P. (2019). Pyronaridine-Artesunate
for Treating Uncomplicated Plasmodium Falciparum
Malaria. Cochrane Database Syst. Rev..

[21] Qi J., Wang S., Liu G., Peng H., Wang J., Zhu Z., Yang C. (2004). Pyronaridine,
a Novel Modulator of P-Glycoprotein-Mediated
Multidrug Resistance in Tumor Cells in Vitro and in Vivo. Biochem. Biophys. Res. Commun..

[22] Rank L., Puhl A. C., Havener T. M., Anderson E., Foil D. H., Zorn K. M., Monakhova N., Riabova O., Hickey A. J., Makarov V., Ekins S. (2022). Multiple Approaches
to Repurposing
Drugs for Neuroblastoma. Bioorg. Med. Chem..

[23] Villanueva P. J., Gutierrez D. A., Contreras L., Parra K., Segura-Cabrera A., Varela-Ramirez A., Aguilera R. J. (2021). The Antimalarial Drug Pyronaridine
Inhibits Topoisomerase II in Breast Cancer Cells and Hinders Tumor
Progression In Vivo. Clin. Cancer Drugs.

[24] El-Sayed S. A. E.-S., Rizk M. A., Ringo A. E., Li Y., Liu M., Ji S., Li J., Byamukama B., Tumwebaze M. A., Xuan X., Igarashi I. (2021). Impact of Using Pyronaridine Tetraphosphate-
Based Combination Therapy in the Treatment of Babesiosis Caused by
Babesia Bovis, B. Caballi, and B. Gibsoni in Vitro and B. Microti
in Mice. Parasitol. Int..

[25] Ekins S., de Siqueira-Neto J. L., McCall L.-I., Sarker M., Yadav M., Ponder E. L., Kallel E. A., Kellar D., Chen S., Arkin M., Bunin B. A., McKerrow J. H., Talcott C. (2015). Machine Learning
Models and Pathway Genome Data Base for Trypanosoma Cruzi Drug Discovery. PLoS Neglected Trop. Dis..

[26] Lane T. R., Massey C., Comer J. E., Anantpadma M., Freundlich J. S., Davey R. A., Madrid P. B., Ekins S. (2019). Repurposing
the Antimalarial Pyronaridine Tetraphosphate to Protect against Ebola
Virus Infection. PLoS Negl. Trop. Dis..

[27] Mori G., Orena B. S., Franch C., Mitchenall L. A., Godbole A. A., Rodrigues L., Aguilar-Pérez C., Zemanová J., Huszár S., Forbak M., Lane T. R., Sabbah M., Deboosere N., Frita R., Vandeputte A., Hoffmann E., Russo R., Connell N., Veilleux C., Jha R. K., Kumar P., Freundlich J. S., Brodin P., Aínsa J.
A., Nagaraja V., Maxwell A., Mikušová K., Pasca M. R., Ekins S. (2018). The EU Approved Antimalarial Pyronaridine Shows Antitubercular Activity
and Synergy with Rifampicin. Targeting RNA Polymerase.
Tuberculosis.

[28] Fong K. Y., Wright D. W. (2013). Hemozoin and Antimalarial Drug Discovery. Future Med. Chem..

[29] Biagini G. A., Fisher N., Berry N., Stocks P. A., Meunier B., Williams D. P., Bonar-Law R., Bray P. G., Owen A., O’Neill P. M., Ward S. A. (2008). Acridinediones: Selective and Potent
Inhibitors of the Malaria Parasite Mitochondrial *Bc*
_1_ Complex. Mol. Pharmacol..

[30] Valluri H., Bhanot A., Shah S., Bhandaru N., Sundriyal S. (2023). Basic Nitrogen
(BaN) Is a Key Property of Antimalarial Chemical Space. J. Med. Chem..

[31] Cheruku S. R., Maiti S., Dorn A., Scorneaux B., Bhattacharjee A. K., Ellis W. Y., Vennerstrom J. L. (2003). Carbon
Isosteres of the 4-Aminopyridine Substructure of Chloroquine: Effects
on PK­(a), Hematin Binding, Inhibition of Hemozoin Formation, and Parasite
Growth. J. Med. Chem..

[32] Chugh M., Scheurer C., Sax S., Bilsland E., van Schalkwyk D. A., Wicht K. J., Hofmann N., Sharma A., Bashyam S., Singh S., Oliver S. G., Egan T. J., Malhotra P., Sutherland C. J., Beck H.-P., Wittlin S., Spangenberg T., Ding X. C. (2015). Identification and Deconvolution of Cross-Resistance
Signals from Antimalarial Compounds Using Multidrug-Resistant Plasmodium
Falciparum Strains. Antimicrob. Agents Chemother..

[33] Irabuena C., Scarone L., de Souza G. E., Aguiar A. C. C., Mendes G. R., Guido R. V. C., Serra G. (2022). Synthesis and Antiplasmodial Assessment
of Nitazoxanide and Analogs as New Antimalarial Candidates. Med. Chem. Res..

[34] Winter R. W., Kelly J. X., Smilkstein M. J., Dodean R., Bagby G. C., Rathbun R. K., Levin J. I., Hinrichs D., Riscoe M. K. (2006). Evaluation
and Lead Optimization of Anti-Malarial Acridones. Exp. Parasitol..

[35] Neafsey D. E., Schaffner S. F., Volkman S. K., Park D., Montgomery P., Milner D. A., Lukens A., Rosen D., Daniels R., Houde N., Cortese J. F., Tyndall E., Gates C., Stange-Thomann N., Sarr O., Ndiaye D., Ndir O., Mboup S., Ferreira M. U., Moraes S. do L., Dash A. P., Chitnis C. E., Wiegand R. C., Hartl D. L., Birren B. W., Lander E. S., Sabeti P. C., Wirth D. F. (2008). Genome-Wide
SNP
Genotyping Highlights the Role of Natural Selection in Plasmodium
Falciparum Population Divergence. Genome Biol..

[36] Aguiar A. C. C., Pereira D. B., Amaral N. S., De Marco L., Krettli A. U. (2014). Plasmodium
Vivax and Plasmodium Falciparum Ex Vivo Susceptibility to Anti-Malarials
and Gene Characterization in Rondônia, West Amazon, Brazil. Malar. J..

[37] Burrows J. N., Duparc S., Gutteridge W. E., Hooft van Huijsduijnen R., Kaszubska W., Macintyre F., Mazzuri S., Möhrle J. J., Wells T. N. C. (2017). New Developments in Anti-Malarial Target Candidate
and Product Profiles. Malar. J..

[38] Le
Manach C., Scheurer C., Sax S., Schleiferböck S., Cabrera D. G., Younis Y., Paquet T., Street L., Smith P., Ding X. C., Waterson D., Witty M. J., Leroy D., Chibale K., Wittlin S. (2013). Fast in Vitro Methods
to Determine the Speed of Action and the Stage-Specificity of Anti-Malarials
in Plasmodium Falciparum. Malar. J..

[39] Mendes G. R., Noronha A. L., Moura I. M. R., Moreira N. M., Bonatto V., Barbosa C. S., Maluf S. E. C., de
Souza G. E., de Amorim M. R., Aguiar A. C. C., Cruz F. C., Ferreira A. D. S., Teles C. B. G., Pereira D. B., Hajdu E., Ferreira A. G., Berlinck R. G. S., Guido R. V. C. (2025). Marine Guanidine
Alkaloids Inhibit Malaria Parasites
Development in In Vitro, In Vivo and Ex Vivo Assays. ACS Infect. Dis..

[40] Berman J. D., Nielsen R., Chulay J. D., Dowler M., Kain K. C., Kester K. E., Williams J., Whelen A. C., Shmuklarsky M. J. (2001). Causal
Prophylactic Efficacy of Atovaquone-Proguanil (MalaroneTM) in a Human
Challenge Model. Trans. R. Soc. Trop. Med. Hyg..

[41] Siregar J. E., Kurisu G., Kobayashi T., Matsuzaki M., Sakamoto K., Mi-ichi F., Watanabe Y., Hirai M., Matsuoka H., Syafruddin D., Marzuki S., Kita K. (2015). Direct Evidence
for the Atovaquone Action on the Plasmodium Cytochrome Bc 1 Complex. Parasitol. Int..

[42] Calit J., Prajapati S. K., Benavente E. D., Araújo J. E., Deng B., Miura K., Annunciato Y., Moura I. M. R., Usui M., Medeiros J. F., Andrade C. H., Silva-Mendonça S., Simeonov A., Eastman R. T., Long C. A., da Silva Araujo M., Williamson K. C., Aguiar A. C. C., Bargieri D. Y. (2024). Pyrimidine Azepine
Targets the Plasmodium
Bc 1 Complex and Displays Multistage Antimalarial Activity. JACS Au.

[43] Stickles A. M., de Almeida M. J., Morrisey J. M., Sheridan K. A., Forquer I. P., Nilsen A., Winter R. W., Burrows J. N., Fidock D. A., Vaidya A. B., Riscoe M. K. (2015). Subtle Changes in Endochin-like Quinolone
Structure Alter the Site of Inhibition within the Cytochrome Bc1 Complex
of Plasmodium Falciparum. Antimicrob. Agents
Chemother..

[44] Painter H. J., Morrisey J. M., Mather M. W., Orchard L. M., Luck C., Smilkstein M. J., Riscoe M. K., Vaidya A. B., Llinás M. (2021). Atypical Molecular
Basis for Drug Resistance to Mitochondrial Function Inhibitors in
Plasmodium Falciparum. Antimicrob. Agents Chemother..

[45] Kümpornsin K., Kochakarn T., Yeo T., Okombo J., Luth M. R., Hoshizaki J., Rawat M., Pearson R. D., Schindler K. A., Mok S., Park H., Uhlemann A.-C., Jana G. P., Maity B. C., Laleu B., Chenu E., Duffy J., Moliner
Cubel S., Franco V., Gomez-Lorenzo M. G., Gamo F. J., Winzeler E. A., Fidock D. A., Chookajorn T., Lee M. C. S. (2023). Generation of a Mutator Parasite to Drive Resistome
Discovery in Plasmodium Falciparum. Nat. Commun..

[46] Lane T., Russo D. P., Zorn K. M., Clark A. M., Korotcov A., Tkachenko V., Reynolds R. C., Perryman A. L., Freundlich J. S., Ekins S. (2018). Comparing and Validating Machine Learning Models for Mycobacterium
Tuberculosis Drug Discovery. Mol. Pharmaceutics.

[47] Zdrazil B., Felix E., Hunter F., Manners E. J., Blackshaw J., Corbett S., de Veij M., Ioannidis H., Lopez D. M., Mosquera J. F., Magarinos M. P., Bosc N., Arcila R., Kizilören T., Gaulton A., Bento A. P., Adasme M. F., Monecke P., Landrum G. A., Leach A. R. (2024). The ChEMBL Database in 2023: A Drug
Discovery Platform Spanning Multiple Bioactivity Data Types and Time
Periods. Nucleic Acids Res..

[48] Aniceto N., Freitas A. A., Bender A., Ghafourian T. (2016). A Novel Applicability
Domain Technique for Mapping Predictive Reliability across the Chemical
Space of a QSAR: Reliability-Density Neighbourhood. J. Cheminform..

[49] Ward K. E., Fidock D. A., Bridgford J. L. (2022). Plasmodium
Falciparum Resistance
to Artemisinin-Based Combination Therapies. Curr. Opin. Microbiol..

[50] Gerets H. H. J., Tilmant K., Gerin B., Chanteux H., Depelchin B. O., Dhalluin S., Atienzar F. A. (2012). Characterization
of Primary Human
Hepatocytes, HepG2 Cells, and HepaRG Cells at the MRNA Level and CYP
Activity in Response to Inducers and Their Predictivity for the Detection
of Human Hepatotoxins. Cell Biol. Toxicol..

[51] Burrows J. N., Leroy D., Lotharius J., Waterson D. (2011). Challenges in Antimalarial
Drug Discovery. Future Med. Chem..

[52] Basco L. K., Le Bras J. (1992). In Vitro Activity of
Pyronaridine against African Strains
of Plasmodium Falciparum. Ann. Trop. Med. Parasitol..

[53] Elueze E. I., Croft S. L., Warhurst D. C. (1996). Activity of Pyronaridine and Mepacrine
against Twelve Strains of Plasmodium Falciparum in Vitro. J. Antimicrob. Chemother..

[54] Paquet T., Le Manach C., Cabrera D. G., Younis Y., Henrich P. P., Abraham T. S., Lee M. C. S., Basak R., Ghidelli-Disse S., Lafuente-Monasterio M. J., Bantscheff M., Ruecker A., Blagborough A. M., Zakutansky S. E., Zeeman A. M., White K. L., Shackleford D. M., Mannila J., Morizzi J., Scheurer C., Angulo-Barturen I., Santosmartínez M., Ferrer S., Sanz L. M., Gamo F. J., Reader J., Botha M., Dechering K. J., Sauerwein R. W., Tungtaeng A., Vanachayangkul P., Lim C. S., Burrows J., Witty M. J., Marsh K. C., Bodenreider C., Rochford R., Solapure S. M., Jiménez-Díaz M. B., Wittlin S., Charman S. A., Donini C., Campo B., Birkholtz L. M., Khanson K., Drewes G., Kocken C. M., Delves M. J., Leroy D., Fidock D. A., Waterson D., Street L. J., Chibale K. (2017). Antimalarial Efficacy of MMV390048,
an Inhibitor of Plasmodium Phosphatidylinositol 4-Kinase. Sci. Transl. Med..

[55] Kancharla P., Dodean R. A., Li Y., Pou S., Pybus B., Melendez V., Read L., Bane C. E., Vesely B., Kreishman-Deitrick M., Black C., Li Q., Sciotti R. J., Olmeda R., Luong T.-L., Gaona H., Potter B., Sousa J., Marcsisin S., Caridha D., Xie L., Vuong C., Zeng Q., Zhang J., Zhang P., Lin H., Butler K., Roncal N., Gaynor-Ohnstad L., Leed S. E., Nolan C., Ceja F. G., Rasmussen S. A., Tumwebaze P. K., Rosenthal P. J., Mu J., Bayles B. R., Cooper R. A., Reynolds K. A., Smilkstein M. J., Riscoe M. K., Kelly J. X. (2020). Lead Optimization
of Second-Generation
Acridones as Broad-Spectrum Antimalarials. J.
Med. Chem..

[56] Dodean R. A., Li Y., Zhang X., Kumar A., Pou S., W Winter R., Liebman K. M., Zakharov L. N., Caridha D., Madejczyk M. S., Vuong C., DeLuca J., Chin G., Kudyba K., McEnearney S., Jin X., Dennis W. E., Chetree R., Blount C., Pannone K., Dinh H. T., Mdaki K., Leed S., Lee P. J., Roth A., Kancharla P., Kelly J. X. (2025). Acridone Prodrugs with Enhanced Dual-Stage
Antimalarial
Efficacy. ACS Med. Chem. Lett..

[57] Suswam E., Kyle D., Lang-Unnasch N. (2001). Plasmodium
Falciparum: The Effects
of Atovaquone Resistance on Respiration. Exp.
Parasitol..

[58] Foguim F. T., Robert M. G., Gueye M. W., Gendrot M., Diawara S., Mosnier J., Amalvict R., Benoit N., Bercion R., Fall B., Madamet M., Pradines B. (2019). Low Polymorphisms in
Pfact, Pfugt and Pfcarl Genes in African Plasmodium Falciparum Isolates
and Absence of Association with Susceptibility to Common Anti-Malarial
Drugs. Malar. J..

[59] Li J., Zhang J., Li Q., Hu Y., Ruan Y., Tao Z., Xia H., Qiao J., Meng L., Zeng W., Li C., He X., Zhao L., Siddiqui F. A., Miao J., Yang Z., Fang Q., Cui L. (2020). Ex Vivo Susceptibilities
of Plasmodium Vivax Isolates from the China-Myanmar Border to Antimalarial
Drugs and Association with Polymorphisms in Pvmdr1 and Pvcrt-o Genes. PLoS Negl. Trop. Dis..

[60] Korsinczky M., Chen N., Kotecka B., Saul A., Rieckmann K., Cheng Q. (2000). Mutations in *Plasmodium Falciparum* Cytochrome *b* That
Are Associated with Atovaquone Resistance Are Located
at a Putative Drug-Binding Site. Antimicrob.
Agents Chemother..

[61] Bailly C. (2021). Pyronaridine:
An Update of Its Pharmacological Activities and Mechanisms of Action. Biopolymers.

[62] Dodean R. A., Kancharla P., Li Y., Melendez V., Read L., Bane C. E., Vesely B., Kreishman-Deitrick M., Black C., Li Q., Sciotti R. J., Olmeda R., Luong T.-L., Gaona H., Potter B., Sousa J., Marcsisin S., Caridha D., Xie L., Vuong C., Zeng Q., Zhang J., Zhang P., Lin H., Butler K., Roncal N., Gaynor-Ohnstad L., Leed S. E., Nolan C., Huezo S. J., Rasmussen S. A., Stephens M. T., Tan J. C., Cooper R. A., Smilkstein M. J., Pou S., Winter R. W., Riscoe M. K., Kelly J. X. (2019). Discovery and Structural
Optimization of Acridones as Broad-Spectrum Antimalarials. J. Med. Chem..

[63] Vallochi A. L., Teixeira L., Oliveira K. D. S., Maya-Monteiro C. M., Bozza P. T. (2018). Lipid Droplet, a
Key Player in Host-Parasite Interactions. Front.
Immunol..

[64] Jackson K. E., Klonis N., Ferguson D. J. P., Adisa A., Dogovski C., Tilley L. (2004). Food Vacuole-associated Lipid Bodies
and Heterogeneous
Lipid Environments in the Malaria Parasite. Plasmodium Falciparum. Mol. Microbiol..

[65] Mok S., Ashley E. A., Ferreira P. E., Zhu L., Lin Z., Yeo T., Chotivanich K., Imwong M., Pukrittayakamee S., Dhorda M., Nguon C., Lim P., Amaratunga C., Suon S., Hien T. T., Htut Y., Faiz M. A., Onyamboko M. A., Mayxay M., Newton P. N., Tripura R., Woodrow C. J., Miotto O., Kwiatkowski D. P., Nosten F., Day N. P. J., Preiser P. R., White N. J., Dondorp A. M., Fairhurst R. M., Bozdech Z. (2015). Population Transcriptomics
of Human Malaria Parasites Reveals the Mechanism of Artemisinin Resistance. Science..

[66] Zhu L., van der Pluijm R. W., Kucharski M., Nayak S., Tripathi J., White N. J., Day N. P. J., Faiz A., Phyo A. P., Amaratunga C., Lek D., Ashley E. A., Nosten F., Smithuis F., Ginsburg H., von Seidlein L., Lin K., Imwong M., Chotivanich K., Mayxay M., Dhorda M., Nguyen H. C., Nguyen T. N. T., Miotto O., Newton P. N., Jittamala P., Tripura R., Pukrittayakamee S., Peto T. J., Hien T. T., Dondorp A. M., Bozdech Z. (2022). Artemisinin
Resistance in the Malaria Parasite, Plasmodium Falciparum, Originates
from Its Initial Transcriptional Response. Commun.
Biol..

[67] Mok S., Stokes B. H., Gnädig N. F., Ross L. S., Yeo T., Amaratunga C., Allman E., Solyakov L., Bottrill A. R., Tripathi J., Fairhurst R. M., Llinás M., Bozdech Z., Tobin A. B., Fidock D. A. (2021). Artemisinin-Resistant
K13 Mutations Rewire Plasmodium Falciparum’s Intra-Erythrocytic
Metabolic Program to Enhance Survival. Nat.
Commun..

[68] Andrade-Neto V. F., Brandão M. G. L., Stehmann J. R., Oliveira L. A., Krettli A. U. (2003). Antimalarial
Activity of Cinchona-like Plants Used to Treat Fever and Malaria in
Brazil. J. Ethnopharmacol..

[69] Jones T., Monakhova N., Guivel-Benhassine F., Lepioshkin A., Bruel T., Lane T. R., Schwartz O., Puhl A. C., Makarov V., Ekins S. (2023). Synthesis
and Evaluation of 9-Aminoacridines
with SARS-CoV-2 Antiviral Activity. ACS Omega.

[70] Puhl A. C., Gomes G. F., Damasceno S., Godoy A. S., Noske G. D., Nakamura A. M., Gawriljuk V. O., Fernandes R. S., Monakhova N., Riabova O., Lane T. R., Makarov V., Veras F. P., Batah S. S., Fabro A. T., Oliva G., Cunha F. Q., Alves-Filho J. C., Cunha T. M., Ekins S. (2022). Pyronaridine
Protects against SARS-CoV-2 Infection in Mouse. ACS Infect. Dis..

[71] Trager W., Jensen J. B. (1976). Human Malaria Parasites
in Continuous Culture. Science (1979).

[72] Lambros C., Vanderberg J. P. (1979). Synchronization of Plasmodium Falciparum Erythrocytic
Stages in Culture. J. Parasitol..

[73] Johnson J. D., Dennull R. A., Gerena L., Lopez-Sanchez M., Roncal N. E., Waters N. C. (2007). Assessment and Continued
Validation
of the Malaria SYBR Green I-Based Fluorescence Assay for Use in Malaria
Drug Screening. Antimicrob. Agents Chemother..

[74] Katsuno K., Burrows J. N., Duncan K., van Huijsduijnen R. H., Kaneko T., Kita K., Mowbray C. E., Schmatz D., Warner P., Slingsby B. T. (2015). Hit and Lead Criteria in Drug Discovery
for Infectious Diseases of the Developing World. Nat. Rev. Drug Discovery.

[75] Fivelman Q. L., Adagu I. S., Warhurst D. C. (2004). Modified
Fixed-Ratio Isobologram
Method for Studying in Vitro Interactions between Atovaquone and Proguanil
or Dihydroartemisinin against Drug-Resistant Strains of Plasmodium
Falciparum. Antimicrob. Agents Chemother..

[76] Hand, D. J. Synergy in Drug Combinations. In Data Analysis. Studies in Classification, Data Analysis, and Knowledge Organization; Gaul, W. , Opitz, O. , Schader, M. , Eds.; Springer, Berlin, Heidelberg, 2000; pp 471–475. 10.1007/978-3-642-58250-9_38.

[77] Peters W. (1965). Drug Resistance
in Plasmodium Berghei Vincke and Lips, 1948. I. Chloroquine Resistance. Exp. Parasitol..

[78] Okada-Junior C. Y., Monteiro G. C., Aguiar A. C. C., Batista V. S., de Souza J. O., Souza G. E., Bueno R. V., Oliva G., Nascimento-Júnior N. M., Guido R. V. C., Bolzani V. S. (2018). Phthalimide Derivatives with Bioactivity
against *Plasmodium Falciparum*: Synthesis, Evaluation,
and Computational Studies Involving *Bc*
_1_ Cytochrome Inhibition. ACS Omega.

[79] Carter M. D., Phelan V. V., Sandlin R. D., Bachmann B. O., Wright D. W. (2010). Lipophilic
Mediated Assays for Beta-Hematin Inhibitors. Comb. Chem. High Throughput Screen..

[80] Murithi J. M., Owen E. S., Istvan E. S., Lee M. C. S., Ottilie S., Chibale K., Goldberg D. E., Winzeler E. A., Llinás M., Fidock D. A., Vanaerschot M. (2020). Combining
Stage Specificity and Metabolomic
Profiling to Advance Antimalarial Drug Discovery. Cell Chem. Biol..

