# Supplementary material for: Efficacy, Structure–Activity Relationship, and Mode of Action Studies of a New Generation of Acridine/Acridone-Based Antimalarials
Source: ACS Infect Dis. 2026 May 26;12(6):1866–83. doi: 10.1021/acsinfecdis.5c00682 (PMC13270508; doi:10.1021/acsinfecdis.5c00682)
Supplement: Supplementary file 1 [file id5c00682_si_001.pdf]

Supporting Information

For

The efficacy, structure-activity relationship, and mode of action studies of a new generation of acridine/acridone-based antimalarials

Sarah El Chamy Maluf [a§], Giovana Rossi Mendes [a§], Igor M. R. Moura [a§], Guilherme Eduardo de Souza [a§], Talita Alvarenga Valdes [a], Vinícius Bonatto [a], Gabriela Silva Oliveira [a], Anna Caroline Campos Aguiar [b], Marcos L. Gazarini [c], Ana C. Puhl [d], Natalia Monakhova [e], Alexander Lepioshkin [e], Vadim Makarov [e], Thomas R. Lane [d], Renuka Raman [d], Guilherme A. S. Campolina [b], Camila S. Barbosa [b], Amália dos Santos Ferreira [f], Carolina B. G. Teles [f], Dhelio B. Pereira [g], Roberto Rudge de Moraes Barros [b], Ernest Diez Benavente [h], Sean Ekins [d*], Rafael Victorio Carvalho Guido [a*]

[a] *São Carlos Institute of Physics, University of São Paulo, São Carlos, 13563-120, Brazil.*
[b] *Department of Microbiology, Immunology and Parasitology, Federal University of São Paulo, São Paulo, CEP 04023-062, Brazil*
[c] *Department of Biosciences, Federal University of São Paulo, Santos, 11015-020, Brazil*
[d] *Collaborations Pharmaceuticals, Inc., 840 Main Campus Drive, Lab 3510, Raleigh NC 27606, USA*
[e] *Research Center of Biotechnology RAS, 33-2 Leninsky prospect, 119071 Moscow, Russia*
[f] *Oswaldo Cruz Foundation, Leishmaniasis and Malaria Bioassay Platform, Porto Velho, 76812-245, Brazil*
[g] *Research Center in Tropical Medicine of Rondônia, Porto Velho, 76812-329, Brazil*
[h] *Laboratory of Experimental Cardiology, University Medical Center Utrecht, Utrecht University, Utrecht 3584 CS, The Netherlands*

[§] *S.E.C.M., G.R.M., I.M.R.M., and G.E.S. contributed equally to this work*

*Email: rvcguido@usp.br
*Email: sean@collaborationspharma.com

Experimental

METHODS - Chemistry....S5
General procedure for the synthesis of acridines 4a-d....S6
METHODS – Biology....S10
Hemolysis Assay....S10
In vitro antiplasmodial blood stage activity....S10
Cross-resistance studies....S11
Ex vivo clinical isolate schizont maturation assay....S11
Speed-of-Action assay....S12
Activity in combination with proguanil....S13
Intracellular localization studies by confocal microscopy....S14
In vivo assay against *Plasmodium berghei*....S14
Inhibition cytochrome bc1 complex assay....S15
Beta-hematin inhibition Assay....S16
Selecting for resistance in *P. falciparum in vitro*....S17
Sanger sequencing....S18
P. falciparum Stage-Specificity Assay....S18
Caco-2 cell permeability testing....S19
Kinetic solubility testing....S21
Mouse and human liver microsome stability....S21
Human and mouse plasma stability assay....S22
Human/mouse plasma protein binding assay....S22

List of Supplementary Tables

Table S1. *In vitro* antiplasmodial activity against chloroquine-sensitive (3D7) strain of *P. falciparum*, cytotoxicity against HepG2 cell line and selectivity index of quinacrine derivatives. Mean values are represented with their respective standard deviations....S24

Table S2. Candidate genes identified by whole-genome sequencing in 5a-resistant lineage....S25

Table S3. Summary of mutations identified by Sanger sequencing across isolated clones....S26

Table S4. Predicted *in vitro* ADME properties for **2d** (12126038). Highlighted has also been determined experimentally. Due to the nature of the prediction scores, typically a threshold of 0.5 is used to determine if a compound is predicted to be active, but higher scores suggest a higher likelihood of activity. Applicability scores are based on the reliability-density neighborhood (RDN) method which considers both the model overlap and the individual bias and precision of the overlapping fingerprints. There is not a defined threshold for an acceptable applicability score, but it is ideal to have a higher score for confidence in the prediction (1 max). The consensus scores are based on majority rule classification (agreement for ≥ 4 algorithms, when equal to 4 a compound is predicted as active). The threshold for activity is shown for each model....S27

Table S5. Predicted *in vitro* ADME properties for 2c (12126039). Highlighted has also been determined experimentally. Due to the nature of the prediction scores, typically a threshold of 0.5 is used to determine if a compound is predicted to be active, but higher scores suggest a higher likelihood of activity. Applicability scores are based on the reliability-density neighborhood (RDN) method which considers both the model overlap and the individual bias and precision of the overlapping fingerprints. There is not a defined threshold for an acceptable applicability score, but it is ideal to have a higher score for confidence in the prediction (1 max). The consensus scores are based on majority rule classification (agreement for ≥ 4 algorithms, when equal to 4 a compound is predicted as active). The threshold for activity is shown for each model........S28

Table S6. Predicted *in vitro* ADME properties for 5a (12126072). Highlighted has also been determined experimentally. Due to the nature of the prediction scores, typically a threshold of 0.5 is used to determine if a compound is predicted to be active, but higher scores suggest a higher likelihood of activity. Applicability scores are based on the reliability-density neighborhood (RDN) method which considers both the model overlap and the individual bias and precision of the overlapping fingerprints. There is not a defined threshold for an acceptable applicability score, but it is ideal to have a higher score for confidence in the prediction (1 max). The consensus scores are based on majority rule classification (agreement for ≥ 4 algorithms, when equal to 4 a compound is predicted as active). The threshold for activity is shown for each model. S29

Table S7. *In vitro* ADME properties for compounds 2d, 2c, and 5a. S30

List of Supplementary Figures

Figure S1. Hemolytic activity of test compounds at 10 µM on human red blood cells after 72 h. Hemolysis was quantified by measuring hemoglobin release at 540 nm and expressed as a percentage relative to untreated (negative control) and 0.1% saponin-treated (positive control) RBCs. DMSO at the highest concentration used served as the vehicle control........S31

Figure S2. Isobolograms of association of compound **1b** (A), **2a** (B), **2b** (C) and quinacrine (D) with proguanil. Experimental data are represented by the red region and red dots, while the black line and gray region indicate the additivity curve. Statistical analysis for the combination of **1b** (**E**), **2a** (**F**), **2b** (**G**) and quinacrine (**H**) with proguanil. The data represent the ∑FIC50 values from three independent experiments. Statistical analysis was conducted by using Student's paired t-test (p-value < 0.027 indicates a statistical distinction between the experimental findings and the additivity isobole)........S32

Figure S3. In vivo anti-malarial activity assessment and survival analysis in mice infected with *P. berghei* NK65. Upper panels: Parasitemia reduction following treatment with pyronaridine **1a** (**A**) and compound **1d** (**B**) at 50 mg/kg/day for three consecutive days post-infection is shown relative to the untreated control group. Chloroquine (20 mg/kg/day) was included as a reference antimalarial. Bottom panels: Survival analysis of mice treated (PO, 50 mg/kg/day) with compound pyronaridine **1a** (**A**) and **1d** (**B**) and standard antimalarial chloroquine evaluated during the experiment (30 days) (n = 3–4)........S32

Figure S4. ^{1}H NMR spectrum (200 MHz, DMSO-d_6) of compound **4a**........S33

Figure S5. ^{13}C NMR spectrum (50 MHz, DMSO-d_6) of compound **4a**........S33

Figure S6. ^{1}H NMR spectrum (200 MHz, DMSO-d$_6$) of compound **4b**……………………………...S34
Figure S7. ^{13}C NMR spectrum (50 MHz, DMSO-d$_6$) of compound **4b**……………………………S34
Figure S8. ^{1}H NMR spectrum (200 MHz, DMSO-d$_6$) of compound **4c**……………………………S35
Figure S9. ^{13}C NMR spectrum (50 MHz, DMSO-d$_6$) of compound **4c**……………………………S36
Figure S10. ^{1}H NMR spectrum (200 MHz, DMSO-d$_6$) of compound **4d**………..……………….…..S36
Figure S11. ^{13}C NMR spectrum (50 MHz, DMSO-d$_6$) of compound **4d**..……………………...…S36
Figure S12. ^{1}H NMR spectrum (200 MHz, DMSO-d$_6$) of compound **5a**..…………………………..S37
Figure S13. ^{13}C NMR spectrum (50 MHz, DMSO-d$_6$) of compound **5a**..…………………………..S37
Figure S14. ^{1}H NMR spectrum (200 MHz, DMSO-d$_6$) of compound **5b**..………………….……S38
Figure S15. ^{13}C NMR spectrum (50 MHz, DMSO-d$_6$) of compound **5b**..…………………...…S38
Figure S16. ^{1}H NMR spectrum (200 MHz, DMSO-d$_6$) of compound **6**..…………………………S39
Figure S17. ^{13}C NMR spectrum (50 MHz, DMSO-d$_6$) of compound **6**..…………………………S39
Figure S18. ^{1}H NMR spectrum (200 MHz, DMSO-d$_6$) of compound **7**..…………………………S40
Figure S19. ^{13}C NMR spectrum (50 MHz, DMSO-d$_6$) of compound **7**..…………………………S40
Figure S20. HR-MS data for compound **4a**..……………………………………..…………………S41
Figure S21. HR-MS data for compound **4b**..………………………………………………………S42
Figure S22. HR-MS data for compound **4c**……...………………………..…………………………S43
Figure S23. HR-MS data for compound **4d**..………...……………………..…………………………S44
Figure S24. HR-MS data for compound **5a**………………………………..…...………………………S45
Figure S25. HR-MS data for compound **5b**……...…………………………..…………………………S46
Figure S26. HR-MS data for compound **6**……...……...…………………..…………………………S47
Figure S27. HR-MS data for compound **7**………....……………………..…………………………S48
Figure S28. HPLC trace of the compound **4a**……...………………….………………………………S49
Figure S29. HPLC trace of the compound**4b**……...………………….………………………………S49
Figure S30. HPLC trace of the compound **4c**……...………………….………………………………S50
Figure S31. HPLC trace of the compound **4d**……...………………….………………………………S50
Figure S32. HPLC trace of the compound **5a**……...………………….………………………………S51
Figure S33. HPLC trace of the compound **5b**……...………………….………………………………S51
Figure S34. HPLC trace of the compound **6**……...……………………………………………………S52
Figure S35. HPLC trace of the compound **7**……...………………….…...……………………………S52
SI References...S53

METHODS

Chemistry

All reagents and solvents were purchased from commercial suppliers (AlfaAesar, Acros, Chimmed) and used without further purification. The ^{1}H and ^{13}C spectra were recorded on a Bruker AC-200 (200 MHz, ^{1}H; 50 MHz, ^{13}C) NMR spectrometer. Chemical shifts were measured in DMSO-d_6 or $CDCl_3$ and reported as ppm values. Low-resolution mass spectra were recorded on a Finnigan MAT INCOS 50 quadrupole mass spectrometer (EI, 70 eV) with direct injection. High-resolution mass spectra were recorded on an Impact II QqTOF high-resolution mass-spectrometer (Bruker Daltonik, Germany) equipped with an Apollo II ESI ion source (Bruker Daltonik) according to the following conditions: direct sample infusion at 0.25 mL/min, ion source in positive mode, high voltage (HV) capillary at 4.5 kV, spray gas – nitrogen at 2.5 bar, dry gas – nitrogen at 6 L/min 220°C, scan range m/z 50-1500, 3 Hz scan rate, automatic internal calibration with sodium trifluoroacetate solution. Spectra were processed with Compass DataAnalysis 5.1 (Bruker Daltonik). The purity of the final compounds was analyzed on an Agilent 1290 Infinity II HPLC system coupled to an Agilent 6460 triple-quadrupole mass spectrometer equipped with an electrospray ionization source. Chromatographic separation was performed on an Agilent Eclipse Plus C18 RRHD column (2.1 × 50 mm, 1.8 μm) at 40 ºC; sample injection volume – 0.2 μL. A mobile phase consisting of 0.1 % formic acid/water (A), and 0.1 % formic acid and 85 % acetonitrile/water (B) was programmed with gradient elution at a flow rate of 0.4 mL/min as follows: 0.0-3.0 min, 60 % B; 3.0-4.0 min, 60 % to 97 % B; 4.0-6.0 min, 97 % B; 6.0-6.1 min, 97 % to 60 % B. Mass spectrometric detection was operated in the positive ion mode. The optimal parameters were: capillary voltage 3500 V, nebulizer pressure 35 psi, gas temperature 350 ºC, gas flow rate 12 L/min. Melting points were determined on an Electrothermal 9001 melting point apparatus (10 °C per min) and were uncorrected. Merck KGaA silica gel 60 F_{254} plates were used for analytical thin-layer chromatography. Spots were detected by an ultraviolet lamp. All final compounds were ≥ 98 % pure.

General procedure for the synthesis of acridines **4a-d:** Triethylamine hydrochloride (0.8 equiv.) was added portion-wise to a suspension of corresponding benzoic acid (1 equiv.) in phosphorus oxychloride (3 mL), and the resulting mixture was stirred at reflux for 5-6 h. Then the mixture was cooled and poured on ice. After 30-min stirring ammonia solution was added dropwise to pH 8, the precipitate of chloro-acridine was filtered, washed with ethanol and n-hexane to afford the desired product.

N1,N1-Diethylpentane-1,4-diamine (6 equiv.) was added to a suspension of chloro-acridine (1 equiv.) in dry DMF (7 mL), and the reaction mixture was stirred at 100 °C for 6 h. Then the solution was cooled, diluted with waster and extracted with ethyl acetate (3x30 mL). The organic layer was acidified by an aqueous solution of hydrochloric acid to pH 2, and the aqueous layer was separated. The organic layer was additionally washed with water until the aqueous layer became pH 7. The combined aqueous solution was evaporated in vacuo. A mixture of ethyl acetate:isopropanol (5:1) was added to the residue and the resulting mixture was boiled until crystals appeared. The precipitate of aim acridine was filtered, washed with isopropanol, ethyl acetate and ether to afford the desired product.

N^4-(2-Chloro-7-methoxyacridin-9-yl)-N^1,N^1-diethylpentane-1,4-diamine hydrochloride **4a**

Yield 56 %, mp. 232-236 °C. ^{1}H NMR (200 MHz; DMSO-d_6; δ, ppm; *J*, Hz): 1.10 (t, 6H, 2CH_3, *J* = 6.7), 1.65 (d, 5H, $C\underline{H}_3CHC\underline{H}_2$, *J* = 6.0), 1.88 and 2.11 (s and s, 1H and 1H, $C\underline{H}_2CH_2N$), 2.93 (s, 2H, $C\underline{H}_2N$), 3.36 (s, 4H, 2$C\underline{H}_2$-CH_3), 4.00 (s, 3H, OCH_3), 4.59 (brs, 1H, CH), 7.71 (d, 1H, HC(6), *J* = 9.2), 8.03 (dt, 4H, HC(3,4,5,8), *J* = 18.0, 9.5), 8.64 (s, 1H, HC(1)), 9.55 (brs, 1H, NH), 10.44 (s, 1H, NH), 14.93 (s, 1H, OH). ^{13}C NMR (50 MHz; DMSO-d_6; δ, ppm): 8.17, 19.96, 20.77, 33.64, 49.80, 55.30, 56.22, 104.12, 112.79, 113.36, 120.66, 120.98, 124.04, 127.45, 127.62, 134.28, 135.35, 137.54, 155.48. MS (EI): m/z 399. Anal. calcd for $C_{23}H_{30}ClN_3O$: C, 69.07; H, 7.56; N, 10.51. Found: C, 69.11; H, 7.59; N, 10.48.

N4-(2-chloro-7-methoxy-9-acridinyl)-N1,N1-diethyl-1,4-pentanediamine hydrochloride **4b**

Yield 42 %, mp. 221-223 °C. ^{1}H NMR (200 MHz, D_2O, 303K δ, ppm; *J*, Hz): 1.08 (t, *J* = 7.2 Hz, 3H, $NCH_2C\underline{H}_3$), 1.10 (t, *J* = 7.4 Hz, 3H, $NCH_2C\underline{H}_3$), 1.50 (q, *J* = 10.7, 9.1 Hz, 2H, $H_2C(3)$), 1.64 (d, *J* = 6.3 Hz, 1H, $H_3CC(4)$), 2.02 – 1.74 (m, 2H, H2C(2)), 3.10 – 2.88 (m, 6H, H_2CN), 3.98 (s, 3H, H_3CO), 4.60 – 4.33 (m, 1H, HC(4)), 7.64 – 7.33 (m, 5H, $HC_{Ar}(1,3,5,6,8)$, 7.99 (d, *J* = 9.2 Hz, 1H, HC(4)). ^{13}C NMR (50 MHz, D_2O, 303K δ, ppm): δ 157.42 ($C_{Ar}(7)$), 142.06 ($C_{Ar}(9)$), 140.34 ($C_{Ar}(4a)$), 135.42 ($C_{Ar}(10a)$), 128.75 ($C_{Ar}(3)$), 128.19 ($C_{Ar}(2)$), 125.96 ($C_{Ar}(4,5)$), 121.62($C_{Ar}(6)$), 118.57 ($C_{Ar}(1)$), 114.73 ($C_{Ar}(9a)$), 110.82 ($C_{Ar}(8a)$), 103.95 ($C_{Ar}(8)$), 57.56 (CH_3O), 56.86 ($H_2C(1)N$), 52.12 (C(4)), 48.61 and 48.53 ($N\underline{C}H_2CH_3$), 35.12 (C(3)), 21.75 (C(2,5)), 9.41 ($NCH_2\underline{C}H_3$). MS (EI): m/z 399. Anal. calcd for $C_{23}H_{30}ClN_3O$: C, 69.07; H, 7.56; N, 10.51. Found: C, 69.18; H, 7.51; N, 10.55.

N^1,*N*^{1}-Diethyl-*N*^{4}-(2-methoxy-7-(trifluoromethyl)acridin-9-yl)pentane-1,4-diamine hydrochloride **4c**

Yield 70 %, mp. 212 °C (decomp.). ^{1}H NMR (200 MHz; DMSO-d_6; δ, ppm; *J*, Hz): 1.12 (t, 6H, $2CH_3$, *J* = 6.7), 1.70 (d, 5H, $C\underline{H_3}CHC\underline{H_2}$, *J* = 6.1), 1.93 and 2.14 (s and s, 1H and 1H, $C\underline{H_2}CH_2N$), 2.94 (s, 2H, $C\underline{H_2}N$), 3.38 (s, 4H, $2C\underline{H_2}$-CH_3), 4.02 (s, 3H, OCH_3), 4.57 (brs, 1H, CH), 7.87 (dd, 1H, HC(3)), 7.94-8.37 (m, 4H, HC(4,5,6,8)), 8.83 (brs, 1H, HC(1)), 10.08 (s, 1H, NH), 10.49 (s, 1H, NH), 15.03 (s, 1H, NH). ^{13}C NMR (50 MHz; DMSO-d_6; δ, ppm): 8.12, 8.17, 19.90, 20.58, 33.46, 45.80, 49.72, 55.73, 56.45, 104.48, 110.46, 114.04, 120.33, 120.63, 121.24, 121.68, 122.28, 122.93, 124.17, 126.64, 127.54, 129.36, 132.07, 135.30, 140.72, 155.85, 156.95. MS (EI): m/z 433. Anal. calcd for $C_{24}H_{30}F_3N_3O$: C, 66.49; H, 6.98; N, 9.69. Found: C, 66.54; H, 7.02; N, 9.73.

N^4-(2,7-Dichloroacridin-9-yl)-*N*^{1},*N*^{1}-diethylpentane-1,4-diamine hydrochloride **4d**

Yield 61 %, mp. 256-258 °C. ^{1}H NMR (200 MHz; DMSO-d_6; δ, ppm; *J*, Hz): 1.15 (t, 6H, $2CH_3$, *J* = 6.7), 1.63 (d, 5H, $C\underline{H_3}CHC\underline{H_2}$, *J* = 5.7), 1.94 and 2.12 (s and s, 1H and 1H, $C\underline{H_2}CH_2N$), 2.98 (s, 2H, $C\underline{H_2}N$), 3.26 (brs, 4H, $N(C\underline{H_2}CH_3)_2$), 4.56 (brs, 1H, CH), 7.84-8.28 (m, 4H, HC(3,4,5,6)), 8.67 (brs, 2H, HC(1,8)), 9.84 (brs, 1H, NH), 10.56 (brs, 1H, NH), 15.02 (brs, 1H, NH). ^{13}C NMR (50 MHz; DMSO-d_6; δ, ppm): 8.27, 19.92, 20.43, 33.42, 46.11, 50.11, 55.24, 113.25, 121.15, 124.64, 127.97, 135.02, 138.75, 155.77. MS (EI): m/z 404. Anal. calcd for $C_{21}H_{26}Cl_2N_3$: C, 65.35; H, 6.73; N, 10.39. Found: C, 65.38; H, 6.78; N, 10.34.

Compound 6-chloro-2-methoxy-9(10H)-acridinone **5a** were synthesized according Šafařík et al.,[1] and its original analytical data presented here.

6-chloro-2-methoxy-9(10H)-acridinone **5a**

Yield 64%, mp >330 °C (decomp.). ^{1}H NMR (200 MHz; DMSO-d_6; δ, ppm; J, Hz): 12.23 (s, 1H, H-10), 8.21 (d, 1H, J = 8.7, H-8), 7.63 (d, 1H, J = 2.0, H-5), 7.60 (d, 1H, J = 3.0, H-1), 7.58 (d, 1H, J = 8.9, H-4), 7.42 (dd, 1H, J = 9.0, J = 3.0, H-3), 7.22 (dd, 1H, J = 8.7, J = 2.0, H-7), 3.85 (s, 3H, OCH_3). ^{13}C NMR (50 MHz; DMSO-d_6; δ, ppm): 175.8 (C-8), 154.5 (C-2), 141.4 (C-4b), 137.8 (C-6), 135.9 (C-4a), 128.5 (C-8), 124.7 (C-3), 121.4 (C-8b), 121.3 (C-7), 119.6 (C-4), 118.4 (C-8a), 116.5 (C-5), 105.2 (C-1), 55.6 (OCH_3). MS (EI): m/z: 259. Anal. calcd for $C_{14}H_{10}ClNO_2$: C, 64.75; H, 3.88; N, 5.39. Found: C, 64.86; H, 4.01; N, 5.37.

Compounds 3-morpholin-4-yl-10-oxo-5,10-dihydrobenzo[b]-1,6-naphthyridine-4-carbonitrile 5b, 3-Morpholin-4-ylbenzo[b]-1,6-naphthyridine-4-carbonitrile 6 and 6-chloro-2-methoxy-9-morpholin-4-ylacridine 7 were synthesized according Puhl et al.,[2] and their original analytical data presented here.

3-Morpholin-4-yl-10-oxo-5,10-dihydrobenzo[b]-1,6-naphthyridine-4-carbonitrile 5b

Yield 38%. 1H NMR (200 MHz; DMSO-d6; δ, ppm; J, Hz): 8.02 (1H, s, CH), 7.91 (1H, d, J = 6.5, CH), 7.59 (1H, s, CH), 7.09 (1H, d, J = 6.5, CH), 4.08 (4H, m, O(CH2)2), 3.72 (4H, m, N(CH2)2). 13C NMR (50 MHz; DMSO-d6; δ, ppm): 182.5, 146.4, 146.1, 139.7, 139.1, 138.7, 137.0, 124.9, 122.7, 118.3, 115.4, 108.8, 94.9, 66.8, 53.7. MS (EI): m/z: 306. Anal. calcd for C17H14N4O2: C, 66.66; H, 4.61; N, 18.29. Found: C, 66.75; H, 4.54; N, 18.13.

3-Morpholin-4-yl-10-oxo-5,10-dihydrobenzo[b]-1,6-naphthyridine-4-carbonitrile **5b**

Yield 38%. ^{1}H NMR (200 MHz; DMSO-d_6; δ, ppm; *J*, Hz): 8.02 (1H, s, CH), 7.91 (1H, d, J = 6.5, CH), 7.59 (1H, s, CH), 7.09 (1H, d, J = 6.5, CH), 4.08 (4H, m, $O(CH_2)_2$), 3.72 (4H, m, $N(CH_2)_2$). ^{13}C NMR (50 MHz; DMSO-d_6; δ, ppm): 182.5, 146.4, 146.1, 139.7, 139.1, 138.7, 137.0, 124.9, 122.7, 118.3, 115.4, 108.8, 94.9, 66.8, 53.7. MS (EI): m/z: 306. Anal. calcd for $C_{17}H_{14}N_4O_2$: C, 66.66; H, 4.61; N, 18.29. Found: C, 66.75; H, 4.54; N, 18.13.

3-Morpholin-4-ylbenzo[b]-1,6-naphthyridine-4-carbonitrile **6**

Yield 78%, mp 222−225 ºC (EtOH:DMF 2:1). ^{1}H NMR (200 MHz; DMSO-d_6; δ, ppm; *J*, Hz): 9.46 (1H, s, CH), 9.13 (1H, s, CH), 8.16 (1H, d, J = 6.5, CH), 7.91 (1H, s, CH), 7.44 (1H, d, J = 6.5, CH), 4.16 (4H, m, $O(CH_2)_2$), 3.78 (4H, m, $N(CH_2)_2$). ^{13}C NMR (50 MHz; DMSO-d_6; δ, ppm): 144.9, 143.5, 137.8, 136.4, 136.0, 133.1, 129.5, 128.4, 126.8, 126.1, 114.5, 101.4, 66.7, 53.8. MS (EI): m/z 290. Anal. calcd for $C_{17}H_{14}N_4O$: C, 70.33; H, 4.86; N, 19.30. Found: C, 70.39; H, 4.02; N, 19.68.

6-chloro-2-methoxy-9-morpholin-4-ylacridine **7**.

Yield 70%, mp 208-212 ºC (DMF). ^{1}H NMR (200 MHz; DMSO-d_6; δ, ppm; *J*, Hz): 8.56 (1H, d, J = 9.0, CH), 7.72 (H, s, CH), 7.62 (1H, d, J = 4.5, CH), 7.44 (1H, d, J = 4.5, CH), 7.27 (1H, s, CH), 7.07 (1H, d, J = 9.0, CH), 3.86 (3H, s, OCH_3). ^{13}C NMR (50 MHz; DMSO-d_6; δ, ppm): 155.7, 149.8, 142.6, 134.5, 131.7, 130.9, 130.6, 127.6, 122.4, 119.7, 119.1, 114.8, 100.5, 66.8, 55.7, 50.8. MS (EI): m/z 328. Anal. calcd for $C_{18}H_{17}ClN_2O_2$: C, 65.75; H, 5.21; N, 8.52. Found: C, 65.87; H, 5.16; N, 8.64.

Biology

Hemolysis Assay

The potential hemolytic effects of the test compounds were evaluated using freshly collected human red blood cells (RBCs). Each compound was incubated at a final concentration of 10 µM with RBCs adjusted to a 2% hematocrit in 96-well plates and maintained at 37 °C. Hemolysis was assessed after 72 hours of incubation. Three control conditions were included: untreated RBCs in RPMI medium (negative control), RBCs exposed to 0.1% saponin (positive control), and RBCs treated with the highest DMSO concentration present in the test wells (vehicle control).

At each time point, plates were centrifuged, and the supernatants were transferred to a fresh plate for quantification of hemoglobin release by measuring absorbance at 540 nm. The percentage of hemolysis was calculated using the formula:

$$\text{Hemolysis (\%)} = 100 \times \frac{A_{\text{sample}} - A_{\text{negative control}}}{A_{\text{positive control}} - A_{\text{negative control}}}$$

where A_{sample}represents the absorbance of the test sample, $A_{\text{negative control}}$corresponds to RBCs incubated with RPMI medium, and $A_{\text{positive control}}$refers to RBCs treated with 0.1% saponin, which was defined as 100% hemolysis.

In vitro antiplasmodial blood stage activity

The antiplasmodial activity of the AC-based compounds was assessed against *P. falciparum* blood parasites 3D7 strain, known for its chloroquine sensitivity. Parasites were synchronized to the ring stage through sorbitol treatment as described by Lambros and Vanderberg (1979).[3] Cultures with 0.5% parasitemia and 2.0% hematocrit were dispensed into 96-well plates and treated with serially diluted compounds. Uninfected and infected erythrocyte cultures without treatment served as negative and positive controls, respectively, maintained in parallel. DMSO concentration was kept below 0.05% (v/v). Plates were incubated for 72 hours at 37°C in a humidified incubator with a gas mixture

containing 90% N_2, 5% O_2, and 5% CO_2. Once the incubation period was completed, the density of parasites was determined using the SYBR Green I method.[4] The half-maximal inhibitory concentration (IC_{50}) was determined by non-linear regression analysis of the concentration-response curve using the GraphPad Prism 8 program (GraphPad Software, San Diego, California, USA). The study was approved by the Research Ethics Committee (CAAE 67642722.50000.5505). Peripheral blood was obtained from healthy adult volunteers after written informed consent.

Cross-resistance studies

The antiplasmodial activity of test compounds was evaluated against a representative panel of *P. falciparum* resistant strains. The panel included: 3D7 (chloroquine-sensitive), Dd2 and K1 (resistant to chloroquine, pyrimethamine, and sulfadoxine), $3D7^{R}$_MMV848 (resistant to MMV692848, a PfPI4K inhibitor) and TM90C6B (resistant to atovaquone). The evaluation of the IC_{50} values of the compounds against each of the panels of resistant strains was conducted as previously described above. After the determination of the IC_{50} value for each resistant strain, a resistance index (RI) was calculated by the ratio of $IC_{50}^{\text{Resistant strain}}$ to IC_{50}^{3D7}. Of note, RI values greater than 5 were considered indicative of cross-resistance.[5]

***Ex vivo* clinical isolate schizont maturation assay**

The study received ethical approval from the Centro de Pesquisa em Medicina Tropical (CEPEM–Rondônia) ethics committee (CAAE 58738416.1.0000.0011). All volunteers provided written informed consent prior to blood collection, which was performed by a trained nurse. Clinical isolates of *P. falciparum* and *P. vivax* were obtained in September and October 2024 from patients enrolled at the Centre of Malaria Control (CEPEM) in Porto Velho, Rondônia, located in the western Brazilian Amazon. Only individuals with confirmed *P. falciparum* monoinfections, parasite densities ranging from 2,000 to 80,000 parasites/µL, and at least 70% ring-stage parasites were eligible. Participants who

had taken antimalarial medication within the previous month or who showed signs of severe malaria were excluded. For each participant, a 5-mL peripheral venous blood sample was drawn into heparinized tubes and immediately used for ex vivo drug susceptibility testing with pre-dosed plates containing serial dilutions of antimalarial compounds.[6]

Test compounds, along with standard antimalarials such as artesunate and chloroquine, were assessed on 7 *P. vivax* and 6 *P. falciparum* isolates, all of which were subjected to compound incubation for ≥ 40 hours. IC_{50} values, median IC_{50} values, and scatter plots were generated using GraphPad 8.0.1 (GraphPad Prism, San Diego, California, USA).

Speed-of-Action assay

To determine whether test compounds acted as fast- or slow-acting inhibitors, a protocol adapted from Le Manach et al.,[7] was used. In this method, 180 µL of synchronized ring-stage 3D7 culture at 0.5% parasitemia and 2% hematocrit were added in three identical 96-well plates containing 20 µL of 10-fold concentrated compounds at a range of concentrations obtained by serial 2-fold dilution. DMSO concentration was maintained below 0.05% (v/v). The positive and negative controls consisted of parasite cultures with no addition of compounds and non-parasitized erythrocytes, respectively. Each plate was incubated with the compounds for a different time (24, 48, or 72h), and the first two plates, the 24h and 48h plates, were washed twice (centrifugation: 2 min, 150 × g) with fresh medium to remove the compounds, followed by incubation for 48 and 24h, respectively. The IC_{50} of each incubation time was determined at the end of 72 hours by the SYBR Green I method. The resulting IC_{50} values were compared using ANOVA to determine statistical significance of differences. In parallel, the parasite's morphological progression under compound exposure was examined by treating cultures with the highest concentration of each compound for 24 hours. After this period, the plate was washed twice with fresh medium to remove the inhibitor. Blood smears were then prepared and stained

from each well at 24, 48, and 72 hours. For growth-control purposes, blood smears from a positive control culture, maintained without any inhibitor, were collected at 0, 24, 48, and 72 hours.

Compounds that demonstrated fast-acting activity within 24 h, along with the reference drugs artesunate and chloroquine, were further evaluated using short-exposure assays. Parasite cultures were incubated with each compound at the tested concentration for exposure periods of 3 h, 6 h, 9 h, and 12 h. At the end of each exposure period, cultures were washed twice with fresh complete medium to remove residual compound and then incubated under drug-free conditions until a total assay duration of 72 h was reached. Parasite growth was subsequently quantified as described above. For each compound, the IC_{50} value obtained after the short exposure was compared with the standard 72 h IC_{50}, and an IC_{50} ratio (short-exposure IC_{50} / 72 h IC_{50}) was calculated to assess the impact of reduced exposure time on drug activity.

Activity in combination with proguanil

Drug combination assays were conducted following the methodology outlined by Fivelman et al.[8] The compounds under investigation and standard antimalarials were diluted and mixed in fixed ratios within a 96-well plate, spanning seven combinations (1:0, 6:1, 5:2, 4:3, 3:4, 2:5, 1:6, 0:1). Initial concentrations were set at 10 times the IC_{50} for all compounds, with experiments conducted at 0.5% parasitemia and 2% hematocrit. Serial dilutions of these mixtures were prepared and co-incubated with the parasite to assess antiplasmodial activity against *P. falciparum*, employing the SYBR Green I method. IC_{50} values for each combination were determined using GraphPad Prism version 8.0.1 (GraphPad Software, San Diego, CA, USA). Additivity was assessed using the Hand model,[9] with Fractional Inhibitory Concentration (FIC_{50}) values calculated for seven different compound proportions, expressed in terms of IC_{50} equivalents. FIC_{50} values from three independent experiments were subjected to non-linear fitting and statistically compared to the additivity isobole. Absence of a statistical difference between the model and the additivity isobole signified an additive drug

combination, while distinct curves indicated synergy (model below the additivity curve) or antagonism (model above the additivity curve).

Intracellular localization studies by confocal microscopy

Erythrocytes infected with *P. falciparum* (3D7 strain) nonsynchronous parasites were washed in MOPS buffer (116 mM NaCl, 5.4 mM KCl, 0.8 mM $MgSO_4$, 5.5 mM D-glucose, 50 mM MOPS, and 2 mM $CaCl_2$, pH 7.4), resuspended in the same buffer, and plated on a microscopy chamber previously pre-treated for 1 h with L-polylysine (1 mg/mL). The localization of the autofluorescent AC-based compound **2d** was achieved by adding 10 μM of it to the cells immobilized in the chamber. Additionally, the tetramethyl rhodamine ethyl ester (TMRE) was used to assess whether the autofluorescent AC-based compound **5a** affected the membrane potential of mitochondria in malaria-infected red blood cells. The cells immobilized in the chamber were loaded with 100 nM of TMRE, and subsequently, compound **5a** (10 μM) was added. Membrane potential-dependent fluorescence changes were then monitored in real-time. Fluorescence signals from compound **2d**, TMRE, **5a**, and in malaria-infected erythrocytes were captured using a confocal microscope (Leica TCS SP8) with the following parameters: λ_{EX} 488 nm/λ_{EM} 490-525 nm for TMRE and λ_{EX} 405 nm/λ_{EM} 420-470 nm for **2d** and **5a.** Fluorescence quantification of compound **2d** both infected and uninfected erythrocytes was performed by selecting regions of interest (ROIs) using Leica LASX software. Statistical analysis was carried out with GraphPad Prism version 8.0.1. Significant differences were determined using Student's t-test (****$p \leq 0.0005$).

In vivo assay against *Plasmodium berghei*

A suppressive parasite growth test was performed in mice infected with *P. berghei* NK65 strain (originally received from the New York University Medical School), as described previously,[10] with some modifications. Briefly, adult female Swiss outbred mice (20 ± 2 g weight) were intraperitoneally

inoculated with 1×10^5 red blood cells infected with *P. berghei*. The infected mice were maintained together for at least 2 h and then randomized into groups of 3 or 4 animals per cage, which were subsequently administered 50 mg/kg/day of each compound diluted in 3% (v/v) DMSO by oral gavage daily for 3 days. Two control groups were used in parallel: one was treated with CQ (20 mg/kg/day), and the other was treated with the vehicle, both for 3 days. Blood smears from mouse tail vein were prepared on days 5, 8, and 11 of the experiment (total of 30 days of experiment) and then fixed with methanol, stained with Giemsa 10% (v/v), and examined under the microscope. The survival of the mice was carefully monitored throughout the entire duration of the experiment, extending up to the thirtieth day. Parasitemia was evaluated, and the percent inhibition of parasite growth was calculated in relation to the untreated group (considered 100% growth) using the following equation: (C – T/C) x 100; where C is the parasitemia in the control group and T is the parasitemia in the treated group. In the *P. berghei* study, after day 11, animals were monitored daily and classified according to pre-established humane endpoint criteria. These criteria included: changes in body weight and food/water intake; external physical appearance (ungroomed and ruffled fur, closed eyelids, ocular/nasal discharge, hunched posture, etc.); clinical signs (alterations in heart and respiratory rates, dyspnea, changes in defecation, body temperature, etc.); unprovoked behavioral changes (e.g., vocalization, self-injury); and behavioral responses to external stimuli (e.g., aggressiveness).[11] All institutional and national guidelines for the care and use of laboratory animals were followed. The use of laboratory animals was approved by the Ethics Committee for Animal Use of Universidade Federal do Estado de São Paulo, UNIFESP (CEUA N 6630080816)

Inhibition cytochrome bc1 complex assay

P. falciparum mitochondria were extracted from isolated parasites according to Okada-Junior,[12] with some modifications. Briefly, infected red blood cells were lysed with saponin (0.05% w/v solution) and washed with PBS. The parasite was subjected to disruption by nitrogen cavitation (400 psi for 30

min), followed by centrifugation (16,000 g, 4 °C, 1h). The pellet was resuspended in 2% DDM in 50 mM potassium phosphate buffer pH 7.4 for 1 hour (0.5 mL of buffer/mL of parasite original pellet). The mitochondrial fraction was obtained after centrifugation (16,000 g, 4°C, 1h), and the total amount of protein in the mitochondrial fraction obtained was then quantified using the BCA Pierce assay. The enzymatic assay consisted of 50 mM phosphate buffer (pH 7.4), 2 mM EDTA, 1 mM NaN_3, 0.03% DDM, and 75 μM cytochrome c (C7752, Sigma Aldrich), and the amount of purified mitochondria solution for assays was determined after each extraction by optimization of signal-to-noise ratios obtained from each batch (between 150 and 200 μg/mL of total proteins from the mitochondrial fraction was enough). The reaction was started by the addition of 100 μM of decylubiquinol, and cytochrome bc1 activity was quantified by indirectly monitoring cytochrome c reduction through the increase in absorbance at 550 nm vs. 540 nm. Percent inhibition was quantified as a decrease in the slope of the resulting curves compared to the positive (2 μM of atovaquone) and negative (50 mM potassium phosphate buffer pH 7.4) control of inhibition.

Beta-hematin inhibition Assay

The β-hematin inhibition assay was performed as previously described.[13] Stock solutions of chloroquine, pyronaridine, and compounds **2d** and **5a** were initially prepared at 20 mM in DMSO. Each of these stocks was subsequently diluted ten-fold in a buffer containing NP-40 detergent, giving a working solution composed of 61.1 μM NP-40 and 10% DMSO in a final volume of 100 μL. From these working solutions, a series of compound dilutions was generated using the same detergent-containing buffer. To prepare hemin, a 25 mM solution was produced by sonicating the pigment in DMSO for one minute. A volume of 179 μL of this solution was then mixed with 20 mL of 1 M acetate buffer (pH 4.8), yielding a uniform suspension. This suspension was dispensed (100 μL) into assay wells so that the final mixture in each well contained 0.5 M acetate buffer and 100 μM hemin. Plates were sealed and kept at 37 °C for 5 hours to allow β-hematin formation.

A detection solution consisting of pyridine, water, acetone, and 2 M HEPES (pH 7.4) in a 50:20:20:10 (v/v/v) ratio was prepared. From this mixture, 32 μL was added to each well, resulting in a final pyridine concentration of 6.9% (v/v). An additional 60 μL of acetone was then introduced to facilitate dispersal of the β-hematin crystals. Absorbance readings were obtained using a SpectraMAX Plus 384 plate reader (Molecular Devices Corp., Sunnyvale, CA). Dose–response curves were generated in GraphPad Prism, and IC_{50} values for each test compound we determined from these fitted models.

Selecting for resistance in *P. falciparum in vitro*.

The generation of resistance parasites was performed according to Paquet et al.,[14] with some modifications. Dd2 DNA polymerase δ mutant (Dd2-Polδ) parasites were maintained in complete culture media at 5% hematocrit and under the gas mixture (90% N_2, 5% CO_2, 5% O_2) at 37°C. On day 0, three independent flasks, each containing an inoculum of $1x10^9$ parasites, were maintained in the presence of compound **5a** and pyronaridine (**1a**) at $3xIC_{90}$, which was determined by using the parental line. Microscopic examination of Giemsa-stained thin blood smears was used to monitor parasitemia. Culture media was changed daily, with compound reposition, until parasite clearance, and every 3 to 4 days for the remainder of the experiment. The flasks with recrudescent parasites were subjected to IC_{50} determination to assess resistance. Resistant clones were isolated by limiting dilution in 96-well plates in compound-free RPMI.

Genomic DNA was extracted from saponin isolated parasites using the DNA Preparation Kit (Cellco). Next, fragmentation and DNA tagging were performed using the Illumina DNA Prep (M) Tagmentation kit, and the library constructed using Illumina DNA/RNA UD indexes Set A (96 indexes, 96 samples). Whole-genome sequencing was performed on an Illumina Nextseq 1000/2000 sequencing platform. Finally, sequences were aligned to the Dd2-Polδ parental line genome, and variants were identified using a previously established analysis pipeline to obtain 300 bp paired-end reads at an average of 30× depth of coverage.

Sanger sequencing

The amplification reactions (Polymerase Chain Reactions, PCRs) were performed using the PCRBIO Taq Mix kit (PCR Biosystems™). The following primers were used to sequence T-complex protein 1 (TCP-1) subunit epsilon (PfDd2_030025400), Glutathione synthetase (GS) (PfDd2_050017100) and lipase putative (PfDd2_140031600): TCP-1(F) – TATCAGAGCTTTTGGTAATGC, TCP-1(R) – AATTGAAGTCGTTTGGAGC; GS (F) – ATGGAAAGAAAGGTAGATGAG, GS (R) – CTTTATGTGAACCTAATAAAGGC; lipase (F) – GTAAAAGCGTCTTTAACGC, lipase (R) – CATCACAATTTTCATTATTCGG.

Thermal conditions included initial denaturation at 95°C for 3 minutes, followed by 35 cycles of denaturation at 95°C for 30 seconds, annealing at 46°C for 30 seconds, and extension at 60°C for 2 minutes, ending with a final extension at 65°C for 5 minutes.

For the sequencing reaction (SR), the purified fragments were processed using the BigDye Terminator Cycle Sequencing Ready Reaction, version 3.1. Sequencing reactions were performed according to the manufacturer's recommendations. The sequencing reaction products were purified with ethanol and analyzed using the ABI Prism 3730 DNA Analyzer (Applied Biosystems™) to identify SNPs (single nucleotide polymorphisms). The sequences, both forward and reverse, were examined using the BioEdit Sequence Alignment Editor software (version 7.2.5), and only those with confidence indices above 99% were considered for analysis.

***P. falciparum* Stage-Specificity Assay**

To identify the asexual blood stage most susceptible to the tested compounds, we followed a previously established protocol.[15] Schizont-stage parasites were first enriched using magnetic separation with MACS LS columns (Miltenyi Biotec) from cultures synchronized multiple times with 5% sorbitol. Following enrichment, the parasites were allowed to re-invade fresh red blood cells at 2% hematocrit. A second sorbitol synchronization was then performed to generate a highly synchronized ring-stage

culture (designated as time = 0 h). Synchronized parasites were distributed into five 96-well plates and exposed to the test compounds at different developmental stages: ring stage (0–16 h), trophozoite stage (16–32 h), or schizont stage (32–48 h). Parasite growth was evaluated at 60 h, a time point at which parasites had completed reinvasion and developed into trophozoites. Parasite viability after both 16 h stage-specific exposures and 72 h continuous exposure was determined using the SYBR Green fluorescence assay. IC_{50} values were calculated from the growth inhibition data by nonlinear regression using GraphPad Prism 8.0.1. Each experiment was performed independently twice, with two technical replicates per experiment.

Caco-2 cell permeability testing

For the Caco-2 cell permeability assay the Caco-2 cell monolayer was plated in a 96 well format (0.4 µm pore-sizes, 0.143 cm^2 surface area). The Caco-2 cell line was procured from Evotec and cells were cultured in 5% CO_2 in a humidified cell culture chamber at 37 ºC. The monolayers in the apical chamber were pre-incubated with pre-warmed HBSS (Hank's balanced salt solution) containing 10mM HEPES buffer (pH 7.4) and cells in the basolateral chamber received pre-warmed HBSS containing 10mM HEPES buffer (pH 7.4) and 1% BSA. Digoxin, Atenolol and Propranolol at a final concentration of 10µM were used as assay controls. A final DMSO concentration of 0.1% was used for the assay. Compounds were assessed at a final concentration of 2 µM and 10 µM in the assay.

On the day of the assay, Caco-2 cell monolayers were washed with transport buffer (HBSS, pH 7.4) and preincubated for 30 min (37 °C, 5% CO_2, 95% RH). Transport experiments were initiated by addition of HBSS buffer (pH 7.4) solution spiked with test compounds and permeability markers (10 µM) to the donor compartment in duplicate (n=2) wells and drug free HBSS buffer with 1% BSA (pH 7.4) to the receiver compartment. The volumes of apical and basolateral compartments are 0.075 and 0.25 mL, respectively. Plates are incubated in the incubator for 120 min at 37 °C. Samples were collected both from acceptor and receiver chambers at 120 min post assay initiation. Transport

experiments were conducted in both apical to basolateral (A-B) and in the basolateral to apical (B-A) directions. Concentration of test compounds in the samples are analyzed by liquid chromatography tandem mass spectrometry (LC-MS/MS) method using discovery grade bioanalytical method.

The integrity of Caco-2 cell monolayers were examined post experiment. Caco-2 cell monolayers were washed with pre-warmed HBSS buffer. Apical insert was dosed with 75 µL of 50 µM Lucifer Yellow (LY) and basolateral wells with 250 µL of HBSS buffer. Plates were incubated in the incubator for 60 min at 37 °C. After 60 min, samples (200 µL) were transferred from each of the basolateral receiver plate into 96 well black plates. The samples were read on spectrophotometer at an excitation wavelength of 428 nm and emission wavelength at 540 nm.

Apparent Permeability (P_{app}) was calculated using the following formula: Papp is expressed in units of 10^{-6} cm/sec.

$$\text{Papp} = \frac{dq}{dt} \text{X} \frac{1}{Co} \text{X} \frac{1}{A}$$

Where dq/dt = rate of transport (rate of transport of compound in the receiver compartment), Co = initial concentration in the donor compartment, A = surface area of the effective filter membrane.

Efflux Ratio was calculated using the following equation:

$$\text{Efflux ratio} = \frac{\text{Papp values in B} - \text{A direction}}{\text{Papp values in A} - \text{B direction}}$$

% Recovery was calculated using following equation:

$$\%\,\text{Recovery} = \frac{\text{Total compound in donor and reciver chamber at the end of transport experiment}}{\text{The initial amount of compound loaded in the donor chamber}} \times 100$$

Acceptance Criteria:

Permeability Markers	Papp x 10-6 cm/sec (A-B)	Papp x 10-6 cm/sec (B-A)
Propranolol	8.2-29.3	6.6-27.02
Atenolol	0.04-0.96	0.06-1.24
Digoxin	0.25-1.08	8.7-30.42

Kinetic solubility testing:

4 µL of master stock (10 mM) was added to 396 µL of 100 mM potassium phosphate buffer (pH 7.4) and incubated for 2 h at room temperature on a thermomixer at a shaking speed of 1100 rpm. Final DMSO concentration in the incubations was 1% v/v. After incubation, samples were filtered at 4000 rpm for 5 min in filter plate and supernatant was separated. Collected supernatants were subjected to HPLC analysis. Test samples were analyzed against 8-point calibration curve with concentrations ranging from 1 µM to 150 µM. Carvedilol and Diethylstilbesterol were used as assay controls with acceptance criteria being Carvedilol solubility > 55 µM and Diethylstilbesterol solubility being ≤ 10 µM.

Mouse and human liver microsome stability:

Mouse and human liver microsomes (MLMs and HLMs respectively) were purchased from Xenotech, (MLMs- catalog no: M5000, lot no: 2210070), (HLMs- catalog no: H2620, lot no: 1910096). Liver microsome (LMs) at a final concentration of 0.56 mg/ml were prepared mixing 5ul of LM stock solution 20mg/ml to 173 µL of 100mM potassium phosphate buffer , pH 7.4 to prepare the microsomal mix. 2 µL of test compound working stock (100 µM) solution was added to 178 µL of buffer microsomal mix and preincubated for 15 min at 37 ± 1 °C (600 RPM) into the wells of a 96 well plate. After preincubation, 45 µL (0 min sample) of preincubation mixture was precipitated with 400 µL of ice-cold quenching solution containing internal standard (50 ng/mL of Tolbutamide and 50 ng/mL of Telmisartan) and 5 µL of 10 mM NADPH was added. 45 µL of preincubation mixture was mixed with 5 µL of buffer and incubated for 30 min (-NADPH sample). The reaction was initiated by addition of 10 µL of cofactor (10 mM) to the preincubation mixture and incubated on a thermomixer maintaining 37 ± 1 °C (600 RPM). 50 µL of incubation mixture was taken at 30 min time point and stopped by addition of 400 µL of ice-cold quenching solution containing internal standard (50 ng/mL of Tolbutamide and 50 ng/mL of Telmisartan). The samples were vortexed for 10 min at 1000 RPM and

centrifuged at 4000 RPM for 30 min at ~6 °C. The supernatants were collected into 96-well plate as per the cassette combination and samples were analyzed by LC-MS/MS. For -NADPH samples, the reaction was stopped at 30 min with addition of 400 µL of ice-cold quenching solution containing internal standard (50 ng/mL of Tolbutamide and 50 ng/mL of Telmisartan) and processed in the above manner. Verapamil and Diclofenac were used as positive controls for the assay. The percentage of parent compound remaining (%PCR) at a specific time point was calculated using LCMS as:

$$\%\ PCR = \frac{\text{Area ratio at Xmin}}{\text{Area ratio at 0 min}} \times 100$$

where X is the final time point.

Human and mouse plasma stability assay:

Intermediate stock solution (100 µM) of test compound (4 µL) was spiked to 396 µL of plasma and incubated for 5 h at 37°C. At each time point (0, 15, 30, 60, 120 and 300 min), 50 µL of incubation mixture was precipitated with 300 µL of ice-cold acetonitrile containing internal standard (250 ng/mL). Samples were vortexed for 10 min at 1000 rpm and centrifuged at 4000 rpm for 10 min. After centrifugation, 100 µL of supernatant was diluted with 100 µL of water and submitted for LC-MS/MS analysis. Propantheline was used as a positive control for the assay. The percentage of parent compound remaining (%PCR) at a specific time point and half-life was calculated using LCMS.

Human/mouse plasma protein binding assay:

Human/mouse plasma protein binding was determined using the Rapid Equilibrium Dialysis method (RED) (200 µL Plasma/350 µL Buffer). Human plasma was thawed to 37°C and centrifuged at 4000 rpm for 10 min. Clear supernatant was separated and used for the assay. 1 µL of working stock (2 mM) was spiked to 999 µL of preincubated plasma, to give a final concentration of 2 µM (DMSO ~0.1%). 200 µL of compound spiked plasma was added to the RED chamber and 350 µL of PBS buffer was added to the buffer chamber. Teflcon block consisting of RED inserts was incubated for 5 h on a

thermomixer at a shaking speed of 450 rpm. T0 control plasma samples were matrix matched with equal volume (1:1, v/v) of PBS buffer and precipitated with 300 µL of acetonitrile containing internal standard (Telmisartan). At 5 h, 50 µL of sample was aliquoted from both red and buffer chamber. These samples were matrix matched and precipitated with 300 µL of acetonitrile containing internal standard. Samples were vortexed at 1050 rpm for 3 minutes and centrifuged at 4000 rpm for 14 min. After centrifugation, 100 µL of supernatant was diluted with 100 µL of Milli Q water and submitted for LC-MS/MS analysis.

Table S1. *In vitro* antiplasmodial activity against chloroquine-sensitive (3D7) strain of *P. falciparum*, cytotoxicity against HepG2 cell line and selectivity index of quinacrine derivatives. Mean values are represented with their respective standard deviations.

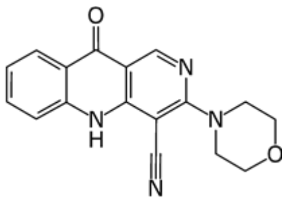

5b

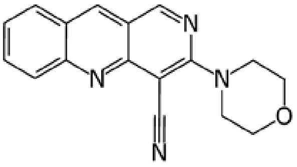

6

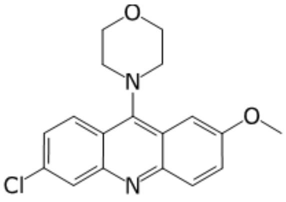

7

**Compound**	**IC_{50} *Pf*^{3D7} (nM)**	**CC_{50} HepG2 (µM)**	**SI**
**5b**	> 10000	n.d.	n.d.
**6**	> 10000	n.d.	n.d.
**7**	3300 ± 600	n.d.	n.d.

n.d. = not determined

Table S2. Candidate genes identified by whole-genome sequencing in **5a**-resistant lineage.

Gene ID	Gene name	SNPs	Clones
PfDd2_030025400	T-Complex protein 1 (TCP-1) subunit epsilon	M521I	5a_B_C3;
PfDd2_140031600	Putative Lipase	I1264N	5a_B_A8; 5a_B_H4
Dd2_050017100	Glutathione synthetase (GS)	M42I	5a_A_C7; 5a_A_H4; 5a_B_C3

Table S3. Summary of mutations identified by Sanger sequencing across isolated clones.

Clones		TCP-1	Putative lipase	GS
Dd2-Polδ (parental line)		0	0	0
**Flask A**	A10	0	0	1
	B8	NI	NI	NI
	C7	0	0	1
	E12	0	0	1
	F12	NI	NI	NI
	H4	0	0	1
**Flask B**	A8	1	1	0
	A10	1	1	0
	B10	1	1	0
	C3	1	1	0
	C4	1	1	0
	H4	1	1	0
**Flask C**	B6	0	0	1
	C4	0	0	0
	C8	0	0	1
	F5	0	0	0
	F6	0	0	0
	G6	0	0	1

NI = not identified.

Table S4. Predicted *in vitro* ADME properties for **2d** (12126038). Highlighted has also been determined experimentally. Due to the nature of the prediction scores, typically a threshold of 0.5 is used to determine if a compound is predicted to be active, but higher scores suggest a higher likelihood of activity. Applicability scores are based on the reliability-density neighborhood (RDN) method which considers both the model overlap and the individual bias and precision of the overlapping fingerprints. There is not a defined threshold for an acceptable applicability score, but it is ideal to have a higher score for confidence in the prediction (1 max). The consensus scores are based on majority rule classification (agreement for ≥ 4 algorithms, when equal to 4 a compound is predicted as active). The threshold for activity is shown for each model.

ADME property	Applicability Domain	Consensus Prediction
Acute Oral Toxicity (EPA category I – II; ≤50 mg-kg)	0.67	0
Acute Oral Toxicity (GHS category of I,II, or III; ≤2,000mg/kg)	0.37	1
AMES Mutagenicity (binary)	0.37	0
BBB Penetration (human; binary)	0.43	0
Caco2 permeability (≥$1x10^{-5}$ cm/s.)	0.59	1
CYP inhibition (CYP1A2, CYP2B6, CYP3A4, CYP2D6, CYP2C19, CYP2C9)	0.67, 0.44, 0.58, 0.37, 0.6, 0.75	0, 0, 0, 0, 0, 0
Hepatocyte Clearance (Human; ≥6 ml/min/kg)	0.37	1
Hepatocyte Metabolic Clearance (Human; ≤9.5 μL/min/mg) AstraZeneca	0.37	0
hERG Toxicity (binary)	0.59	1
Human Bioavailability [ChEMBL with literature] (>50%, >70%)	0.50, 0.41	1, 0
Human Clearance (Cl, >4 mL min-1 kg-1)	0.51	1
Human Clearance ($t_{1/2}$ >2, >4, >15hr)	0.56, 0.45, 0.32	1, 1, 0
Human intestinal absorption (HIA) (>90%, >80%)	0.38, 0.51	0, 1
Human Microsomal Stability (30, 60 min; ≥70%)*	0.53, 0.41	1, 1
Human oral bioavailability (binary)	0.50	1
Human Plasma Stability ($t_{1/2}$ ≥50 min)*	0.23	1
Microsomal Stability (Mouse; ≥ 59.5 min)*	0.58	0
P-glycoprotein inhibition (≤ 1μM)	0.26	0
P-glycoprotein Substrate (Binary)	0.33	0
Plasma Protein Binding (Human) (>90%, >50%)*	0.43, 0.60	1, 1

*ADME properties experimentally validated (Data available in the main text).

Table S5. Predicted *in vitro* ADME properties for **2c** (12126039). Highlighted has also been determined experimentally. Due to the nature of the prediction scores, typically a threshold of 0.5 is used to determine if a compound is predicted to be active, but higher scores suggest a higher likelihood of activity. Applicability scores are based on the reliability-density neighborhood (RDN) method which considers both the model overlap and the individual bias and precision of the overlapping fingerprints. There is not a defined threshold for an acceptable applicability score, but it is ideal to have a higher score for confidence in the prediction (1 max). The consensus scores are based on majority rule classification (agreement for ≥ 4 algorithms, when equal to 4 a compound is predicted as active). The threshold for activity is shown for each model.

ADME property	Applicability Domain	Consensus Score
Acute Oral Toxicity (EPA category I – II; ≤50 mg-kg)	0.81	0
Acute Oral Toxicity (GHS category of I,II, or III; ≤2,000mg/kg)	0.44	1
AMES Mutagenicity (binary)	0.51	0
BBB Penetration (human; binary)	0.30	1
Caco2 permeability (≥$1x10^{-5}$ cm/s.)	0.36	1
CYP inhibition (CYP1A2, CYP2B6, CYP3A4, CYP2D6, CYP2C19, CYP2C9)	0.56, 0.75, 0.35, 0.52, 0.48, 0.25	0, 0, 0, 0, 0, 0
Hepatocyte Clearance (Human; ≥6 ml/min/kg)	0.38	0
Hepatocyte Metabolic Clearance (Human; ≤9.5 µL/min/mg) AstraZeneca	0.39	0
hERG Toxicity (binary)	0.36	1
Human Bioavailability [ChEMBL with literature] (>50%, >70%)	0.44, 0.47	1, 0
Human Clearance (Cl, >4 mL min-1 kg-1)	0.42	1
Human Clearance ($t_{1/2}$ >2, >4, >15hr)	0.66, 0.56, 0.66	1, 1, 0
Human intestinal absorption (HIA) (>90%, >80%)	0.47, 0.58	0, 1
Human Microsomal Stability (30, 60 min; ≥70%)*	0.41, 0.41	0, 0
Human oral bioavailability (binary)	0.39	1
Human Plasma Stability ($t_{1/2}$ ≥50 min)*	0.59	1
Microsomal Stability (Mouse; ≥ 59.5 min)*	0.53	0
P-glycoprotein inhibition (≤ 1µM)	0.58	0
P-glycoprotein Substrate (Binary)	0.42	0
Plasma Protein Binding (Human) (>90%, >50%)*	0.42, 0.58	1, 1

*ADME properties experimentally validated (Data available in the main text).

Table S6. Predicted *in vitro* ADME properties for **5a** (12126072). Highlighted has also been determined experimentally. Due to the nature of the prediction scores, typically a threshold of 0.5 is used to determine if a compound is predicted to be active, but higher scores suggest a higher likelihood of activity. Applicability scores are based on the reliability-density neighborhood (RDN) method which considers both the model overlap and the individual bias and precision of the overlapping fingerprints. There is not a defined threshold for an acceptable applicability score, but it is ideal to have a higher score for confidence in the prediction (1 max). The consensus scores are based on majority rule classification (agreement for ≥ 4 algorithms, when equal to 4 a compound is predicted as active). The threshold for activity is shown for each model.

ADME property	Applicability Domain	Consensus Score
Acute Oral Toxicity (EPA category I – II; ≤50 mg-kg)	0.83	0
Acute Oral Toxicity (GHS category of I,II, or III; ≤2,000mg/kg)	0.50	0
AMES Mutagenicity (binary)	0.38	0
BBB Penetration (human; binary)	0.33	1
Caco2 permeability (≥1x10^{-5} cm/s.)	0.31	1
CYP inhibition (CYP1A2, CYP2B6, CYP3A4, CYP2D6, CYP2C19, CYP2C9)	0.34, 0.64, 0.55, 0.72, 0.39, 0.65	0, 0, 0, 0, 0, 0
Hepatocyte Clearance (Human; ≥6 ml/min/kg)	0.34	1
Hepatocyte Metabolic Clearance (Human; ≤ 9.5 µL/min/mg) AstraZeneca	0.35	0
hERG Toxicity (binary)	0.51	0
Human Bioavailability [ChEMBL with literature] (>50%, >70%)	0.45, 0.33	1, 1
Human Clearance (Cl, >4 mL min-1 kg-1)	0.36	1
Human Clearance ($t_{1/2}$ >2, >4, >15hr)	0.23, 0.34, 0.35	1, 1, 0
Human intestinal absorption (HIA) (>90%, >80%)	0.33, 0.38	1, 1
Human Microsomal Stability (30, 60 min; ≥70%)*	0.43, 0.26	0, 1
Human oral bioavailability (binary)	0.52	1
Human Plasma Stability ($t_{1/2}$ ≥50 min)*	0.47	1
Microsomal Stability (Mouse; ≥ 59.5 min)*	0.33	0
P-glycoprotein inhibition (≤1 µM)	0.40	0
P-glycoprotein Substrate (Binary)	0.59	1
Plasma Protein Binding (Human) (>90%, >50%)*	0.48, 0.74	1, 1

*ADME properties experimentally validated (Data available in the main text).

Table S7. *In vitro* ADME properties for compounds **2d**, **2c**, and **5a**.

**Property/Compound**	**2d**	**2c**	**5a**
Solubility	108.88 µM at pH 7.4	1.24 µM at pH 7.4	0.06 µM at pH 7.4
Mouse liver microsomes	Parent compound remaining at 30 min = 77.5% (stable)	Parent compound remaining at 30 min = 82.8% (stable)	Parent compound remaining at 30 min = 2.4% (unstable)
Human liver microsomes	Parent compound remaining at 30 min = 92.6% (stable)	Parent compound remaining at 30 min = 98.6% (stable)	Parent compound remaining at 30 min = 0.5% (unstable)
Mouse plasma protein binding	% bound = 93.4% stability = 30.0	% bound = 96.5% stability = 22.3	% bound = 98.6% stability = 93.8
Human plasma protein binding	% bound = 88.7% stability = 23.7	% bound = 94.4% stability = 26.7	% bound = 99.6% stability = 88.2
In vitro stability in human plasma	Half-life = 138 min (unstable)	Half-life = 157 min (unstable)	Half-life = 1892 min (stable)
In vitro stability in CD-1 mouse plasma	Half-life = 77 min (unstable)	Half-life = 56 min (unstable)	Half-life = 928 min (stable)
Caco-2	Papp A-B = 0.37 x10^{-6} cm/s<br>Papp B-A = 9.65 x10^{-6} cm/s<br>Efflux ratio = 25.8	Papp A-B = 0.16 x10^{-6} cm/s<br>Papp B-A = 5.81 x10^{-6} cm/s<br>Efflux ratio = 36.4	Papp A-B = 5.92 x10^{-6} cm/s<br>Papp B-A = 0.76 x10^{-6} cm/s<br>Efflux ratio = 0.1

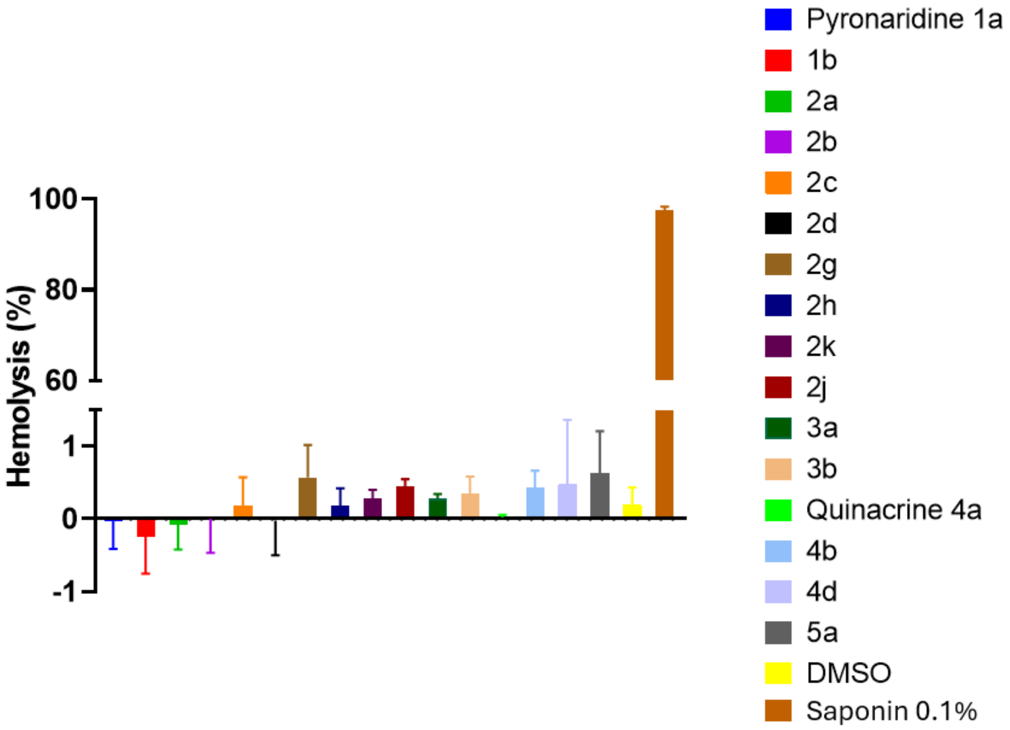

Figure S1. Hemolytic activity of test compounds at 10 µM on human red blood cells after 72 h. Hemolysis was quantified by measuring hemoglobin release at 540 nm and expressed as a percentage relative to untreated (negative control) and 0.1% saponin-treated (positive control) RBCs. DMSO at the highest concentration used served as the vehicle control.

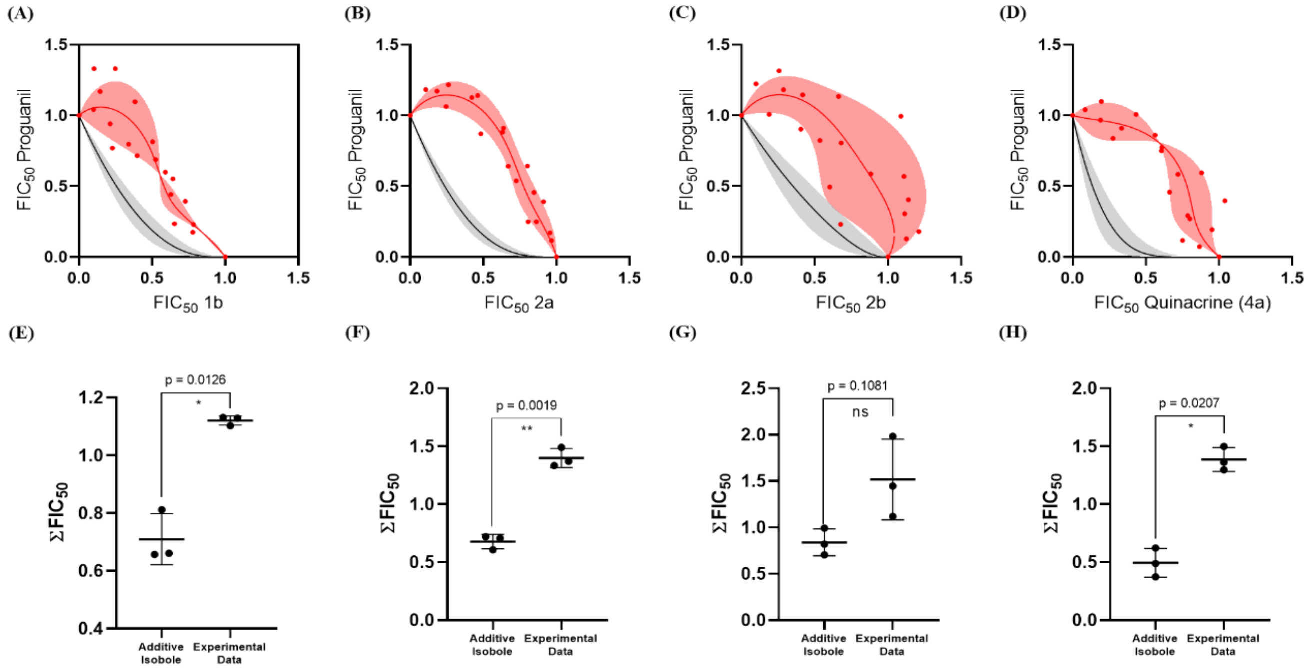

Figure S2. Isobolograms of association of compound **1b** (A), **2a** (B), **2b** (C) and quinacrine (D) with proguanil. Experimental data are represented by the red region and red dots, while the black line and gray region indicate the additivity curve. Statistical analysis for the combination of **1b** (**E**), **2a** (**F**), **2b** (**G**) and quinacrine (**H**) with proguanil. The data represent the ∑FIC50 values from three independent experiments. Statistical analysis was conducted by using Student's paired t-test (p-value < 0.027 indicates a statistical distinction between the experimental findings and the additivity isobole).

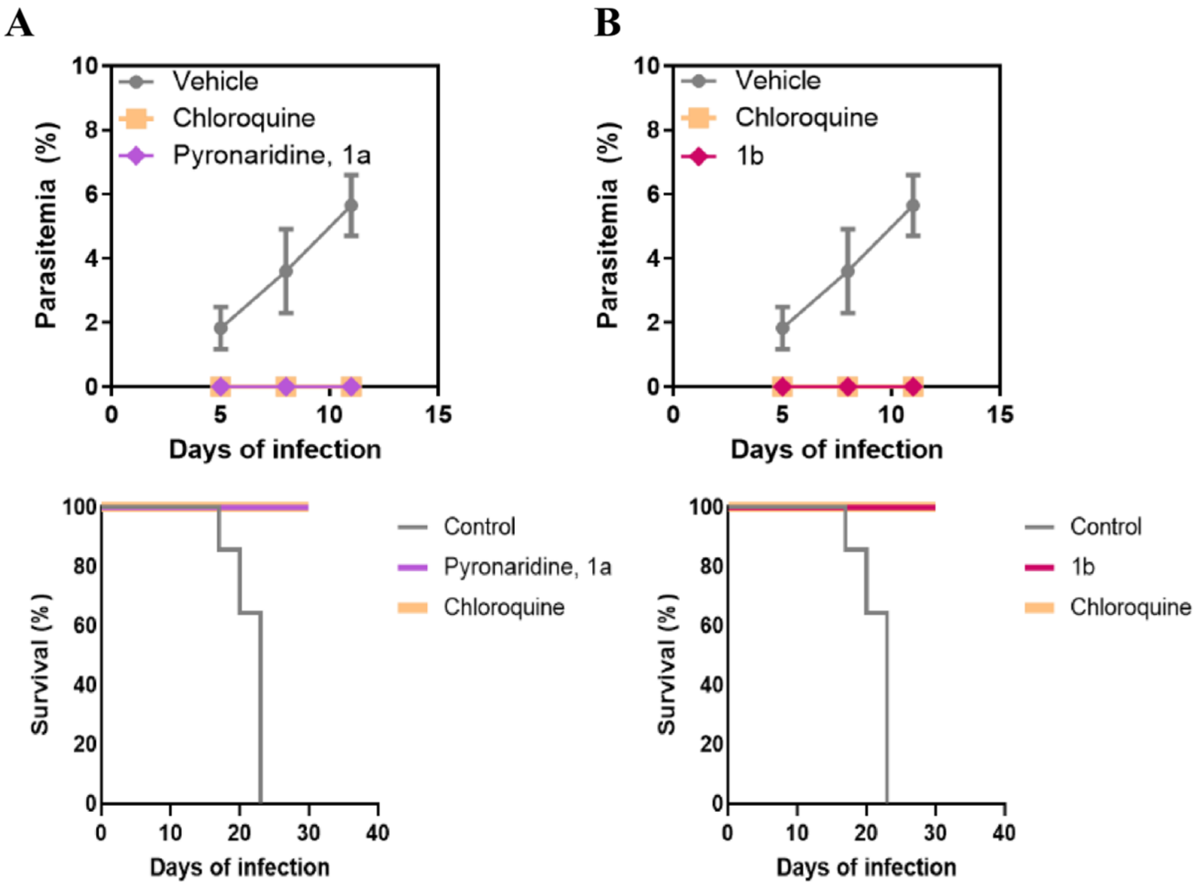

Figure S3. In vivo anti-malarial activity assessment and survival analysis in mice infected with *P. berghei* NK65. Upper panels: Parasitemia reduction following treatment with pyronaridine **1a** (**A**) and compound **1d** (**B**) at 50 mg/kg/day for three consecutive days post-infection is shown relative to the untreated control group. Chloroquine (20 mg/kg/day) was included as a reference antimalarial. Bottom panels: Survival analysis of mice treated (PO, 50 mg/kg/day) with compound pyronaridine **1a** (**A**) and **1d** (**B**) and standard antimalarial chloroquine evaluated during the experiment (30 days) (n = 3–4).

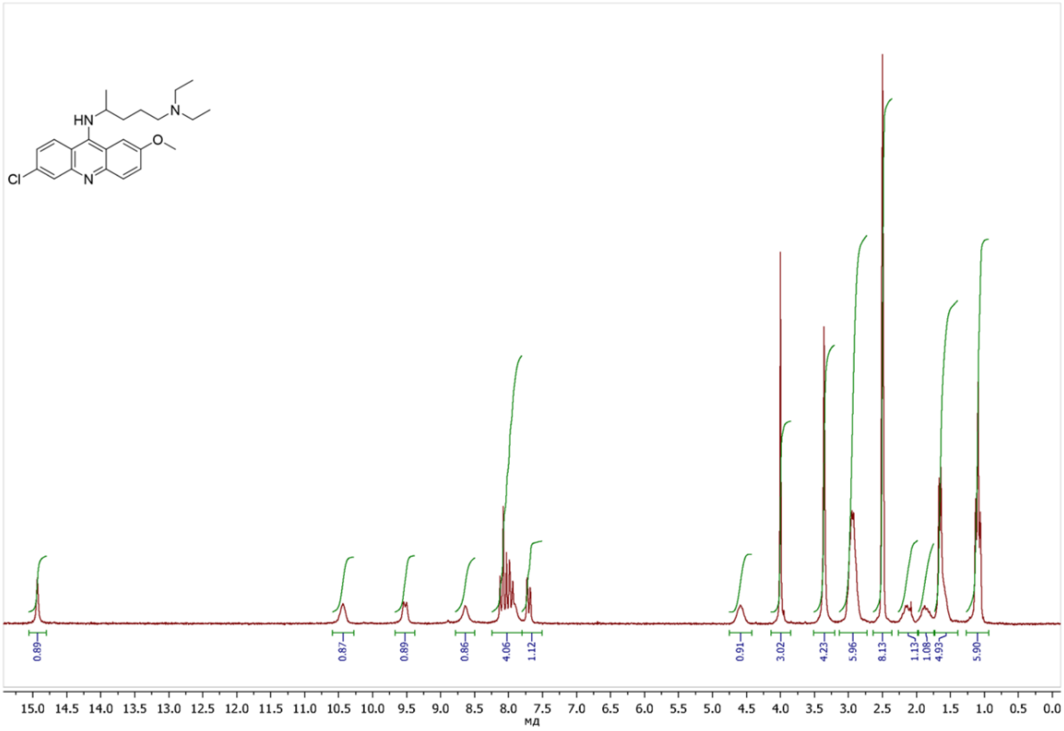

Figure S4. ^{1}H NMR spectrum (200 MHz, DMSO-d_6) of compound **4a.**

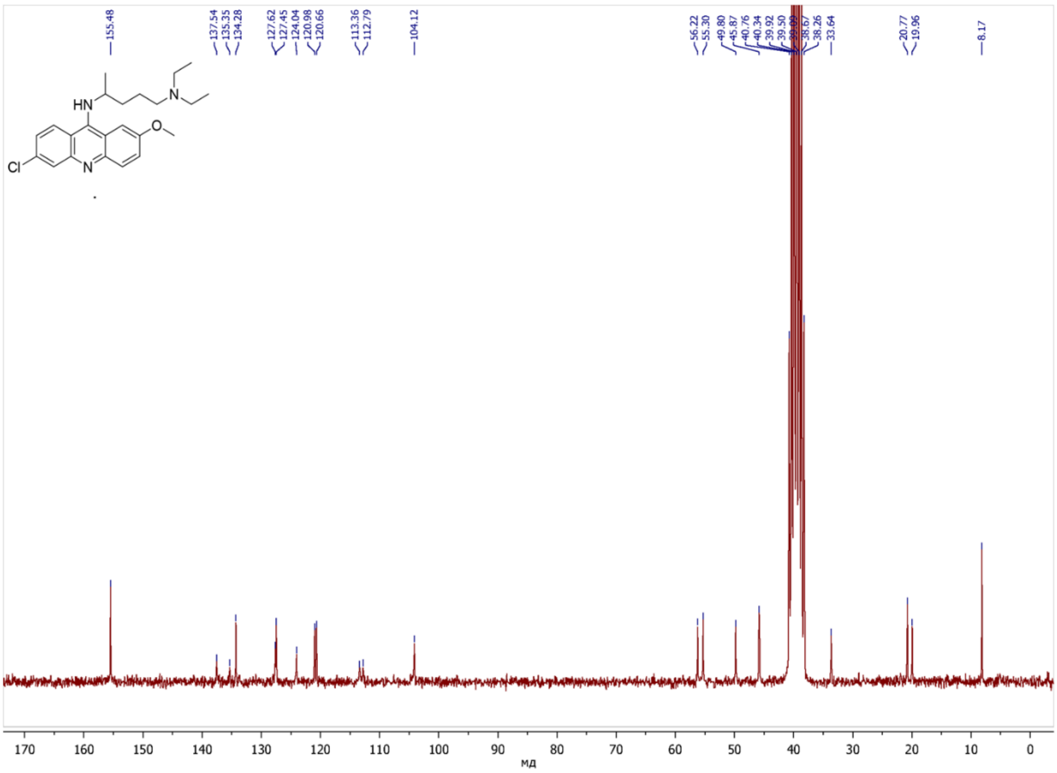

Figure S5. ^{13}C NMR spectrum (50 MHz, DMSO-d_6) of compound **4a**

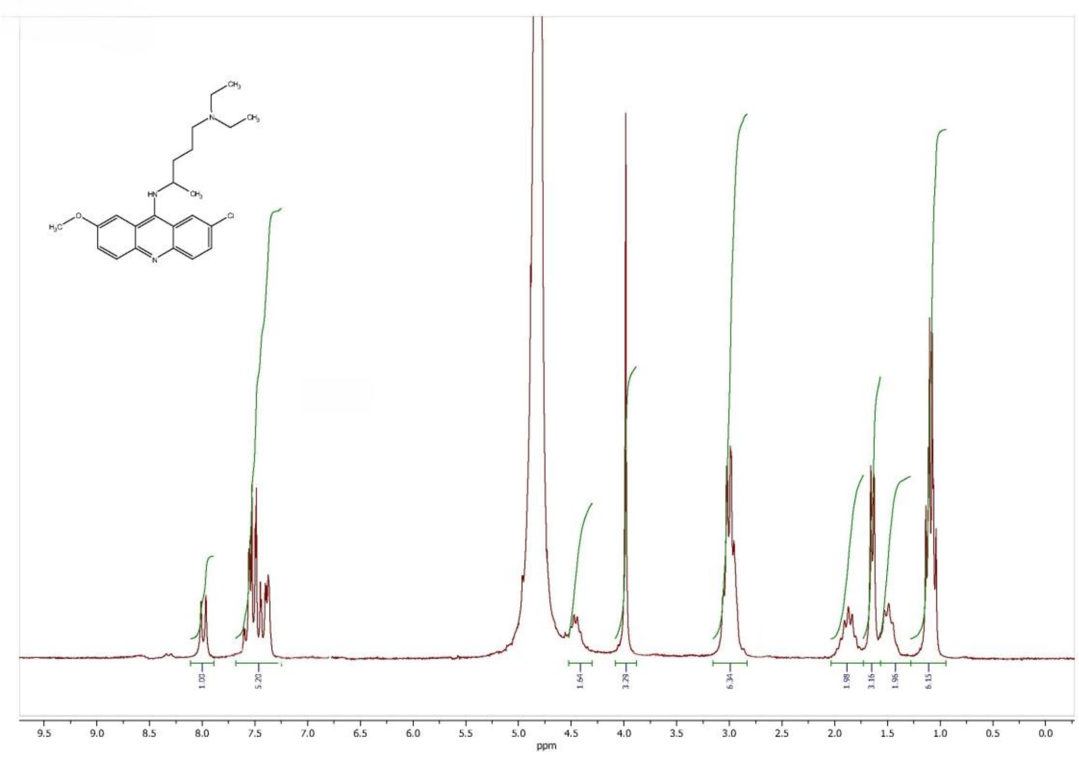

Figure S6. ^{1}H NMR spectrum (200 MHz, DMSO-d_6) of compound **4b**

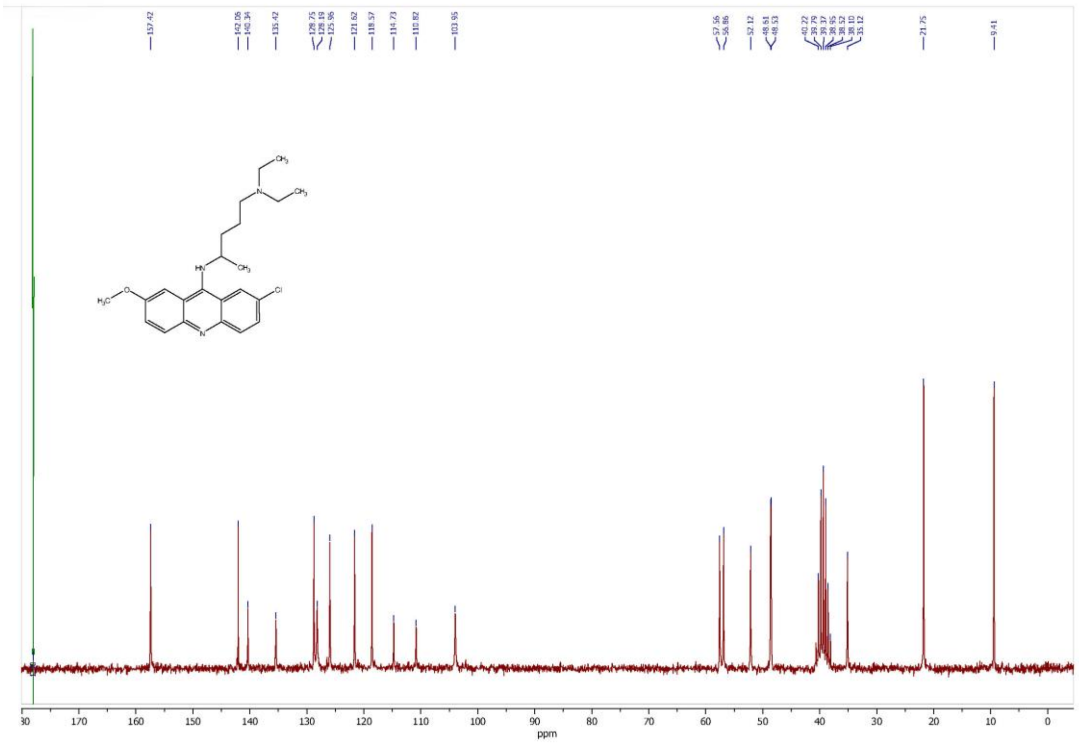

Figure S7. ^{13}C NMR spectrum (50 MHz, DMSO-d_6) of compound **4b**

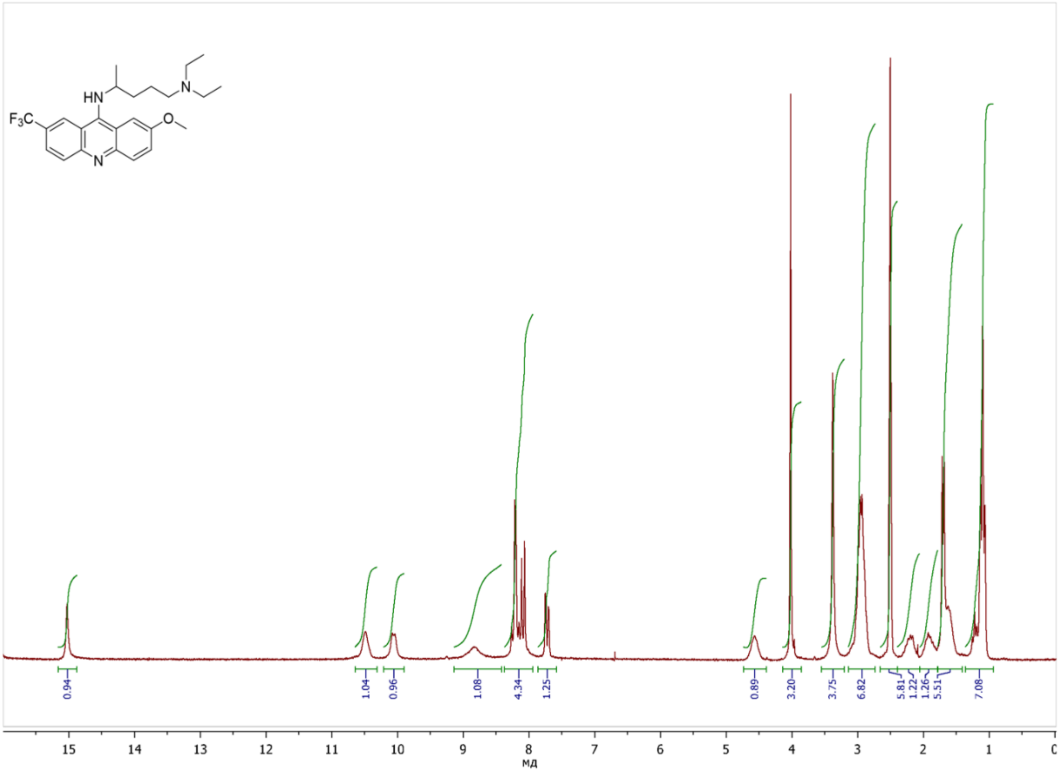

Figure S8. ^{1}H NMR spectrum (200 MHz, DMSO-d$_6$) of compound **4c**

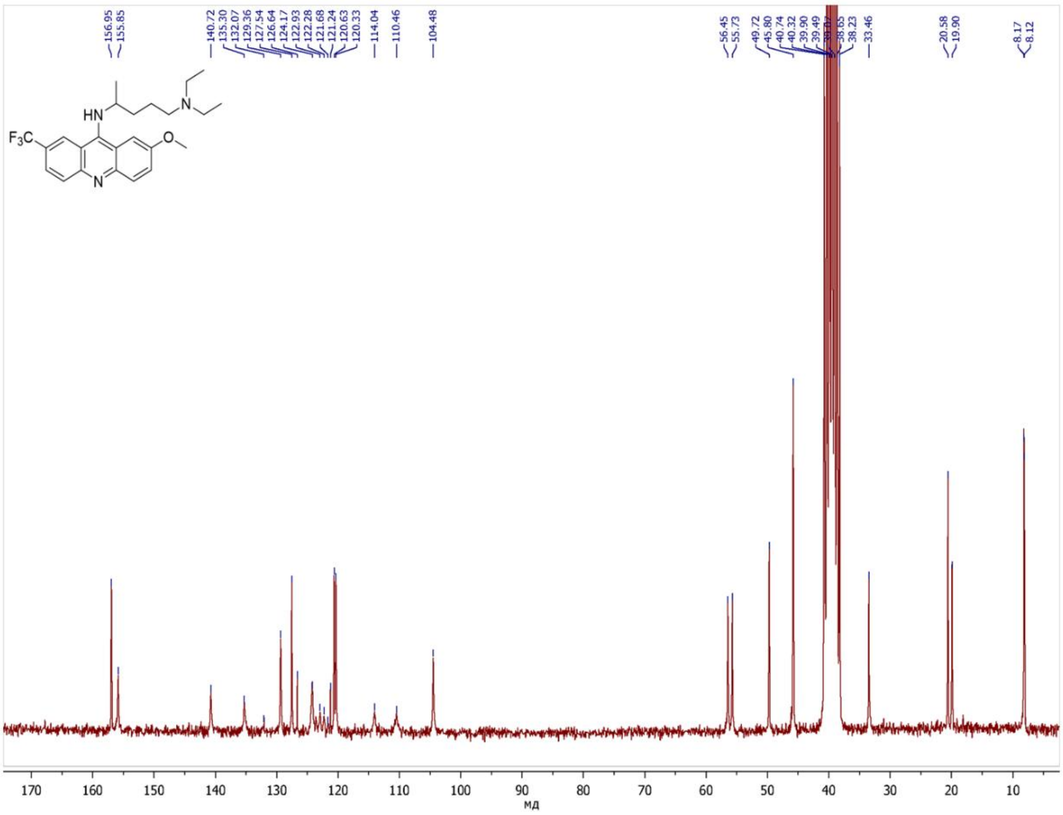

Figure S9. ^{13}C NMR spectrum (50 MHz, DMSO-d$_6$) of compound **4c**

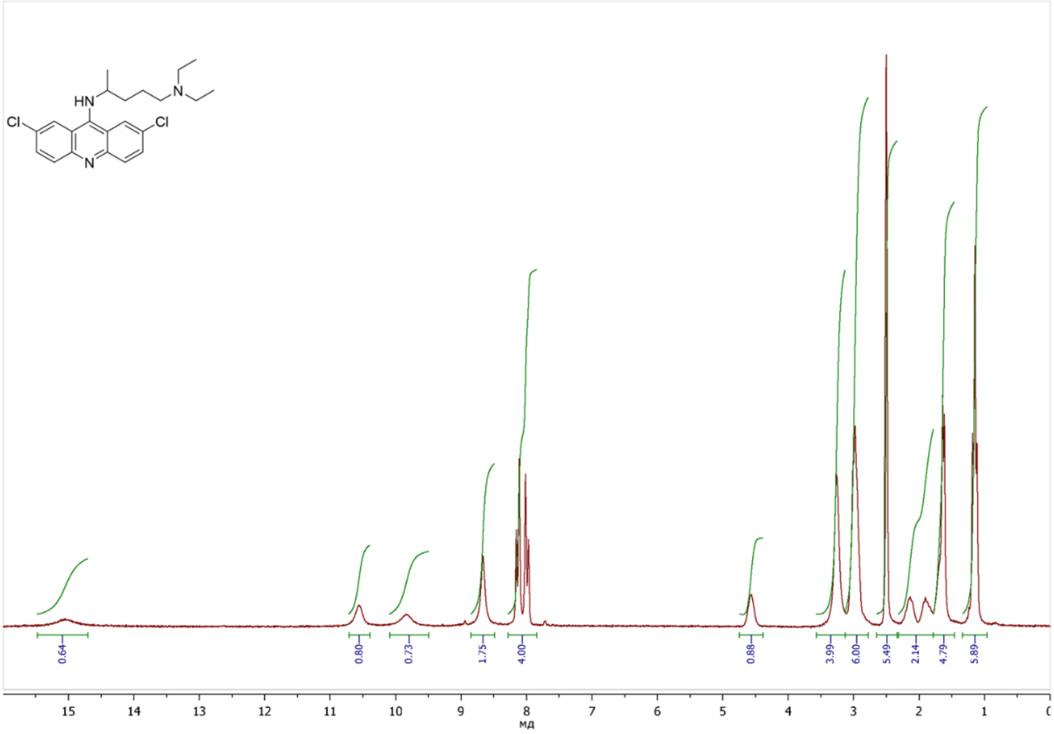

Figure S10. ^{1}H NMR spectrum (200 MHz, DMSO-d$_6$) of compound **4d**

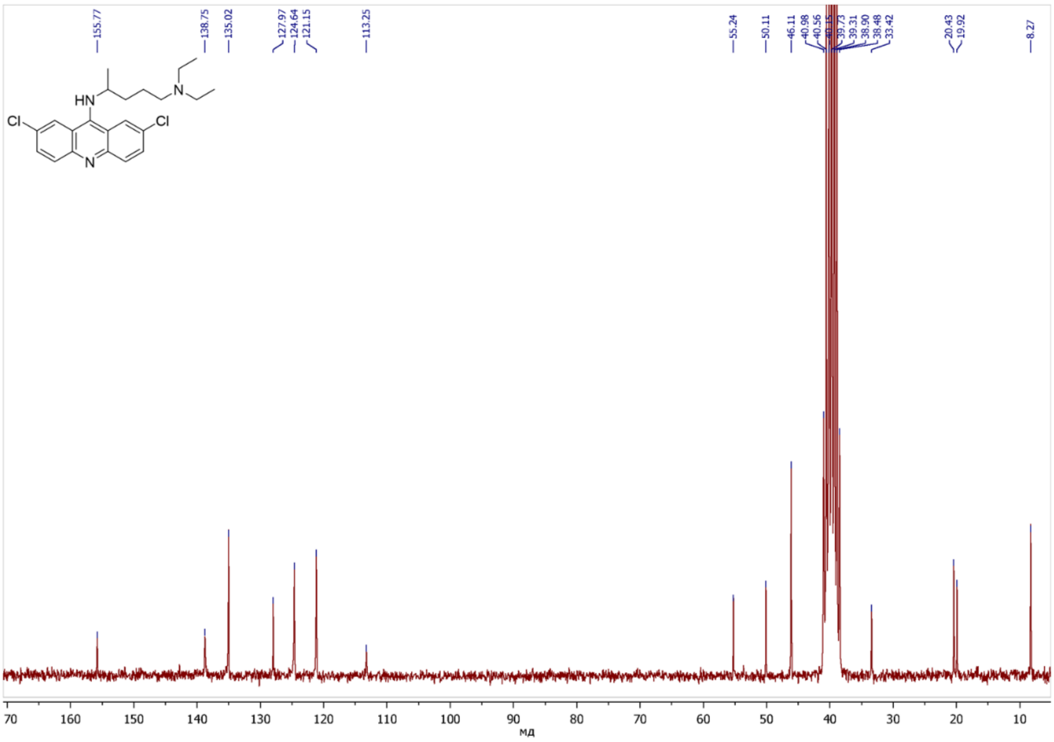

Figure S11. ^{13}C NMR spectrum (50 MHz, DMSO-d$_6$) of compound **4d**

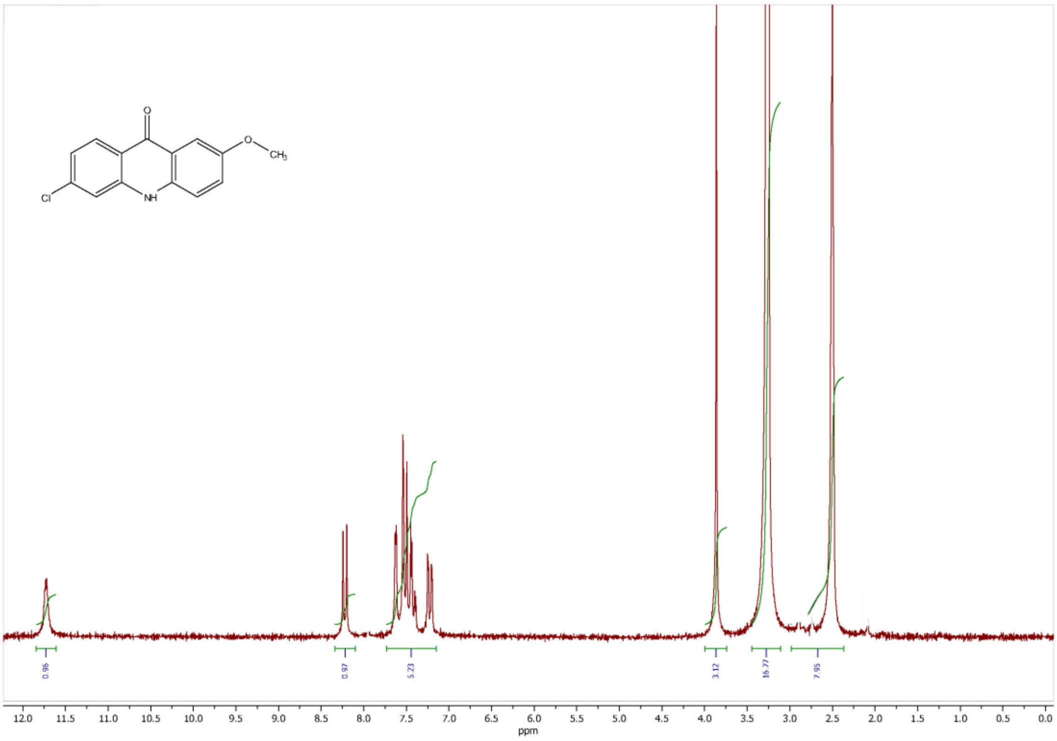

Figure S12. ^{1}H NMR spectrum (200 MHz, DMSO-d$_6$) of compound **5a**

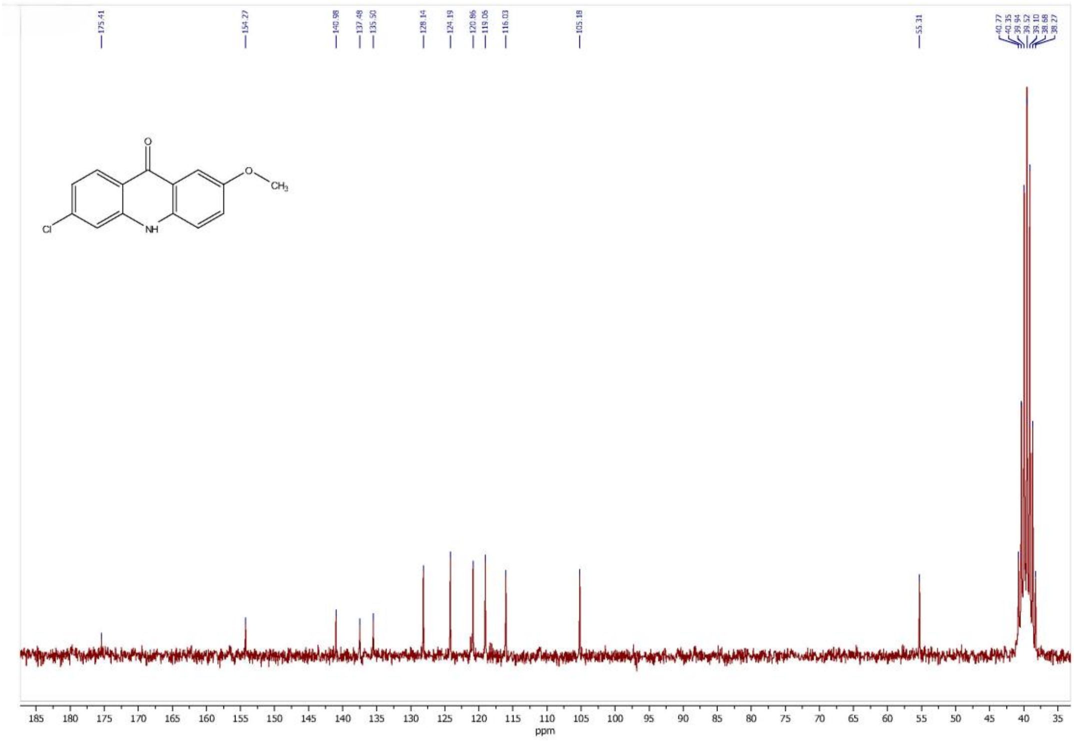

Figure S13. ^{13}C NMR spectrum (50 MHz, DMSO-d$_6$) of compound **5a**

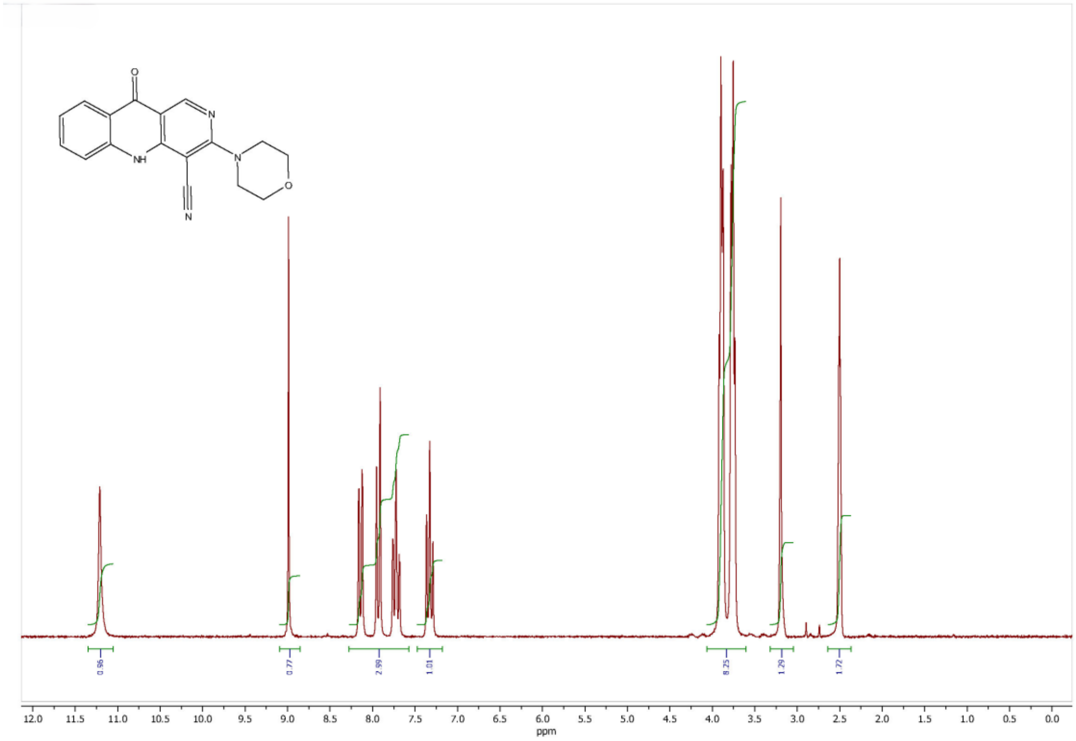

Figure S14. ^{1}H NMR spectrum (200 MHz, DMSO-d_6) of compound **5b**

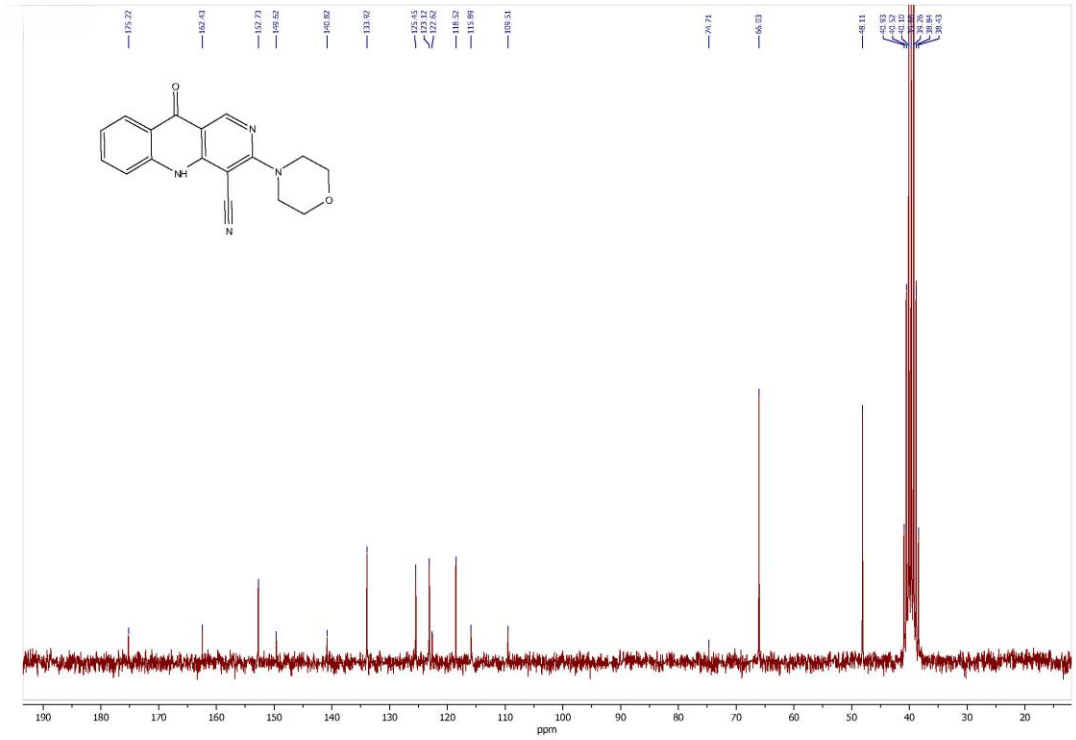

Figure S15. ^{13}C NMR spectrum (50 MHz, DMSO-d_6) of compound **5b**

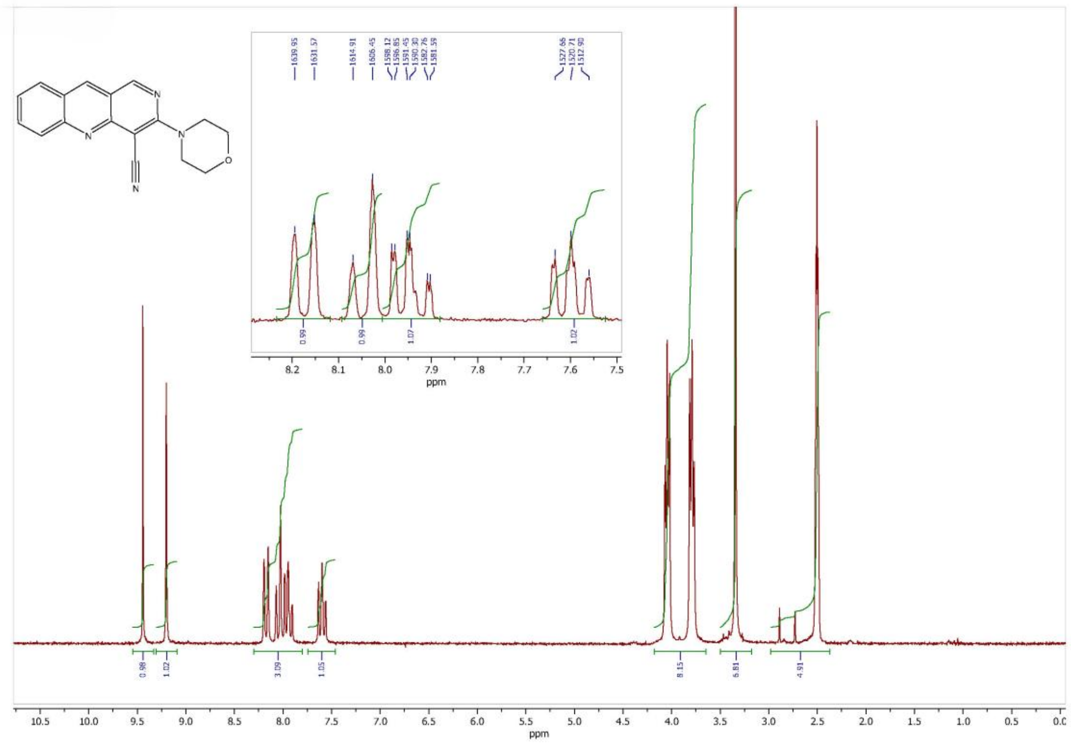

Figure S16. ^{1}H NMR spectrum (200 MHz, DMSO-d_6) of compound **6**

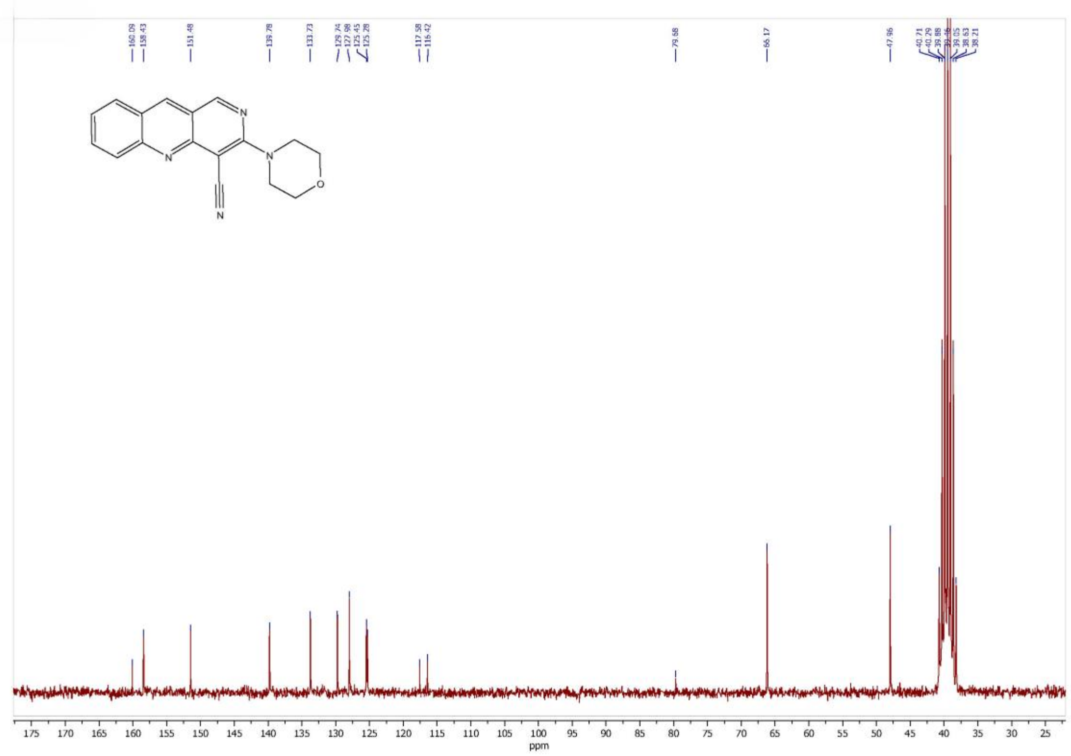

Figure S17. ^{13}C NMR spectrum (50 MHz, DMSO-d_6) of compound **6**

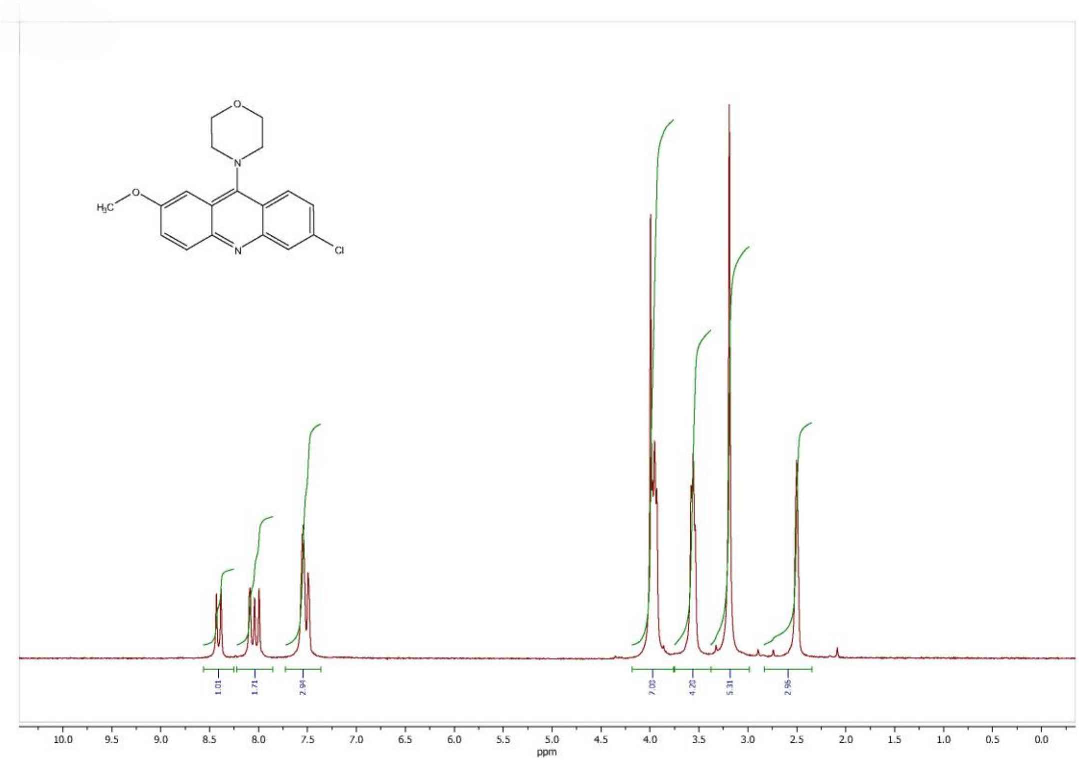

Figure S18. ^{1}H NMR spectrum (200 MHz, DMSO-d_6) of compound **7**

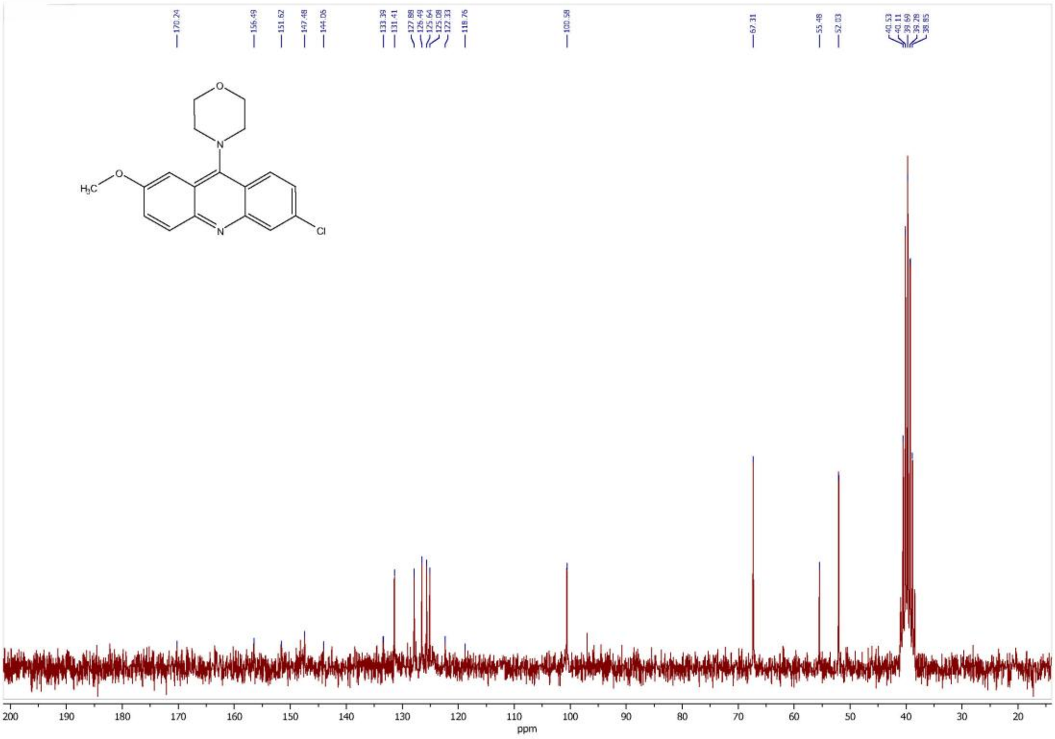

Figure S19. ^{13}C NMR spectrum (50 MHz, DMSO-d_6) of compound **7**

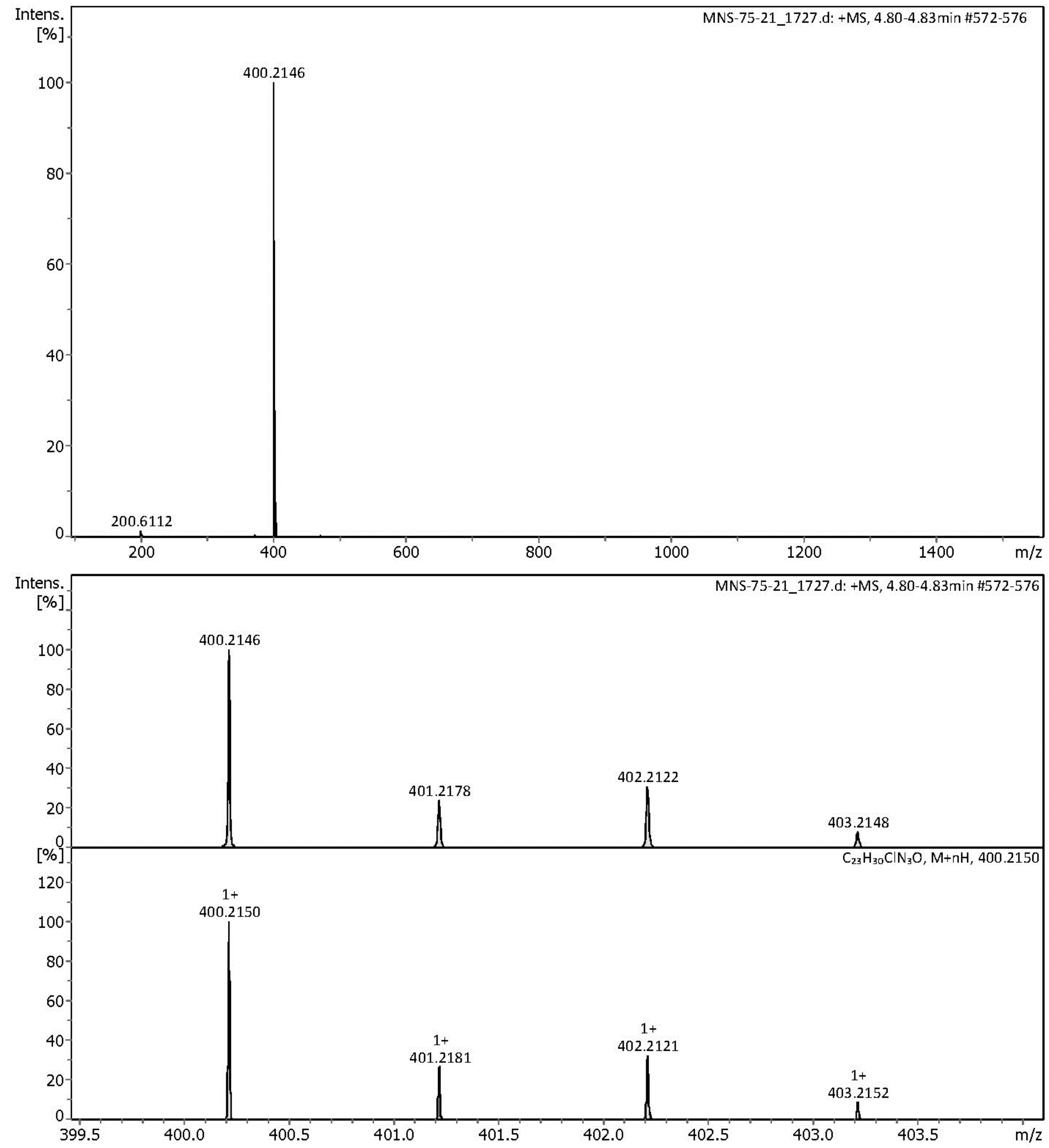

Figure S20. HR-MS data for compound **4a**

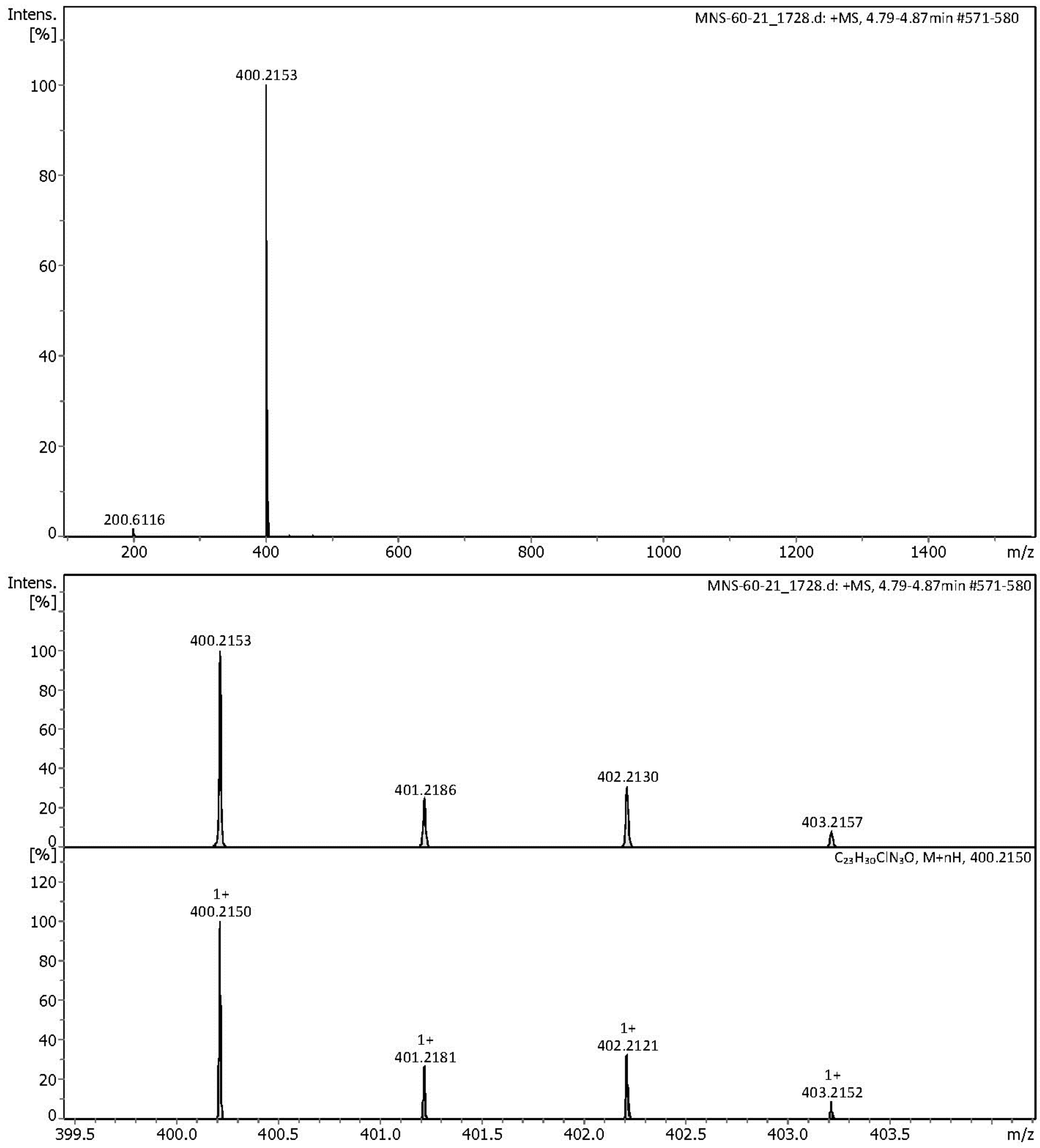

Figure S21. HR-MS data for compound **4b**

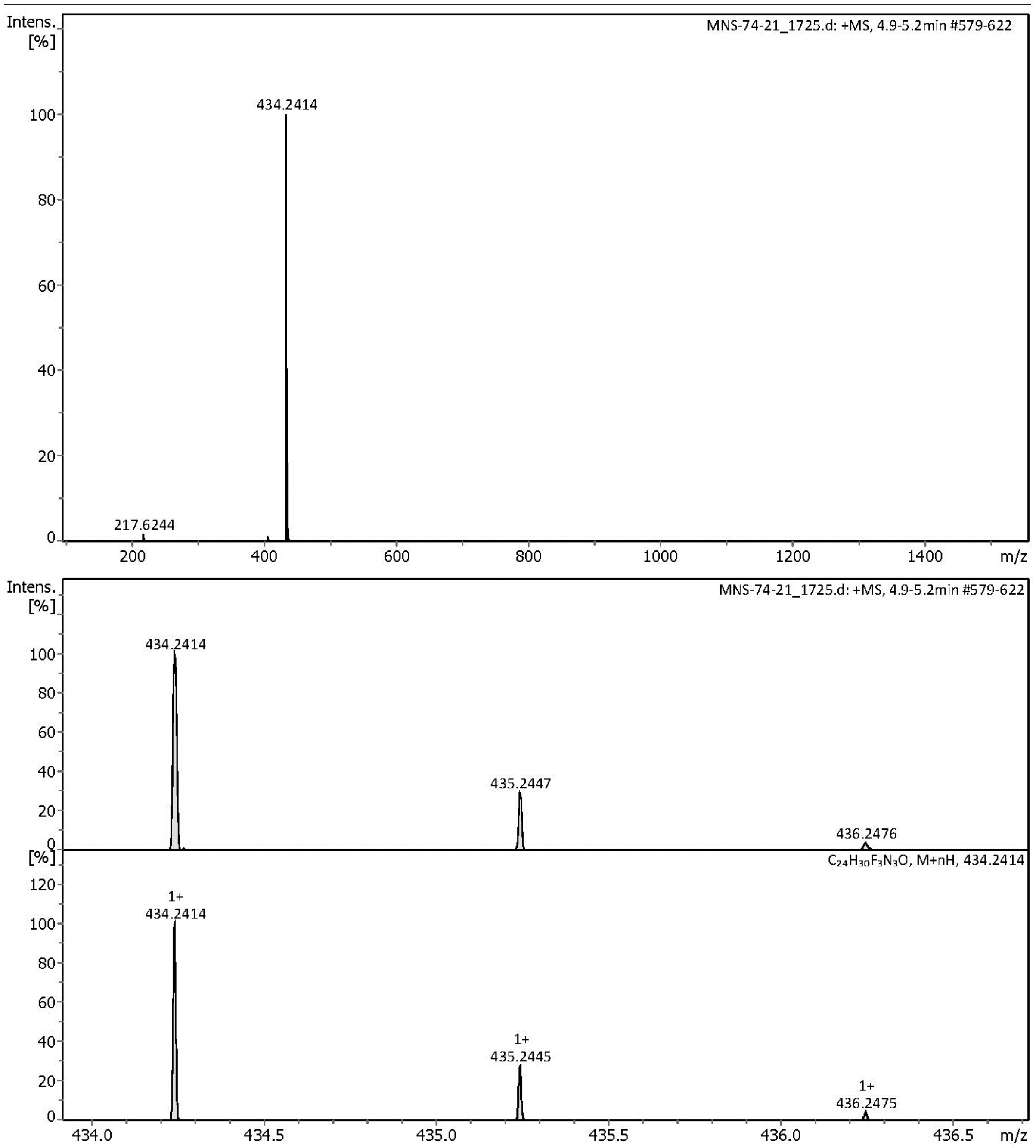

Figure S22. HR-MS data for compound **4c**

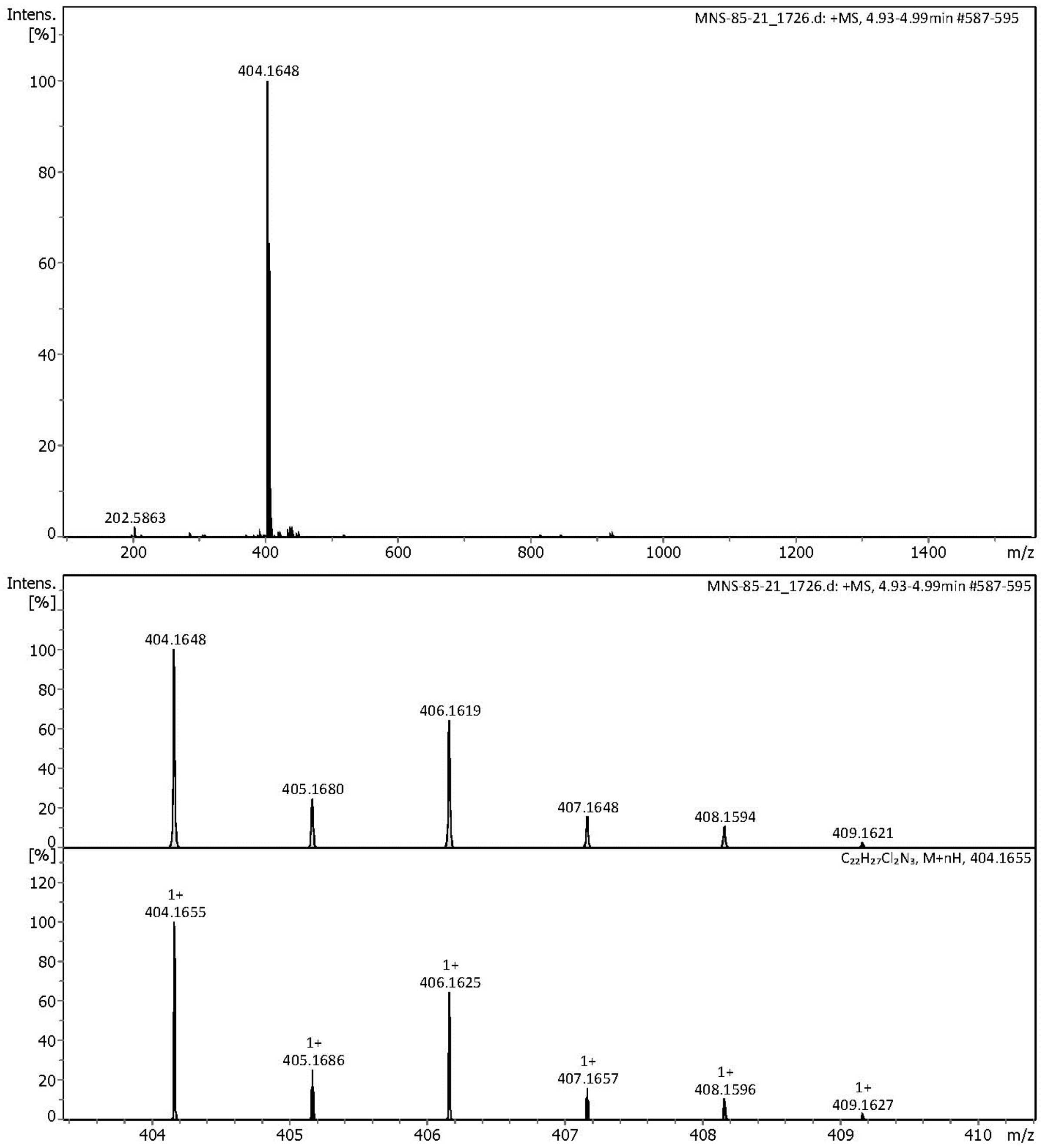

Figure S23. HR-MS data for compound **4d**

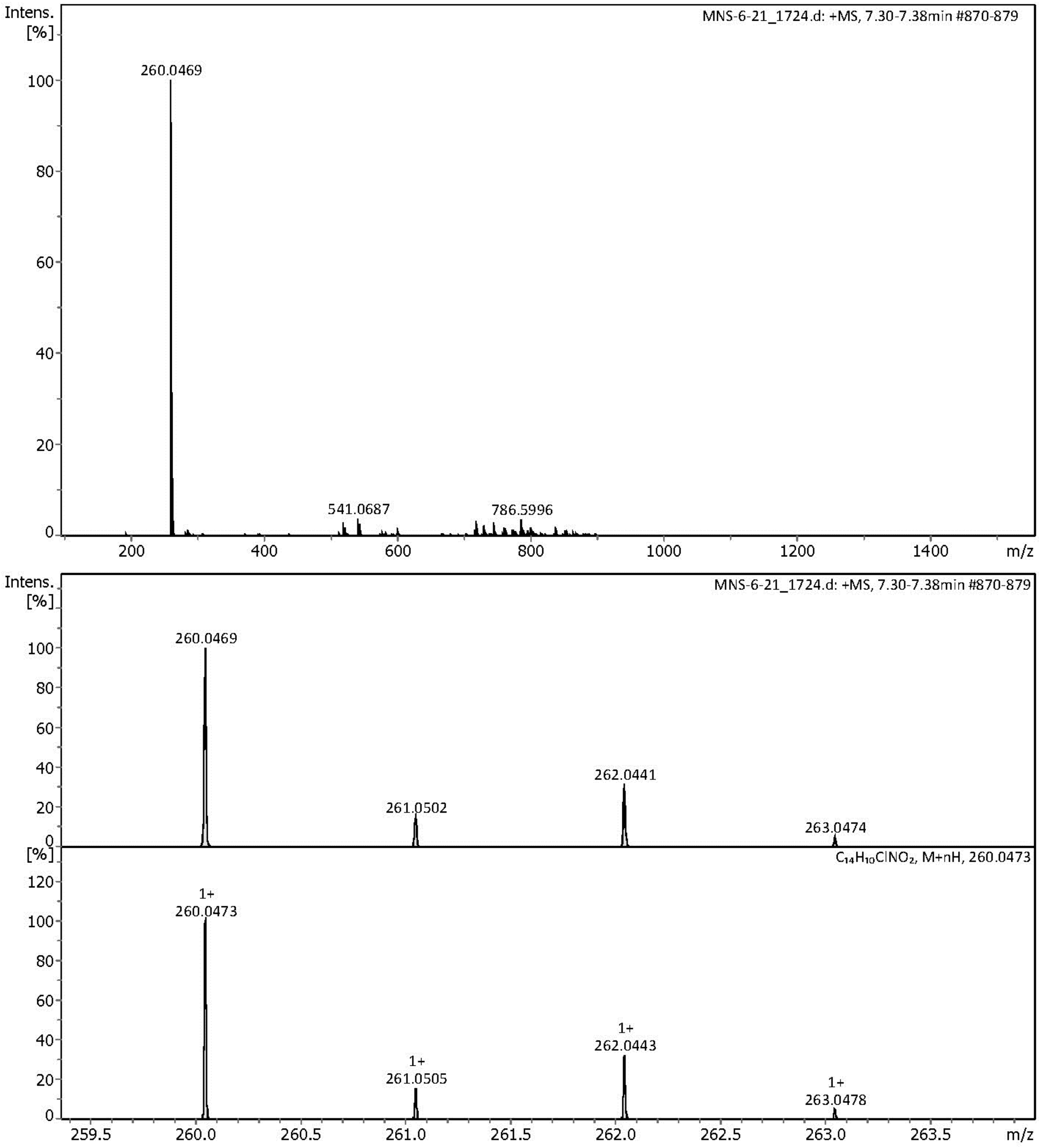

Figure S24. HR-MS data for compound **5a**

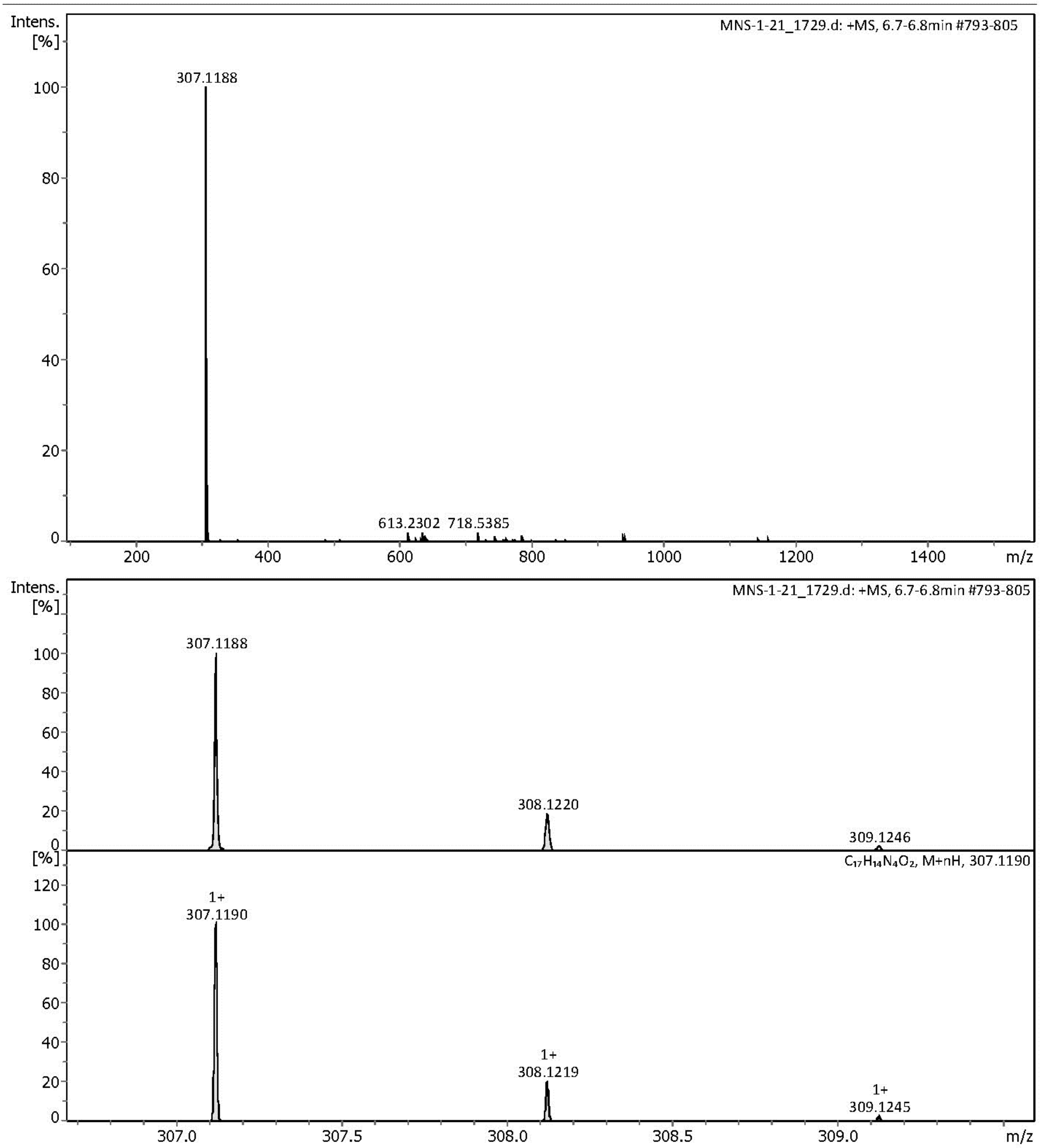

Figure S25. HR-MS data for compound **5b**

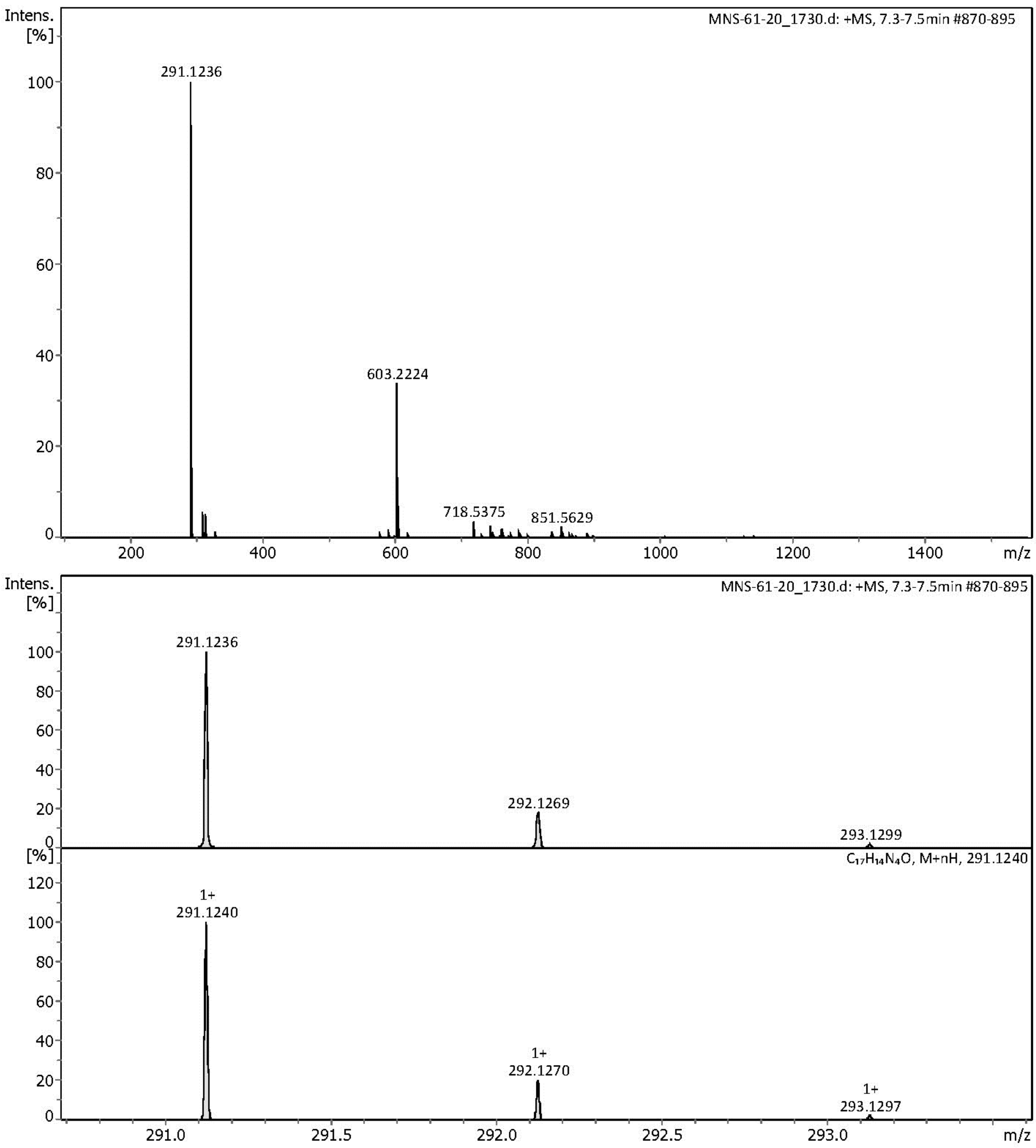

Figure S26. HR-MS data for compound **6**

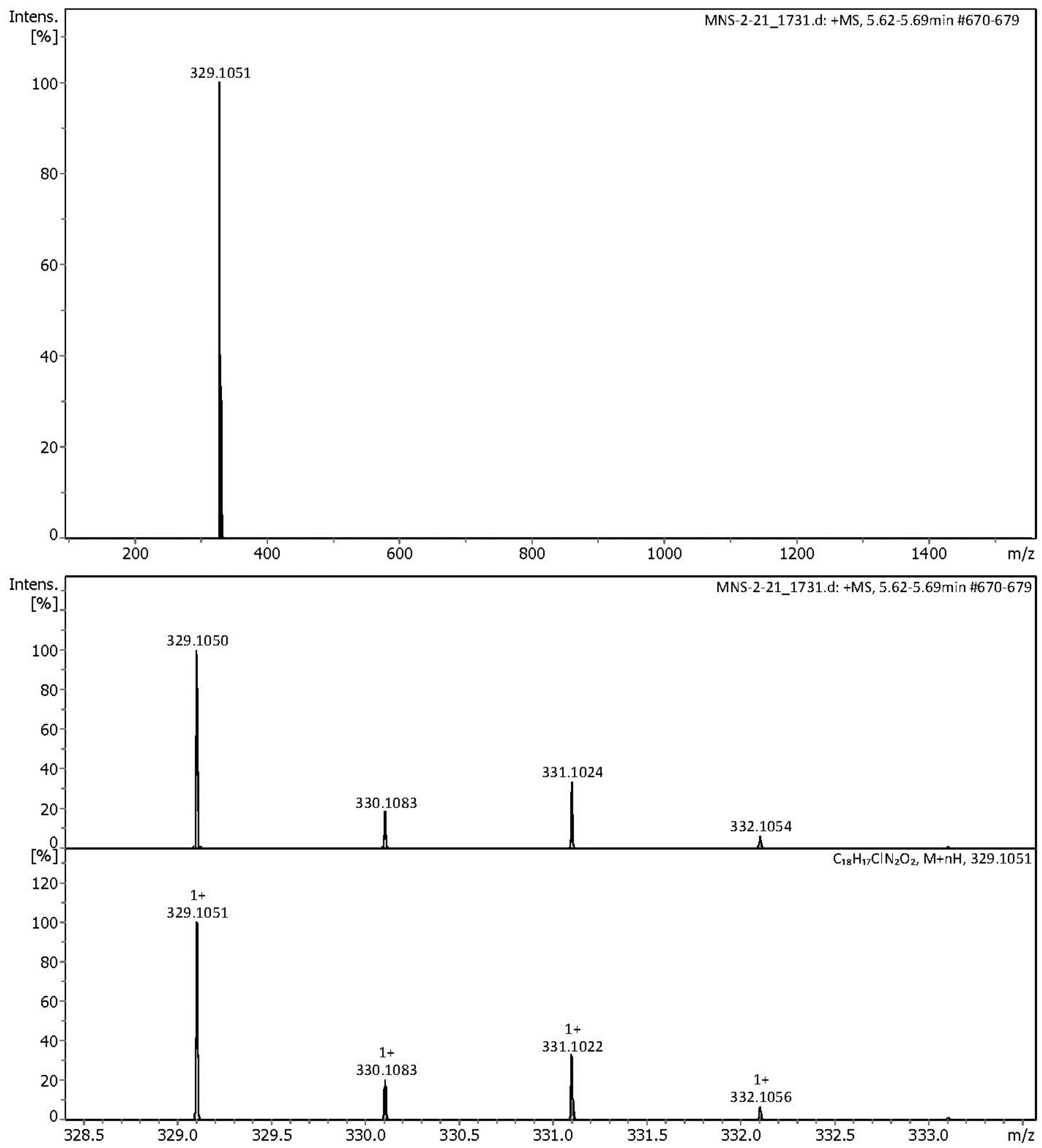

Figure S27. HR-MS data for compound **7**

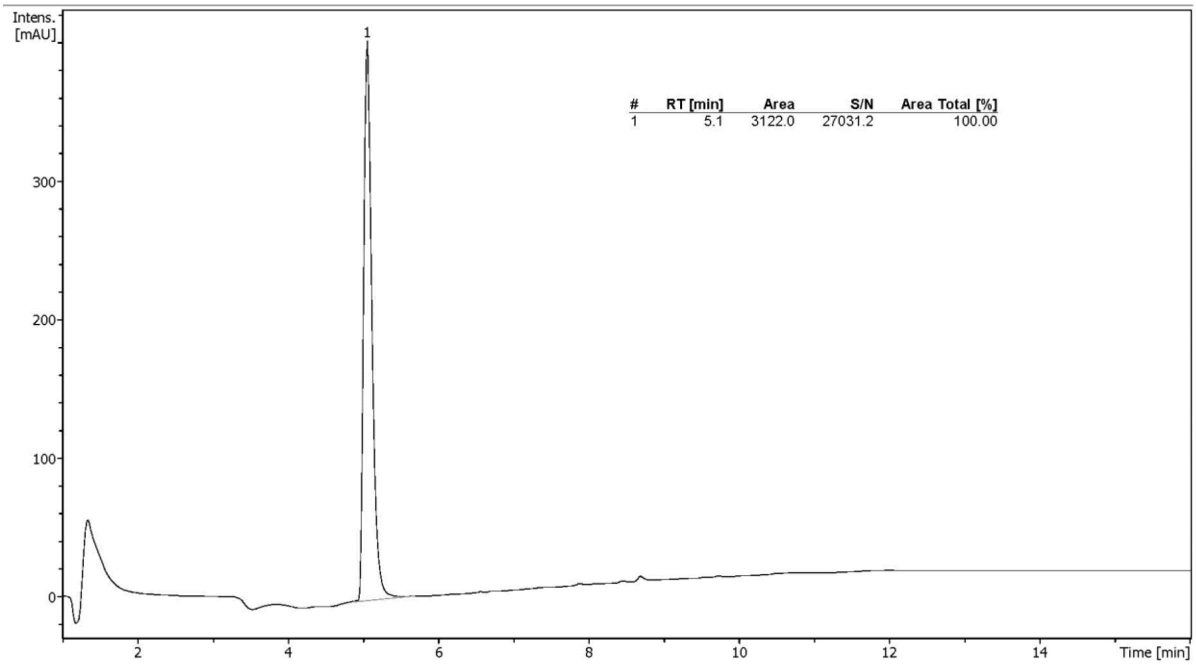

Figure S28. HPLC trace of the compound **4a**.

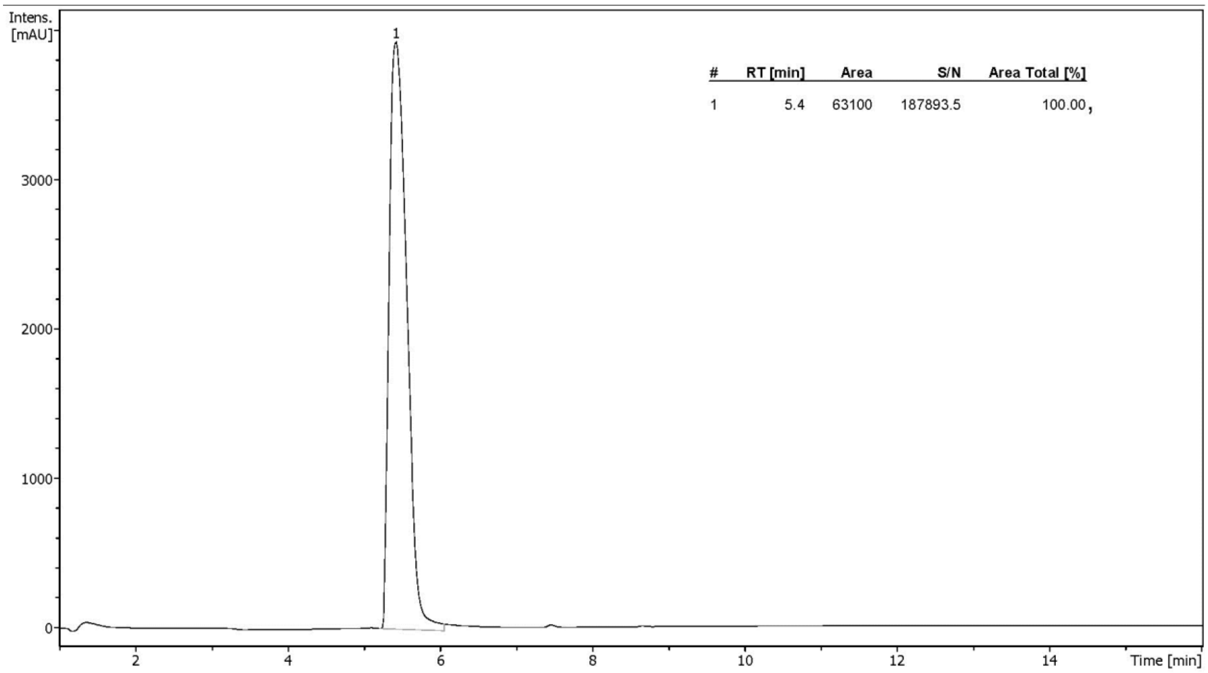

Figure S29. HPLC trace of the compound **4b**.

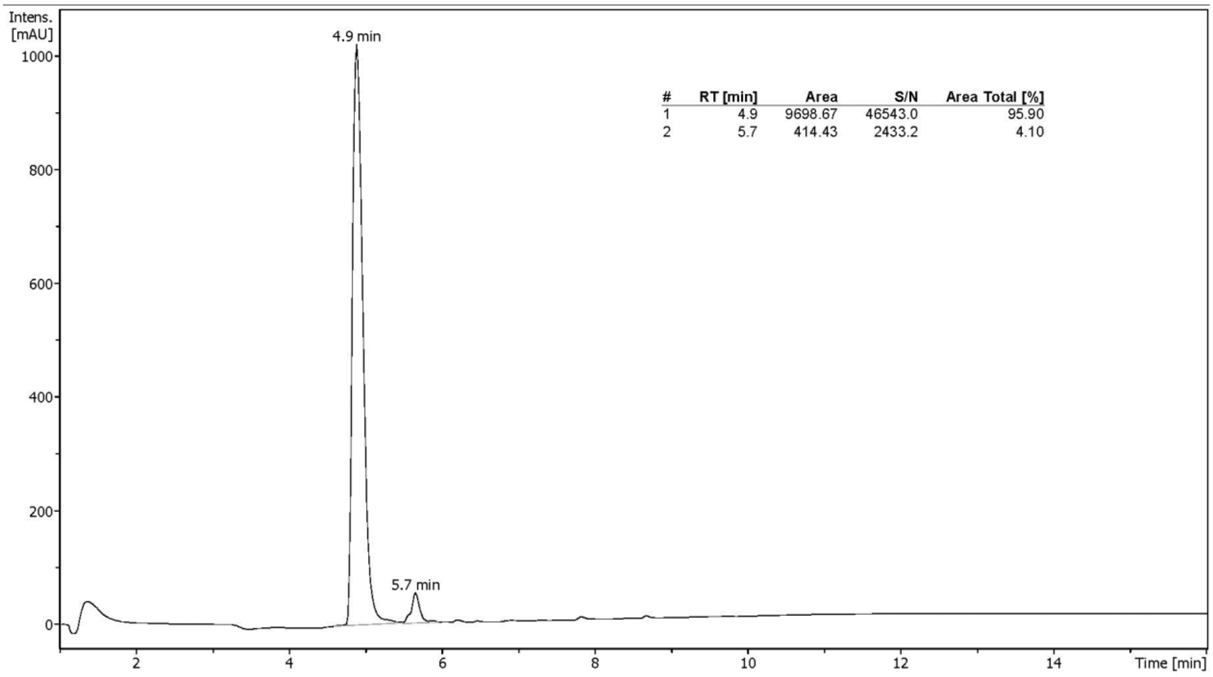

Figure S30. HPLC trace of the compound **4c**.

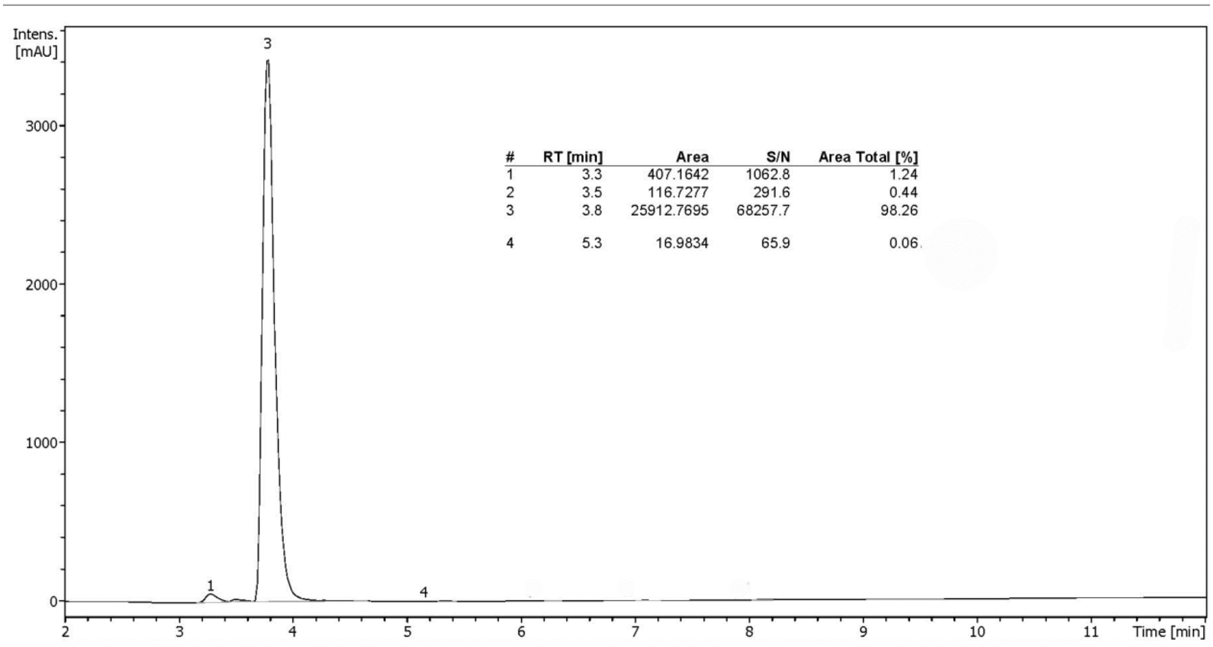

Figure S31. HPLC trace of the compound **4d**.

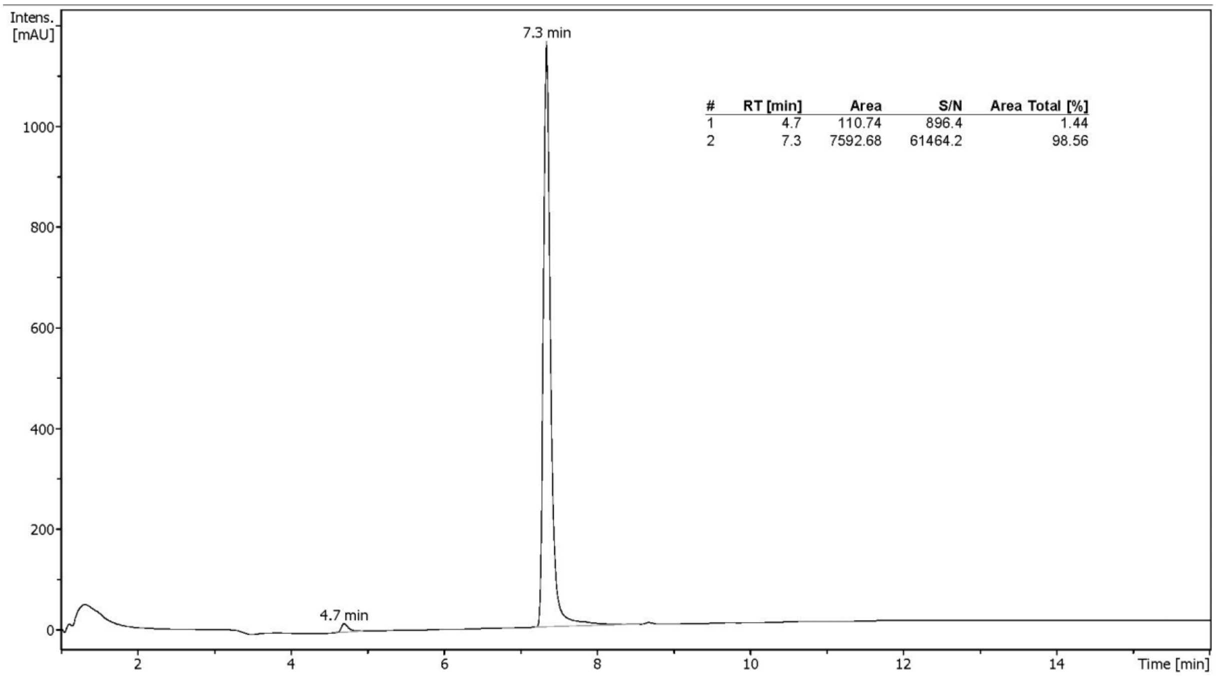

Figure S32. HPLC trace of the compound **5a**.

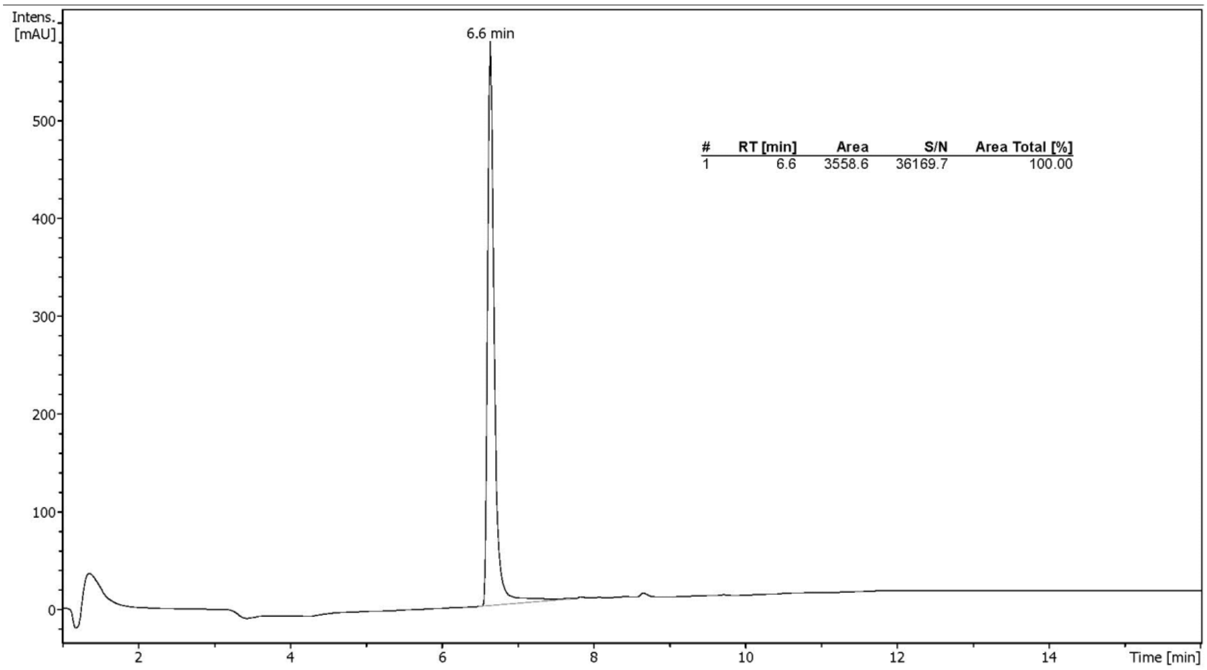

Figure S33. HPLC trace of the compound **5b**.

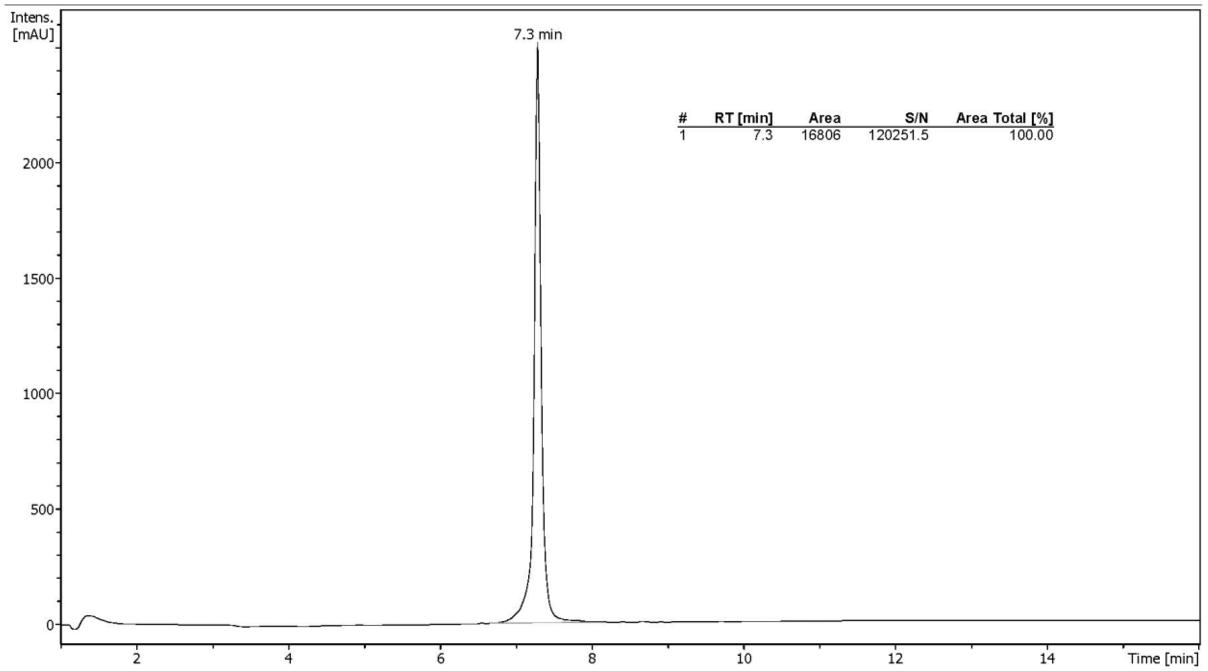

Figure S34. HPLC trace of the compound **6**.

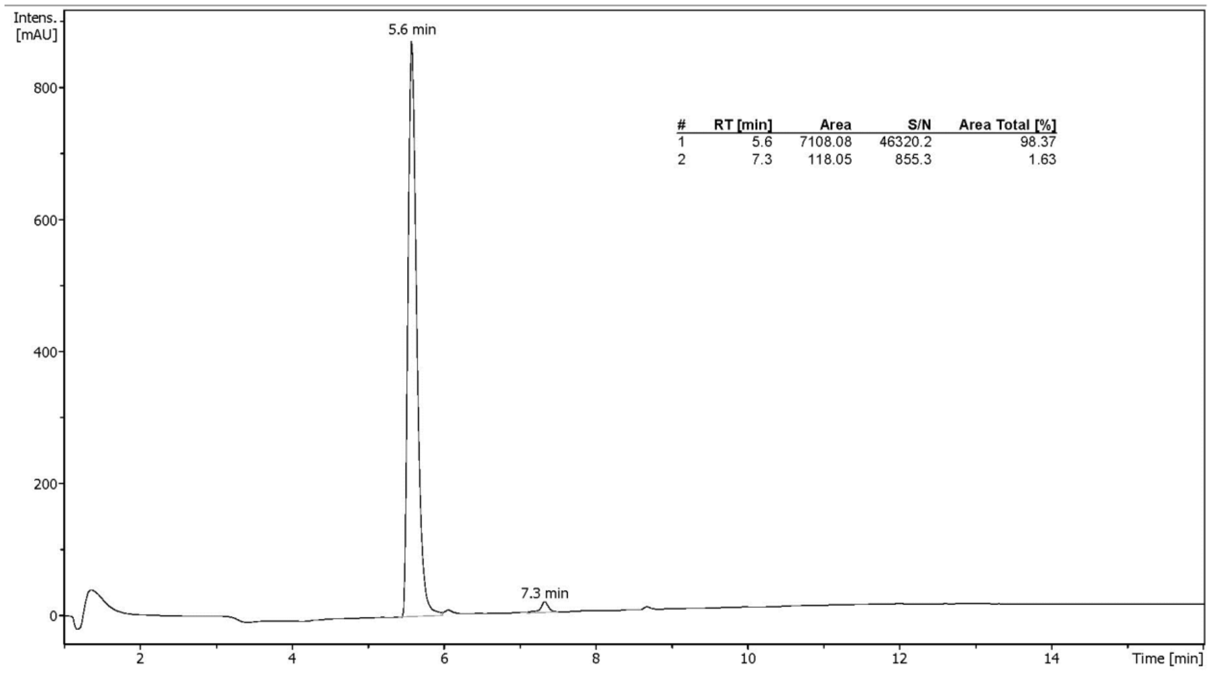

Figure S35. HPLC trace of the compound **7**.

SI References

(1) Šafařík, M.; Moško, T.; Zawada, Z.; Šafaříková, E.; Dračínský, M.; Holada, K.; Šebestík, J. Reactivity of 9-Aminoacridine Drug Quinacrine with Glutathione Limits Its Antiprion Activity. *Chem. Biol. Drug Des.* 2017, *89* (6), 932–942. https://doi.org/10.1111/cbdd.12918.

(2) Puhl, A. C.; Gomes, G. F.; Damasceno, S.; Godoy, A. S.; Noske, G. D.; Nakamura, A. M.; Gawriljuk, V. O.; Fernandes, R. S.; Monakhova, N.; Riabova, O.; Lane, T. R.; Makarov, V.; Veras, F. P.; Batah, S. S.; Fabro, A. T.; Oliva, G.; Cunha, F. Q.; Alves-Filho, J. C.; Cunha, T. M.; Ekins, S. Pyronaridine Protects against SARS-CoV-2 Infection in Mouse. *ACS Infect. Dis.* 2022, *8* (6), 1147–1160. https://doi.org/10.1021/acsinfecdis.2c00091.

(3) Lambros, C.; Vanderberg, J. P. Synchronization of Plasmodium Falciparum Erythrocytic Stages in Culture. *J. Parasitol.* 1979, *65* (3), 418. https://doi.org/10.2307/3280287.

(4) Johnson, J. D.; Dennull, R. A.; Gerena, L.; Lopez-Sanchez, M.; Roncal, N. E.; Waters, N. C. Assessment and Continued Validation of the Malaria SYBR Green I-Based Fluorescence Assay for Use in Malaria Drug Screening. *Antimicrob. Agents Chemother.* 2007, *51* (6), 1926–1933. https://doi.org/10.1128/AAC.01607-06.

(5) Katsuno, K.; Burrows, J. N.; Duncan, K.; van Huijsduijnen, R. H.; Kaneko, T.; Kita, K.; Mowbray, C. E.; Schmatz, D.; Warner, P.; Slingsby, B. T. Hit and Lead Criteria in Drug Discovery for Infectious Diseases of the Developing World. *Nat. Rev. Drug Discov.* 2015, *14* (11), 751–758. https://doi.org/10.1038/nrd4683.

(6) Aguiar, A. C. C.; Pereira, D. B.; Amaral, N. S.; De Marco, L.; Krettli, A. U. Plasmodium Vivax and Plasmodium Falciparum Ex Vivo Susceptibility to Anti-Malarials and Gene Characterization in Rondônia, West Amazon, Brazil. *Malar. J.* 2014, *13* (1), 73. https://doi.org/10.1186/1475-2875-13-73.

(7) Le Manach, C.; Scheurer, C.; Sax, S.; Schleiferböck, S.; Cabrera, D. G.; Younis, Y.; Paquet, T.; Street, L.; Smith, P.; Ding, X. C.; Waterson, D.; Witty, M. J.; Leroy, D.; Chibale, K.; Wittlin, S. Fast in Vitro Methods to Determine the Speed of Action and the Stage-Specificity of Anti-Malarials in Plasmodium Falciparum. *Malar. J.* 2013, *12* (1), 424. https://doi.org/10.1186/1475-2875-12-424.

(8) Fivelman, Q. L.; Adagu, I. S.; Warhurst, D. C. Modified Fixed-Ratio Isobologram Method for Studying In Vitro Interactions between Atovaquone and Proguanil or Dihydroartemisinin against Drug-Resistant Strains of *Plasmodium Falciparum*. *Antimicrob. Agents Chemother.* 2004, *48* (11), 4097–4102. https://doi.org/10.1128/AAC.48.11.4097-4102.2004.

(9) Hand, D. J. Synergy in Drug Combinations; 2000; pp 471–475. https://doi.org/10.1007/978-3-642-58250-9_38.

(10) Peters, W. Drug Resistance in Plasmodium Berghei Vincke and Lips, 1948. I. Chloroquine Resistance. *Exp. Parasitol.* 1965, *17* (1), 80–89. https://doi.org/10.1016/0014-4894(65)90012-3.

(11) Baumans, V.; Brain, P. F.; Brugére, H.; Clausing, P.; Jeneskog, T.; Perretta, G. Pain and Distress in Laboratory Rodents and Lagomorphs. Report of the Federation of European

Laboratory Animal Science Associations (FELASA) Working Group on Pain and Distress Accepted by the FELASA Board of Management November 1992. *Lab. Anim.* 1994, *28* (2), 97–112. https://doi.org/10.1258/002367794780745308.

(12) Okada-Junior, C. Y.; Monteiro, G. C.; Aguiar, A. C. C.; Batista, V. S.; de Souza, J. O.; Souza, G. E.; Bueno, R. V.; Oliva, G.; Nascimento-Júnior, N. M.; Guido, R. V. C.; Bolzani, V. S. Phthalimide Derivatives with Bioactivity against *Plasmodium Falciparum* : Synthesis, Evaluation, and Computational Studies Involving *Bc* 1 Cytochrome Inhibition. *ACS Omega* 2018, *3* (8), 9424–9430. https://doi.org/10.1021/acsomega.8b01062.

(13) Carter, M. D.; Phelan, V. V; Sandlin, R. D.; Bachmann, B. O.; Wright, D. W. Lipophilic Mediated Assays for Beta-Hematin Inhibitors. *Comb. Chem. High Throughput Screen.* 2010, *13* (3), 285–292. https://doi.org/10.2174/138620710790980496.

(14) Paquet, T.; Le Manach, C.; Cabrera, D. G.; Younis, Y.; Henrich, P. P.; Abraham, T. S.; Lee, M. C. S.; Basak, R.; Ghidelli-Disse, S.; Lafuente-Monasterio, M. J.; Bantscheff, M.; Ruecker, A.; Blagborough, A. M.; Zakutansky, S. E.; Zeeman, A.-M.; White, K. L.; Shackleford, D. M.; Mannila, J.; Morizzi, J.; Scheurer, C.; Angulo-Barturen, I.; Martínez, M. S.; Ferrer, S.; Sanz, L. M.; Gamo, F. J.; Reader, J.; Botha, M.; Dechering, K. J.; Sauerwein, R. W.; Tungtaeng, A.; Vanachayangkul, P.; Lim, C. S.; Burrows, J.; Witty, M. J.; Marsh, K. C.; Bodenreider, C.; Rochford, R.; Solapure, S. M.; Jiménez-Díaz, M. B.; Wittlin, S.; Charman, S. A.; Donini, C.; Campo, B.; Birkholtz, L.-M.; Hanson, K. K.; Drewes, G.; Kocken, C. H. M.; Delves, M. J.; Leroy, D.; Fidock, D. A.; Waterson, D.; Street, L. J.; Chibale, K. Antimalarial Efficacy of MMV390048, an Inhibitor of *Plasmodium* Phosphatidylinositol 4-Kinase. *Sci. Transl. Med.* 2017, *9* (387). https://doi.org/10.1126/scitranslmed.aad9735.

(15) Murithi, J. M.; Owen, E. S.; Istvan, E. S.; Lee, M. C. S.; Ottilie, S.; Chibale, K.; Goldberg, D. E.; Winzeler, E. A.; Llinás, M.; Fidock, D. A.; Vanaerschot, M. Combining Stage Specificity and Metabolomic Profiling to Advance Antimalarial Drug Discovery. *Cell Chem. Biol.* 2020, *27* (2), 158-171.e3. https://doi.org/10.1016/j.chembiol.2019.11.009.
